# Supplementary material for: Loss of hepatocyte PI3Kα reduces hepatocellular carcinoma and hepatocyte proliferation in association with altered lipid metabolism
Source: JHEP Rep. 2026 Apr 15;8(7):101847. doi: 10.1016/j.jhepr.2026.101847 (PMC13310634; doi:10.1016/j.jhepr.2026.101847)
Supplement: Multimedia component 1 [file mmc1.pdf]

Loss of hepatocyte PI3Kα protects mice from hepatocellular carcinoma in association with altered lipid metabolism gene expression

Barbara Becattini, Claudia Sardi, Bart Edelbroek, Amit Chand Gupta, Toshima Parris, Khalil Helou, Giovanni Solinas

Table of contents

Supplementary figures 2

Fig. S1

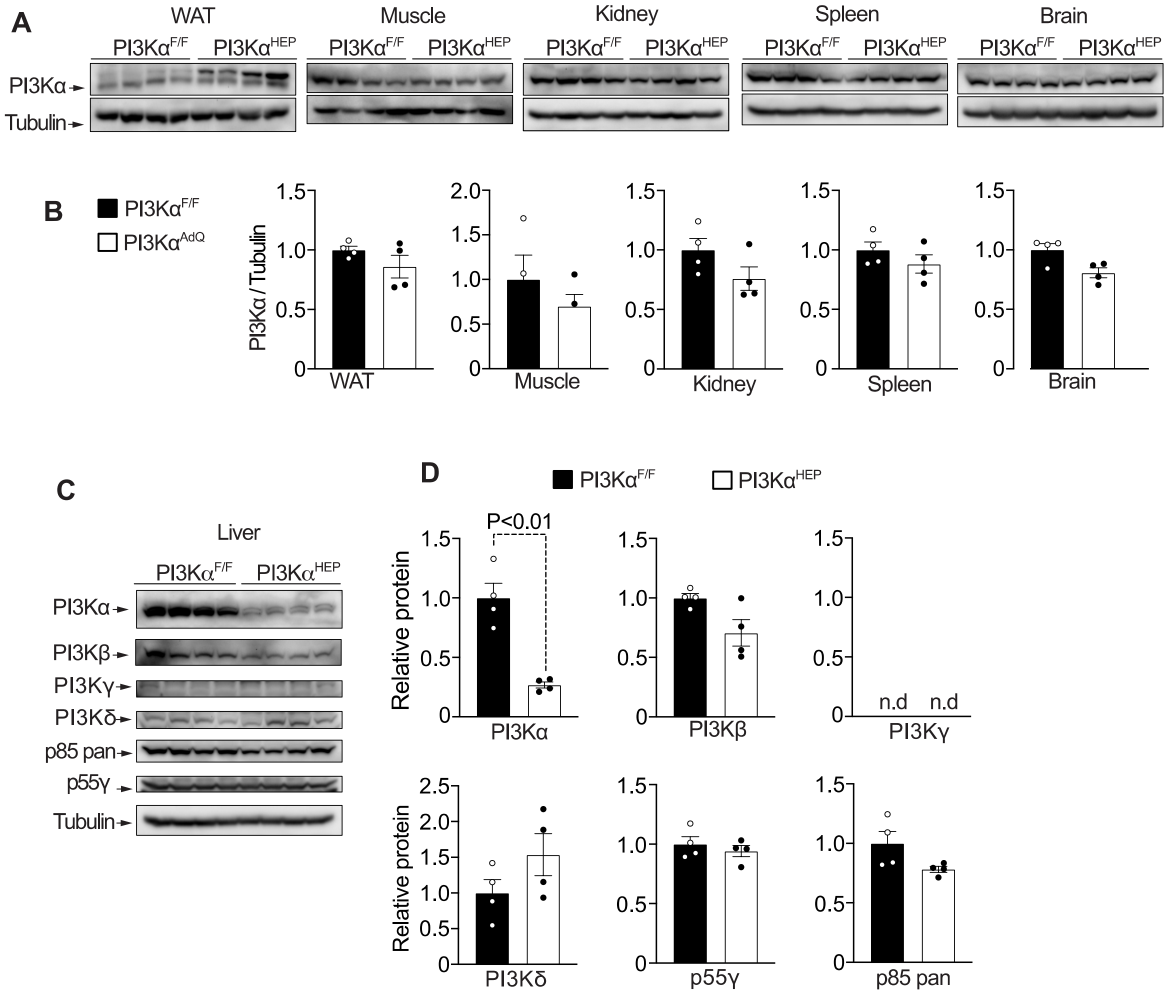

Fig. S1. (Related to Fig. 1). Characterization of PI3Kα deletion in PI3Kα^{Hep} mice.

(A) Immunoblots analysis of PI3Kα abundances in white adipose tissue (WAT) muscle, kidney, spleen, and brain. (B) Quantifications of the blots in A. (C) Immunoblot analysis of different class-1 PI3K in the liver of PI3Kα^{Hep} mice and PI3Kα$^{F/F}$ mice. (D) Quantifications of the blots in C. Data are represented as means, and error bars indicate standard errors. Statistical analysis was performed using Mann-Whitney.

n=4 mice per group.

Fig. S2

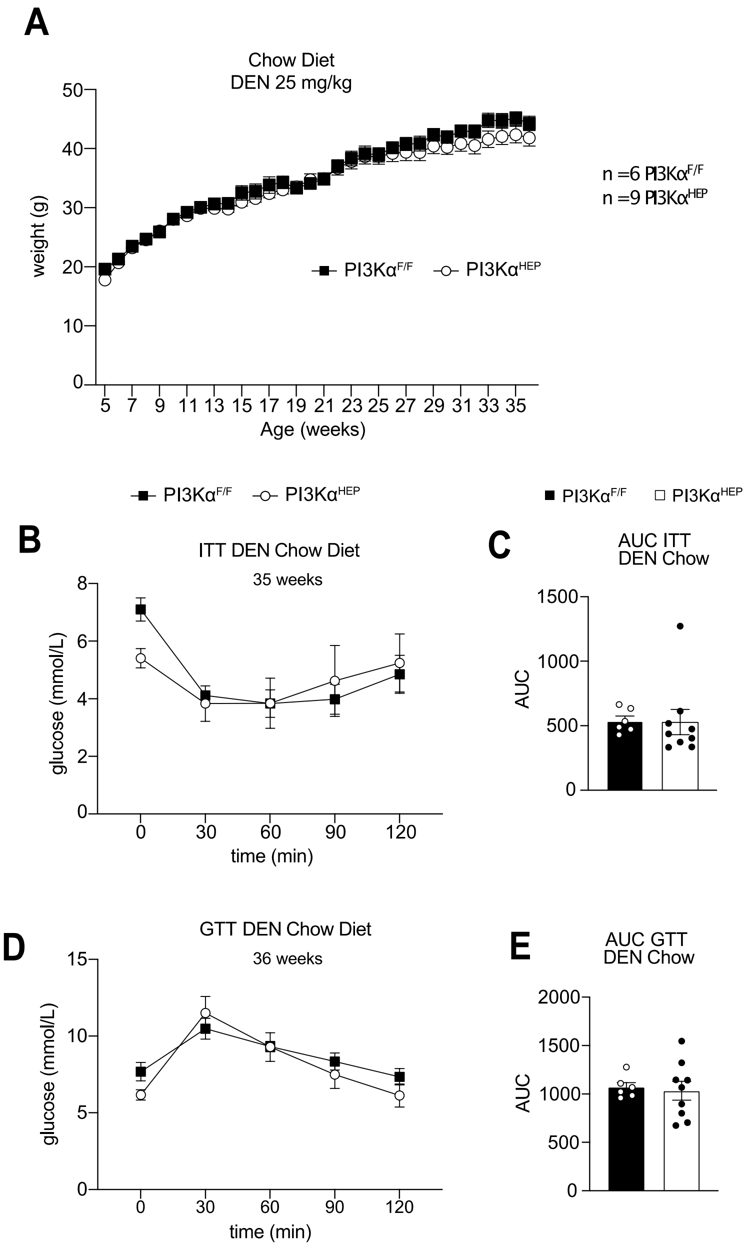

Fig. S2. (Related to Fig. 1). Loss of hepatocyte PI3Kα has a negligible impact on growth and glucose homeostasis in DEN-injected mice fed a chow diet.

(A) Growth curve of $PI3K\alpha^{F/F}$ and $PI3K\alpha^{HEP}$ mice kept on a chow diet. (B) Insulin tolerance test (ITT) of the mice from A at the age of 35 weeks. (C) Area under the curves of the ITT in B. (D) Glucose tolerance test (GTT) of the mice in A at the age of 36 weeks. (E) Area under the curve of the GTT in D. n=6-9 mice

Data are represented as means, and error bars indicate standard errors. Statistical analysis was performed using Mann-Whitney for C and E, and two-way ANOVA for A,B, D.

Fig. S3

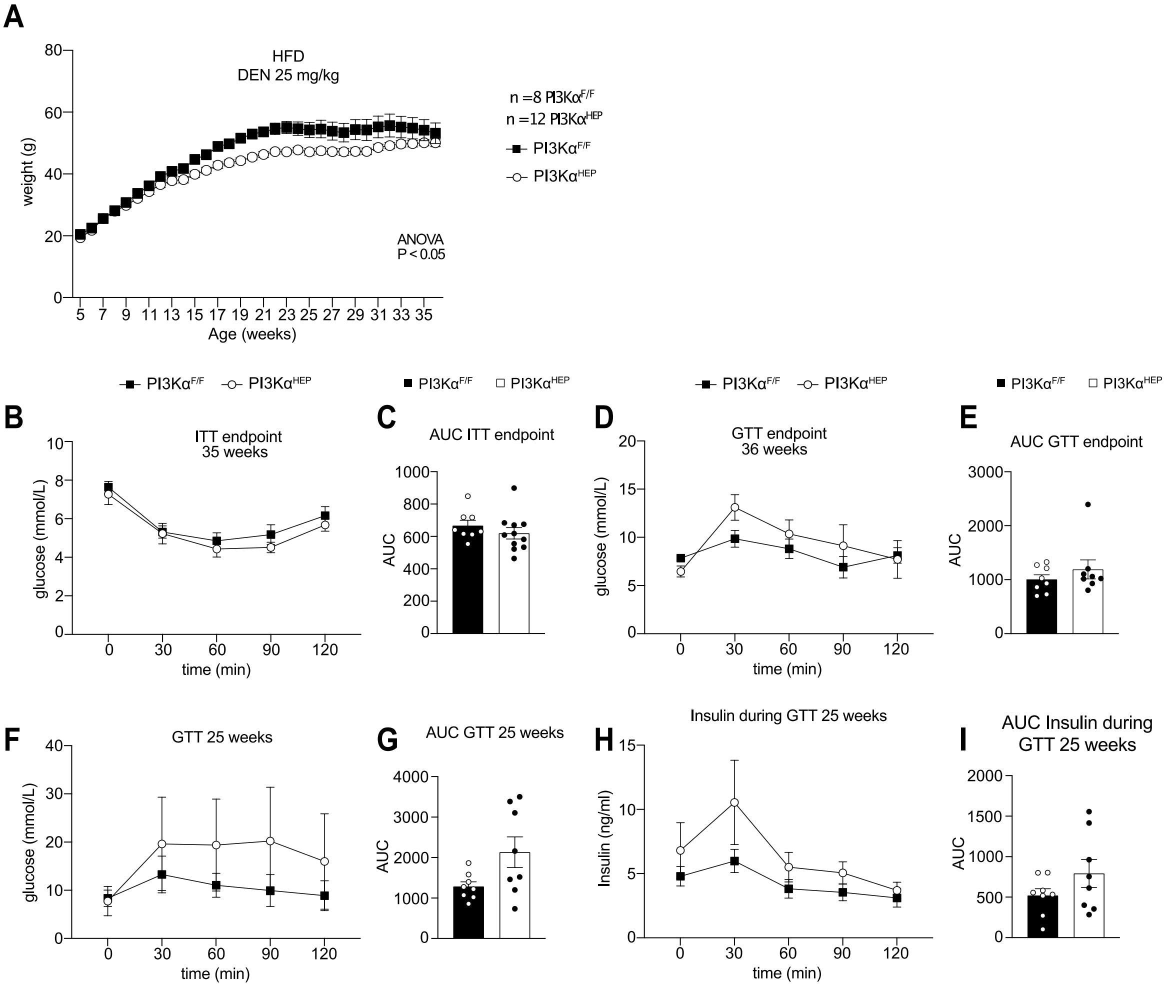

Fig. S3. (Related to Fig. 1) Loss of hepatocyte PI3Kα has a mild impact on growth and glucose homeostasis in DEN-injected mice fed an obesogenic HFD.

(A) Growth curve of PI3Kα$^{F/F}$ and PI3Kα^{HEP} mice kept on an HFD. (B) Insulin tolerance test (ITT) of the mice from A at the age of 35 weeks. (C) Area under the curves of the ITT in B. (D) Glucose tolerance test (GTT) of the mice in A at the age of 36 weeks. (E) Area under the curve of the GTT in D. (F) GTT of the mice in A at the age of 25 weeks. (G) Area under the curve of the GTT in F. (H) Serum insulin during the GTT in F. (I) Area under the curve of the insulin levels in H. n=8-12 mice for A, n=8 mice for B-I. Data are represented as means, and error bars indicate standard errors. Statistical analysis was performed using Mann-Whitney for C, E,D. I and two-way ANOVA for A,B, D, F, H.

Fig. S4

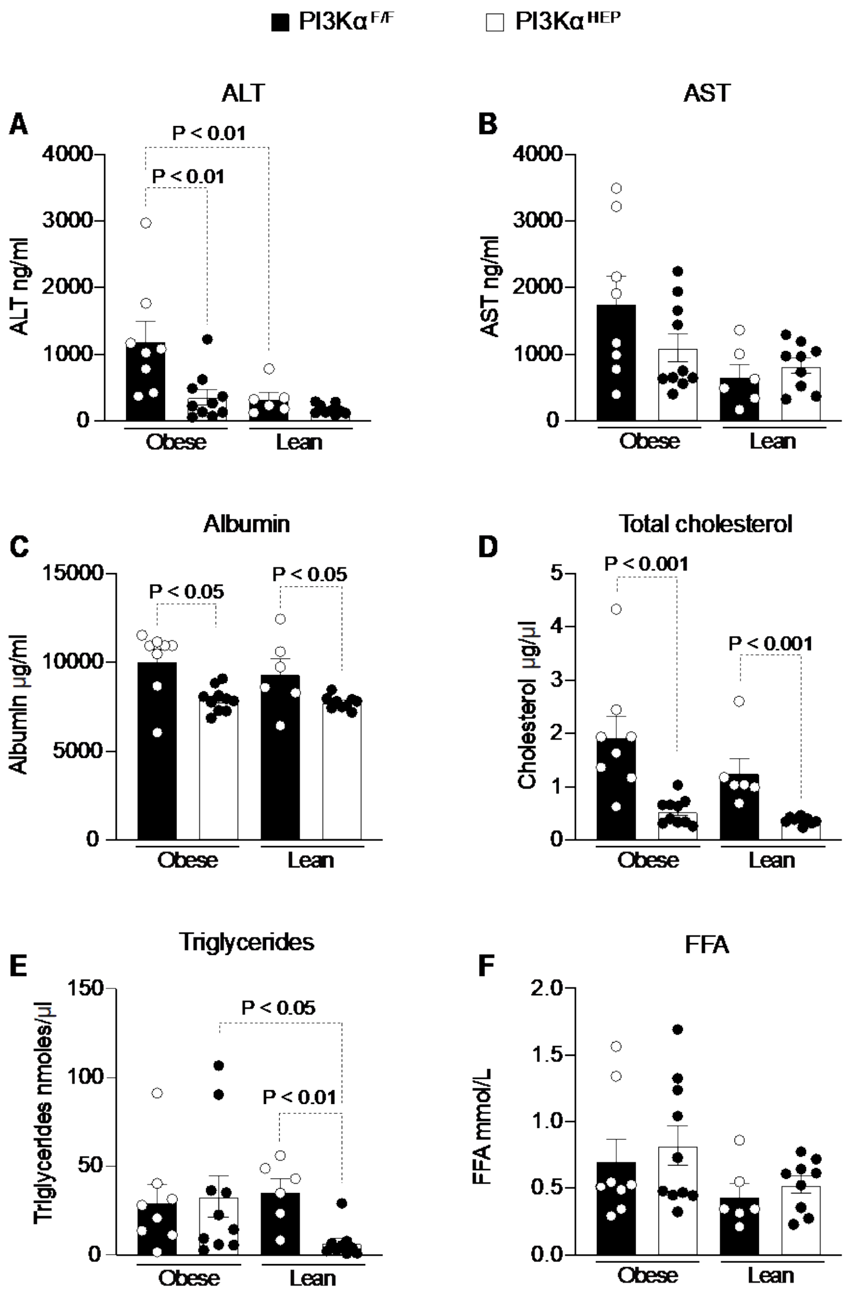

Fig. S3. (Related to Fig. 1) Liver enzymes and circulating lipids.

(A) Serum abundance of Alanine Aminotransferase (ALT). (B) Serum abundance of Aspartate Aminotransferase (AST). (C) Serum abundance of Albumin. (D) Serum abundance of total cholesterol. (E) Serum abundance of triglycerides. (F) Serum abundance of Free fatty acids (FFA). n=6-10 mice per group. Data are represented as means, and error bars indicate standard errors. Statistical analysis was performed using Mann-Whitney.

Fig. S5

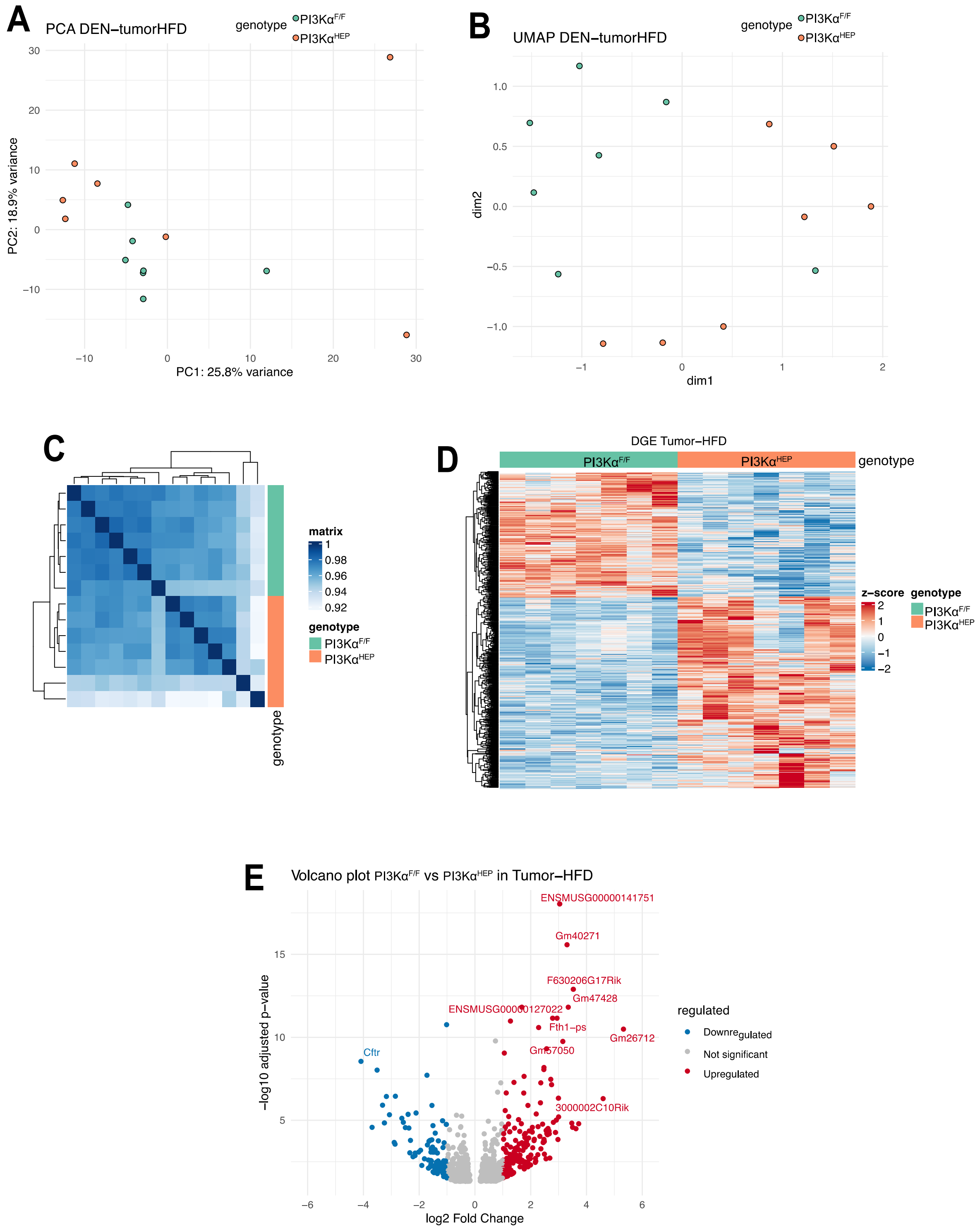
A
PCA DEN−tumorHFD
genotype
PI3Kα^F/F
PI3Kα^HEP
PC2: 18.9% variance
PC1: 25.8% variance
B
UMAP DEN−tumorHFD
dim1
dim2
C
matrix
genotype
D
DGE Tumor−HFD
z−score
E
Volcano plot PI3Kα^F/F vs PI3Kα^HEP in Tumor−HFD
ENSMUSG00000141751
Gm40271
F630206G17Rik
Gm47428
ENSMUSG00000127022
Fth1−ps
Gm26712
Gm57050
Cftr
3000002C10Rik
regulated
Downregulated
Not significant
Upregulated
−log10 adjusted p−value
log2 Fold Change

Fig. S5. (Related to Fig. 3) Dimensional analysis and differentially expressed genes in HCC from PI3K$\alpha^{F/F}$ and PI3Kα^{HEP} mice kept on an HFD.

(A) Principal component analysis (PCA) of mRNA Sequencing data from HCC of PI3K$\alpha^{F/F}$ and PI3Kα^{HEP} mice kept on an HFD from Figure 3. (B) Uniform Manifold Approximation and Projection (UMAP) analysis of mRNA sequencing data from HCC of PI3K$\alpha^{F/F}$ and PI3Kα^{HEP} mice kept on an HFD from Figure 3. (C) Clustered Spearman correlation matrix from the data above. (D) Heatmap showing z-scores of differentially expressed genes from HCC of PI3K$\alpha^{F/F}$ and PI3Kα^{HEP} mice kept on an HFD from Figure 3. Each row contains expression of one differentially expressed gene (n = 890, adj. p-value < 0.05). Each column represents the expression in one biological replicate (n = 7). (E) Volcano plot of the differentially expressed genes in D, marked as downregulated (log2 fold change < -1) or upregulated (log2 fold change > 1). The top 10 genes are labeled, based on the product of the absolute fold change and $-\log_{10}$ adj. p-value. Statistical analysis was performed using BH-adjusted Wald test
n=7 mice per genotype.

Fig. S6

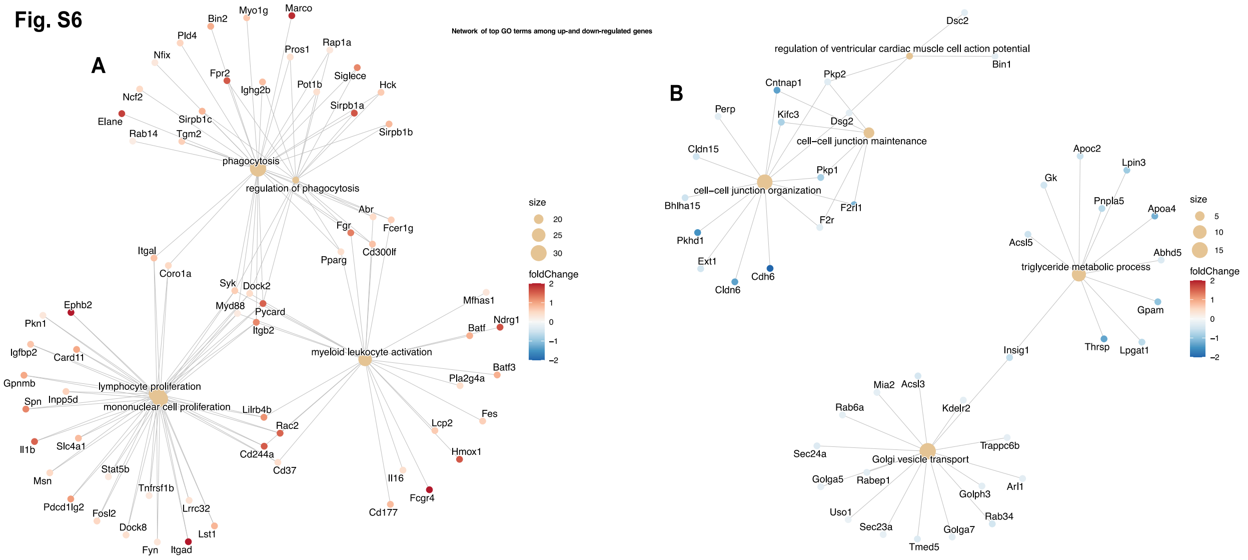

Fig. S6. (Related to Fig. 3) Network of top gene ontology terms

(A) Network of the most significant gene ontology terms of upregulated genes and their associated upregulated genes in HCC of PI3Kα^{HEP} mice kept on an HFD from Figure 3. Gene nodes are colored according to $\log_2$ fold change.

(B) Network of the most significant gene ontology terms of downregulated genes and their associated downregulated genes in HCC of PI3Kα^{HEP} mice kept on an HFD from Figure 3. Gene nodes are colored according to $\log_2$ fold change. n=7 mice per genotype.

Fig. S7

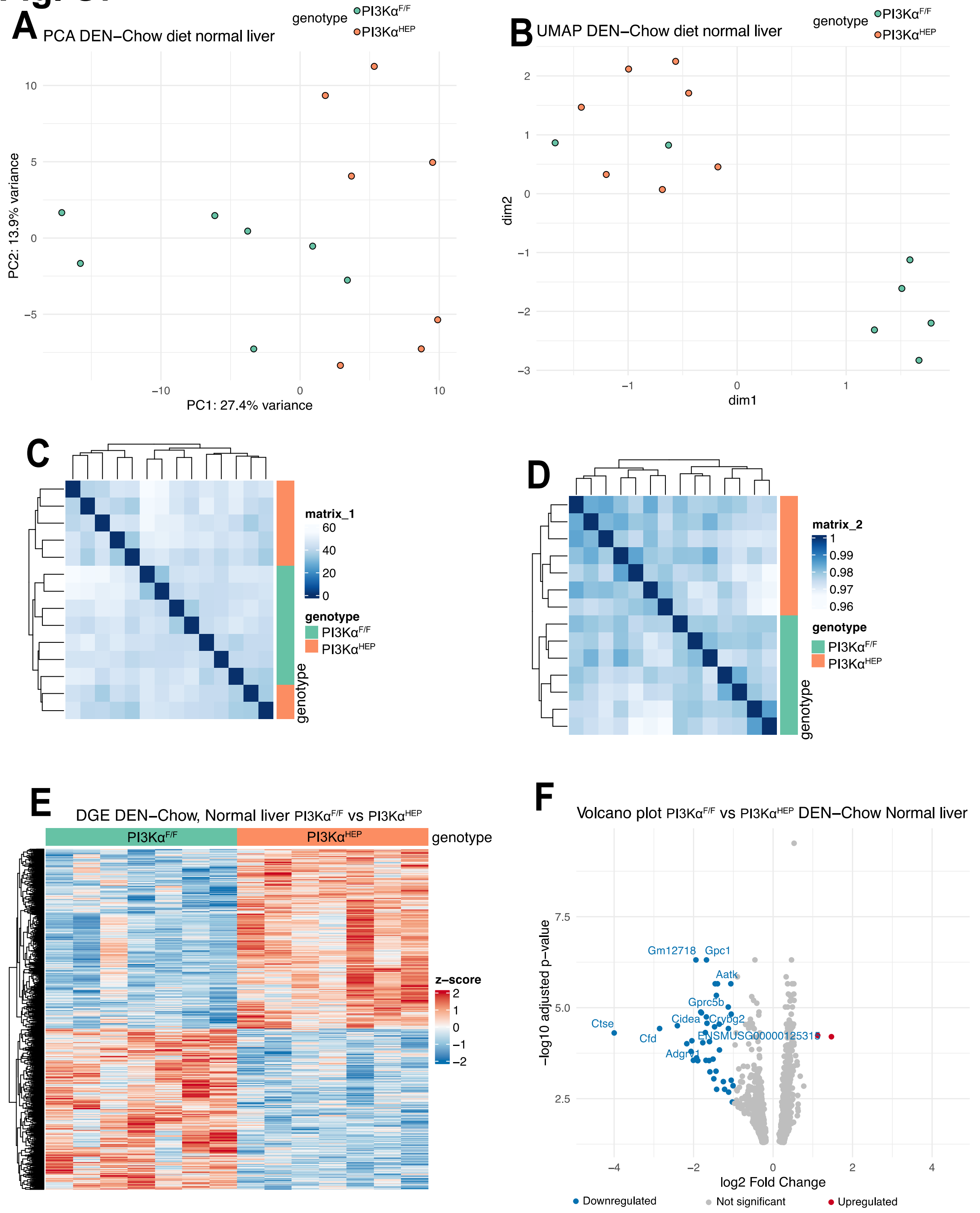

Fig. S7. (Related to Fig. 4) Dimensional analysis and differentially expressed genes in normal livers from PI3Kα$^{F/F}$ and PI3Kα^{HEP} mice kept on a chow diet.

(A) Principal component analysis (PCA) of mRNA Sequencing data from normal livers of PI3K$\alpha^{F/F}$ and PI3Kα^{HEP} mice kept on a chow diet from Figure 4.
(B) Uniform Manifold Approximation and Projection (UMAP) analysis of mRNA Sequencing data from above.
(C) Clustered Euclidian distance matrix from the data above.
(D) Clustered Spearman correlation matrix from the data above.
(E) Heatmap showing z-scores of differentially expressed genes from normal liver tissue of PI3K$\alpha^{F/F}$ and PI3Kα^{HEP} mice kept on chow diet from Figure 4. Each row contains expression of one differentially expressed gene (n = 1418, adj. p-value < 0.05). Each column represents the expression in one biological replicate (n = 7).
(F) Volcano plot of the differentially expressed genes in E, marked as downregulated (log2 fold change < -1) or upregulated (log2 fold change > 1). The top 10 genes are labeled, based on the product of the absolute fold change and $-\log_{10}$ adj. p-value.
Statistical analysis was performed using BH-adjusted Wald test. n=7 mice per genotype.

Fig. S8

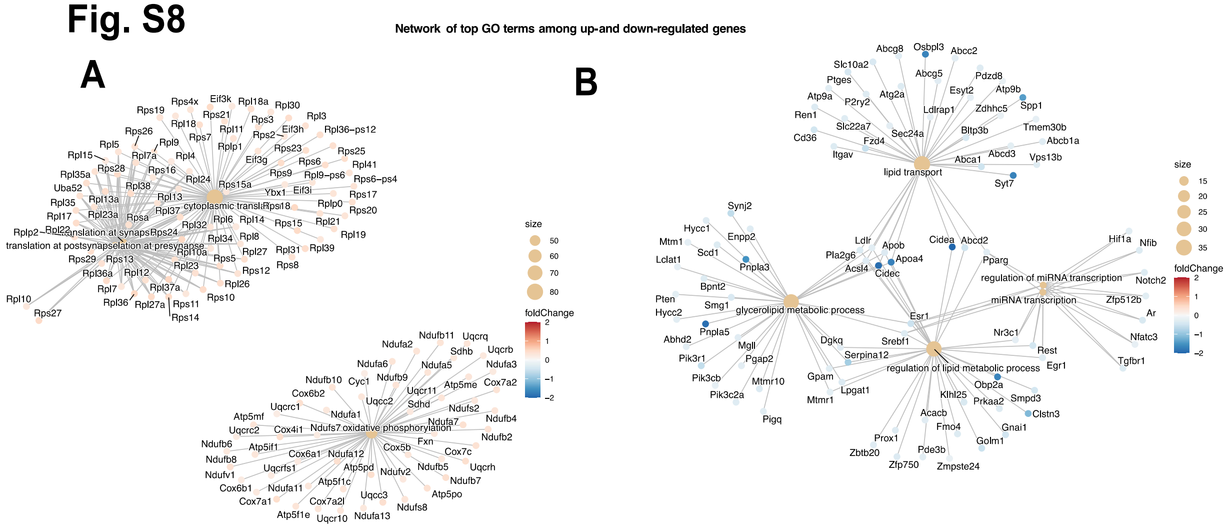

Fig. S8. (Related to Fig. 4) Network of top gene ontology terms (Chow diet)

(A) Network of the most significant gene ontology terms of upregulated genes and their associated upregulated genes in the normal liver of PI3Kα^{HEP} mice kept on a chow diet from Figure 4. Gene nodes are colored according to $\log_2$ fold change.

(B) Network of the most significant gene ontology terms of downregulated genes and their associated downregulated genes in the normal liver of PI3Kα^{HEP} mice kept on a chow diet from Figure 4. Gene nodes are colored according to $\log_2$ fold change.

n=7 mice per genotype.

Fig. S9

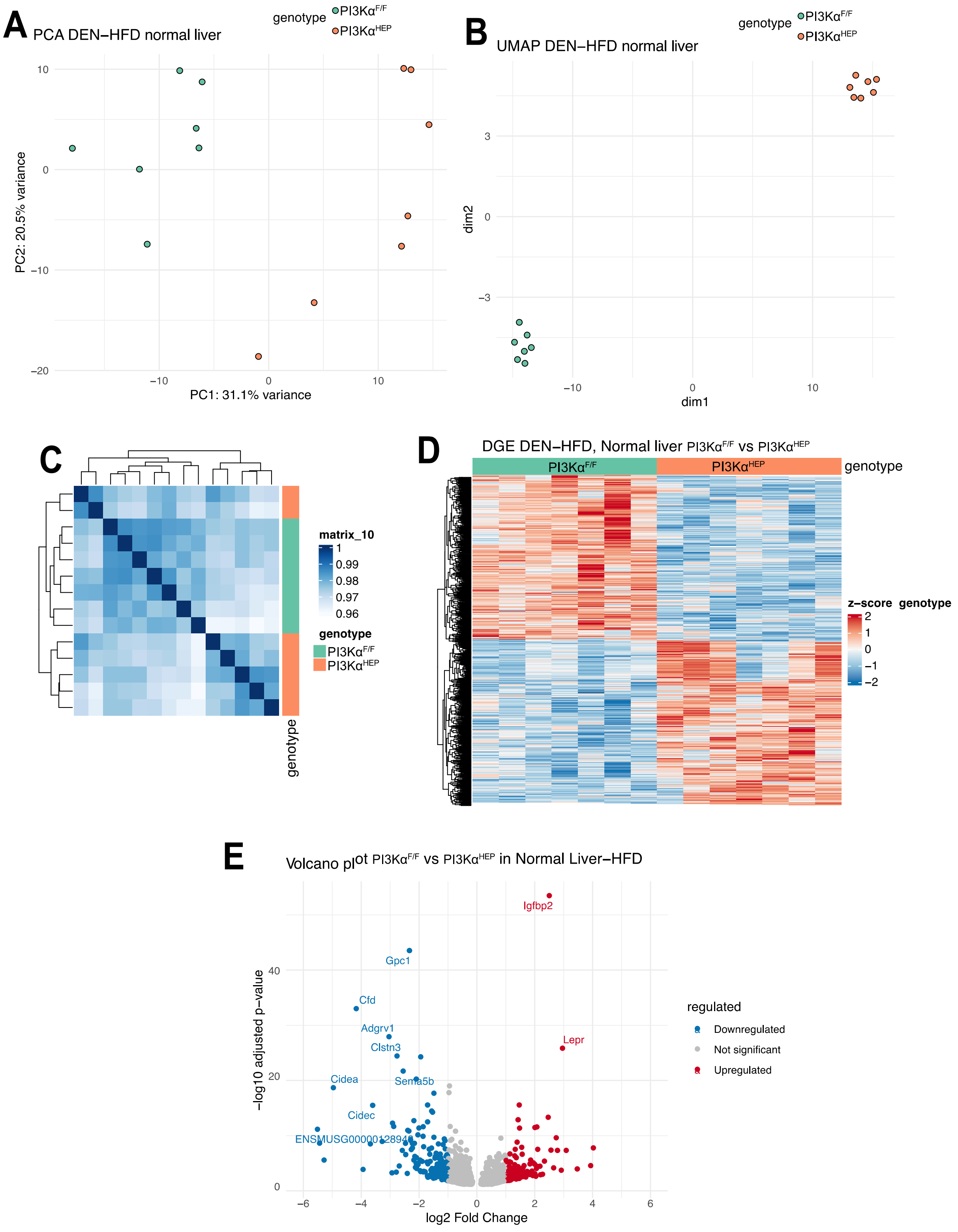

A
PCA DEN−HFD normal liver
genotype
PI3Kα F/F
PI3Kα HEP
PC1: 31.1% variance
PC2: 20.5% variance
B
UMAP DEN−HFD normal liver
dim1
dim2
C
matrix_10
genotype
D
DGE DEN−HFD, Normal liver PI3Kα F/F vs PI3Kα HEP
z−score
E
Volcano plot PI3Kα F/F vs PI3Kα HEP in Normal Liver−HFD
log2 Fold Change
−log10 adjusted p−value
regulated
Downregulated
Not significant
Upregulated
Igfbp2
Gpc1
Cfd
Adgrv1
Clstn3
Lepr
Cidea
Sema5b
Cidec

Fig. S9. (Related to Fig. 4) Dimensional analysis and differentially expressed genes in normal livers from PI3K$\alpha^{F/F}$ and PI3Kα^{HEP} mice kept on HFD

(A) Principal component analysis (PCA) of mRNA Sequencing data from normal livers of PI3K$\alpha^{F/F}$ and PI3Kα^{HEP} mice kept on an obesogenic HFD from Figure 4.

(B) Uniform Manifold Approximation and Projection (UMAP) analysis of mRNA Sequencing data from above.

(C) Clustered Spearman correlation matrix from the data above.

(D) Heatmap showing z-scores of differentially expressed genes from normal livers of PI3K$\alpha^{F/F}$ and PI3Kα^{HEP} mice kept on HFD from Figure 4. Each row contains expression of one differentially expressed gene (n = 1814, adj. p-value < 0.05). Each column represents the expression in one biological replicate (n = 7).

(E) Volcano plot of the differentially expressed genes in D, marked as downregulated (log2 fold change < -1) or upregulated (log2 fold change > 1). The top 10 genes are labeled, based on the product of the absolute fold change and $-\log_{10}$ adj. p-value. Statistical analysis was performed using BH-adjusted Wald test. n=7 mice per genotype.

Fig. S10

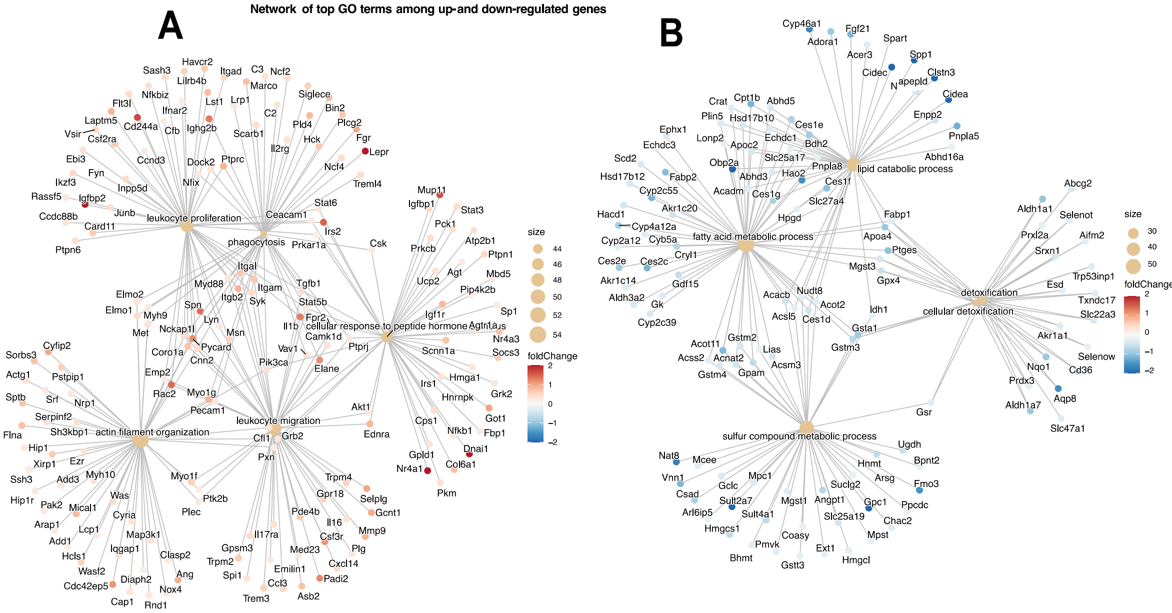

Fig. S10. (Related to Fig. 4) Network of top gene ontology terms (HFD)

(A) Network of the most significant gene ontology terms of upregulated genes and their associated upregulated genes in the normal liver of PI3Kα^{HEP} mice kept on HFD from Figure 4. Gene nodes are colored according to $\log_2$ fold change.

(B) Network of the most significant gene ontology terms of downregulated genes and their associated downregulated genes in the normal liver of PI3Kα^{HEP} mice kept on HFD from Figure 4. Gene nodes are colored according to $\log_2$ fold change.

n=7 mice per genotype.

Fig. S11

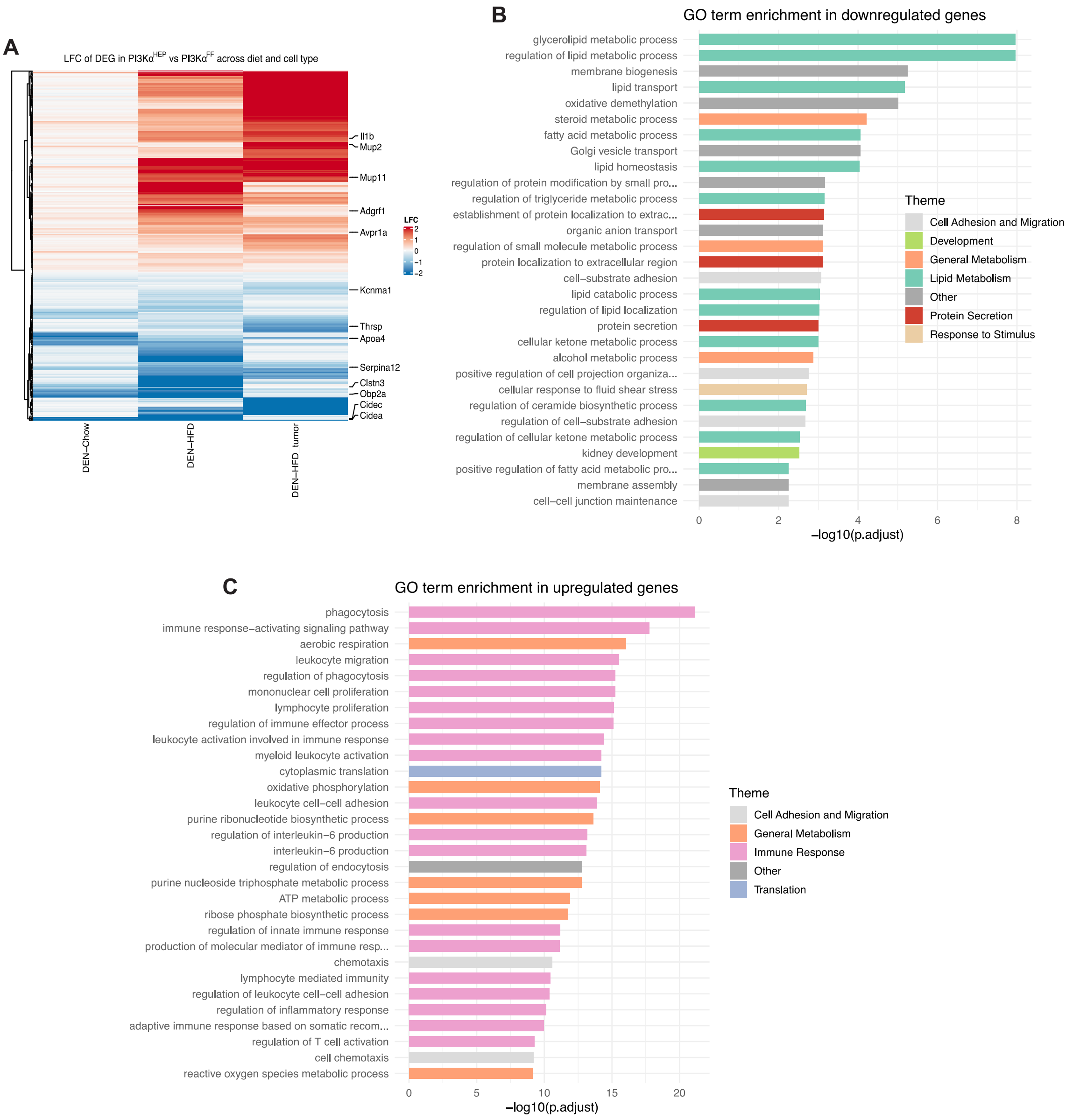

Fig. S11. (Related to Fig. 3, 4) Analysis of gene expression, all samples compared. (A) Heat map of differentially expressed genes in normal liver and tumor in chow and HFD. Log2 fold change (LFC) of expression in PI3Kα^{Hep} vs PI3Kα$^{F/F}$ across diet and tissue type. Significantly regulated genes (p-adj < 0.05) with a minimum effect size (|LFC| > 1) from the combined analysis were plotted. Genes which belong to the ‘regulation of lipid metabolism’ GO-term (GO:0019216) are labeled with their gene symbol.

(B) Gene ontology (GO) term enrichment analysis of all significantly downregulated genes (p-adj < 0.05) from the combined analysis in A. The top 30 most significant GO terms are shown, colored by overarching theme

(C) Gene ontology (GO) term enrichment analysis of all significantly upregulated genes (p-adj < 0.05) from the combined analysis in A. The top 30 most significant GO terms are shown, colored by overarching theme. Statistical analysis was performed using BH-adjusted hypergeometric test p-values.

Fig. S12

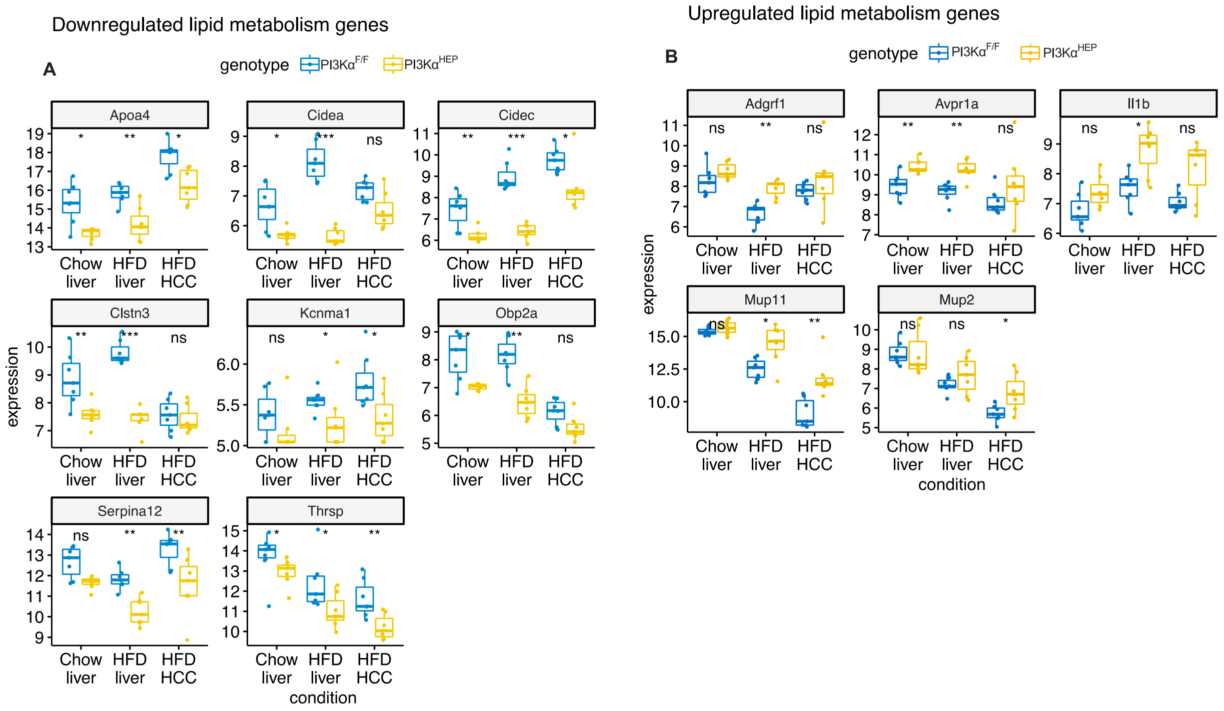

Fig. S12. (Related to Fig. 3, 4) Expression of significantly regulated lipid metabolism genes across diet and tissue types.

Expression of significantly regulated genes (p-adj < 0.05) with a minimum effect size (|LFC| > 1) across diet and tissue types from the combined analysis. Boxplots show the expression per condition in PI3K$\alpha^{F/F}$ (blue) and PI3Kα^{Hep} (yellow). The points show the expression for each of the biological replicates for the given gene (n=7). Statistical significance for the pairwise comparisons per condition shown above the boxplots (Wilcoxon rank sum test; ns = p ≥ 0.5, * = p < 0.05, ** = p < 0.01, *** = p < 0.001). Downregulated genes shown in A, and upregulated genes in B.

Fig. S13

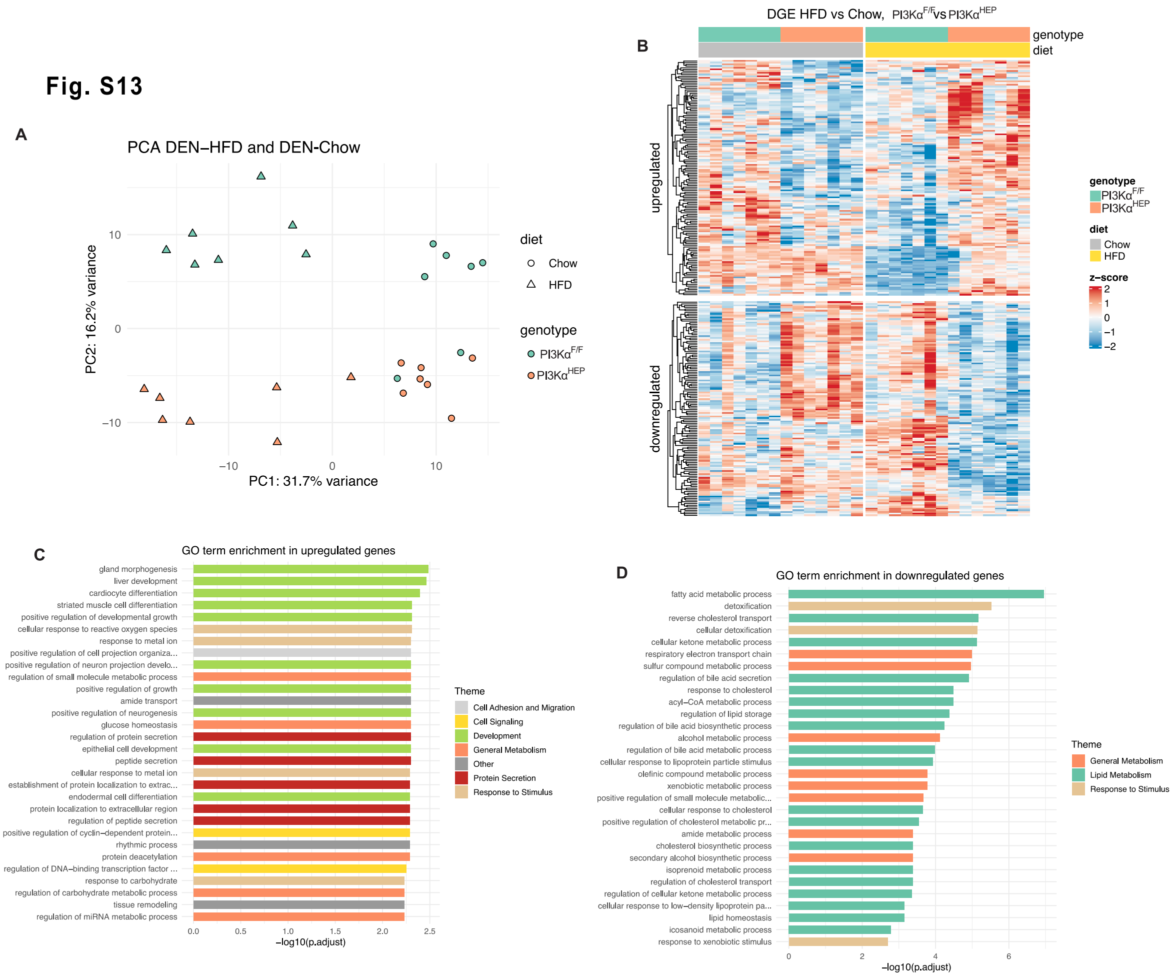

Fig. S13. (Related to Fig. 3, 4) Impact of diet on PI3Kα dependent gene regulation in normal liver.

(A) Principal component analysis of normal liver tissue from mice kept on chow diet (circles) or HFD (triangles). Points colored by genotype.

(B) Heatmap of differentially expressed genes dependent on PI3Kα and diet. Z-scores shown for all significant genes (p-adj < 0.05) with a minimum 1.5-fold up or downregulation in the interaction term.

(C) Gene ontology (GO) term enrichment analysis of the upregulated genes from B. The top 30 most significant GO terms are shown, colored by overarching theme.

(D) Gene ontology (GO) term enrichment analysis of the downregulated genes from B. The top 30 most significant GO terms are shown, colored by overarching theme. Statistical analysis by BH-adjusted hypergeometric test p-values.

Fig. S14

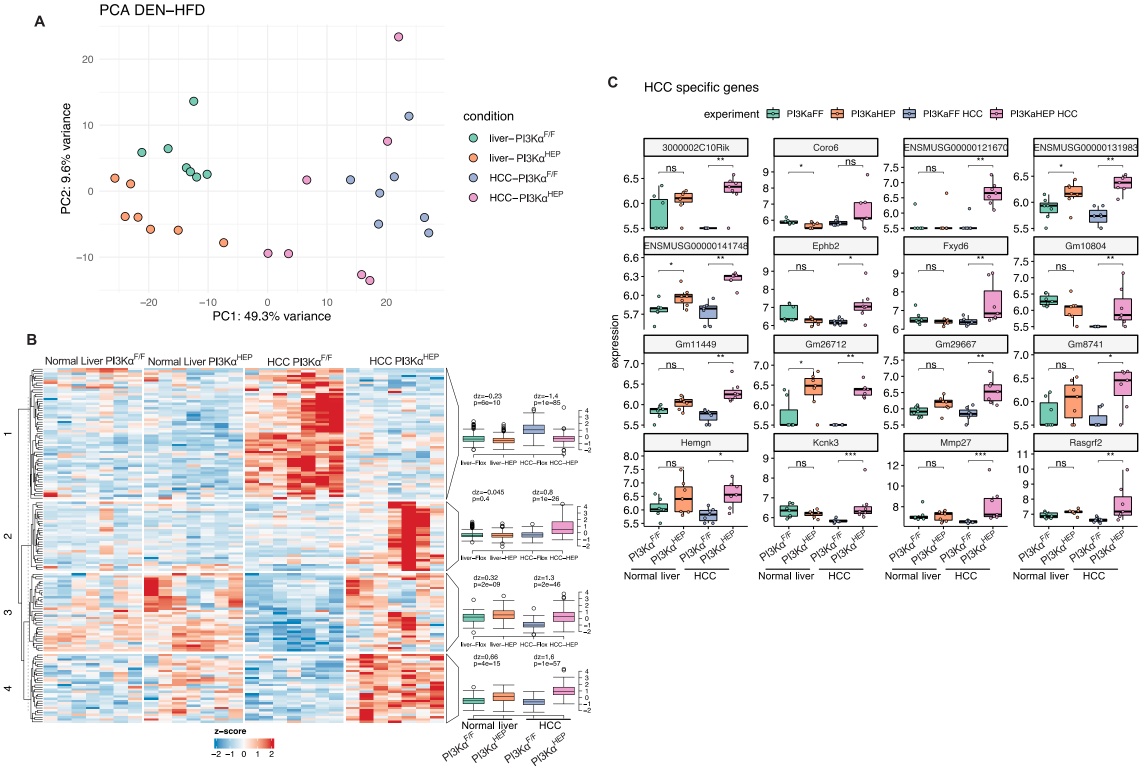

Fig. S14. (Related to Fig. 3, 4) Analysis of gene expression, comparison of normal liver and tumor from PI3Kα^{Hep} and PI3Kα$^{F/F}$ mice kept on HFD.

(A) Principal component analysis of normal liver tissue and HCC from mice kept on HFD, colored by condition.

(B) Heatmap of differentially expressed genes in response to PI3Kα in HCC but not normal liver tissue. Genes with p-adj < 0.05 and |LFC| > 1 in HCC, and p-adj > 0.1 or |LFC| < 0.2 in normal liver tissue were defined as specifically regulated in HCC. Z-scores were hierarchically clustered. The boxplots to the right show the z-scores for the cluster, for each of the conditions. The p-value (pairwise Wilcox test) and relative change in z-score (dz) are shown for the pairwise comparisons (normal liver PI3Kα$^{F/F}$ vs PI3Kα^{Hep} and HCC PI3Kα$^{F/F}$ vs PI3Kα^{Hep}).

(C) Expression of top 16 genes with the greatest LFC difference between normal liver and HCC from B. Statistical significance for the pairwise comparisons per condition shown above the boxplots (Wilcoxon rank sum test; ns = p ≥ 0.5, * = p < 0.05, ** = p < 0.01, *** = p < 0.001).
