# Supplementary material for: Loss of hepatocyte PI3Kα reduces hepatocellular carcinoma and hepatocyte proliferation in association with altered lipid metabolism
Source: JHEP Rep. 2026 Apr 15;8(7):101847. doi: 10.1016/j.jhepr.2026.101847 (PMC13310634; doi:10.1016/j.jhepr.2026.101847)
Supplement: Multimedia component 2 [file mmc2.docx]

**JHEP Reports**

**CTAT methods**

Tables for a “Complete, Transparent, Accurate and Timely account” (CTAT) are now mandatory for all revised submissions. The aim is to enhance the reproducibility of methods.

- Only include the parts relevant to your study
- Refer to the CTAT in the main text as ‘Supplementary CTAT Table’
- Do not add subheadings
- Add as many rows as needed to include all information
- Only include one item per row

**If the CTAT form is not relevant to your study, please outline the reasons why:**

|  |
| --- |

- 1. **Antibodies**

| **Name** | **Citation** | **Supplier** | **Cat no.** | **Clone no.** |
| --- | --- | --- | --- | --- |
| AKT Phospho (Thr308) |  | Cell Signaling | 4056 |  |
| AKT Phospho (Ser373) XP |  | Cell Signaling | 4060 |  |
| AKT |  | Cell Signaling | 9272 |  |
| Erk 1/2 (p44/42 MAPK) Phospho(Thr202/Tyr204) XP |  | Cell Signaling | 9101 |  |
| Erk 1/2 (p44/42 MAPK) |  | Cell Signaling | 9102 |  |
| Tubulin α/β |  | Cell Signaling | 2148 |  |
| PI3Kα |  | Cell Signaling | 4249 |  |
| PI3Kβ |  | Cell Signaling | 3011 |  |
| PI3Kδ |  | Millipore | 04-401 |  |
| PI3Kγ |  | a gift of Prof. Matthias Wymann University of Basel | Russian |  |
| PI3K p85 |  | Cell Signaling | 4257 |  |
| PI3K p55γ |  | Abcam | Ab238509 |  |
| Ki67 |  | Cell Signaling | 12202 |  |
| Horse Anti-Rabbit IgG |  | Vector Laboratories | BA-1100 |  |

- 1. **Cell lines**

| **Name** | **Citation** | **Supplier** | **Cat no.** | **Passage no.** | **Authentication test method** |
| --- | --- | --- | --- | --- | --- |
|  |  |  |  |  |  |

- 1. **Organisms**

| **Name** | **Citation** | **Supplier** | **Strain** | **Sex** | **Age** | **Overall n number** |
| --- | --- | --- | --- | --- | --- | --- |
| PI3Kα^Flox^ |  | Provided by Victoria Rotter Sopasakis |  | Males | 2-36 weeks | As indicated in figure legends |
| PI3Kα^HEP^ |  | Provided by Victoria Rotter Sopasakis |  | Males | 2-36 weeks | As indicated in figure legends |

- 1. **Sequence based reagents**

| **Name** | **Sequence** | **Supplier** |
| --- | --- | --- |
| Cyclophilin; Genbank: NM_009807  Primer Forward ATGGTCAACCCCACCGTGT | Eurofins Genomics | N/A |
| Cyclophilin; Genbank: NM_009807  Primer Reverse TTTCTGCTGTCTTTGGAACTTTGTC | Eurofins Genomics | N/A |
| Cyclin D1; GenBank: NM_007631.2  Primer Forward GGGTGGGTTGGAAATGAAC | Eurofins Genomics | N/A |
| Cyclin D1; GenBank: NM_007631.2  Primer Reverse TCCTCTCCAAAATGCCAGAG | Eurofins Genomics | N/A |

- 1. **Biological samples**

| **Description** | **Source** | **Identifier** |
| --- | --- | --- |
|  |  |  |

- 1. **Deposited data**

| **Name of repository** | **Identifier** | **Link** |
| --- | --- | --- |
| Mendeley Data | Loss of Hepatocyte PI3K$\alpha$Reduces Hepatocellular Carcinoma in Association with Altered Lipid Metabolism Gene Expression | DOI: 10.17632/g6fndymznk.1 |

- 1. **Software**

| **Software name** | **Manufacturer** | **Version** |
| --- | --- | --- |
| Image Lab software (version 5.2.1) | Bio-Rad | http://www.bio-rad.com/en-us/ product/image-lab-software |
| Graph Pad Prism 7.0 | Graph Pad Software | N/A |
| AxioVision Software | Imaging Sofware | Carl Zeiss Microscopy |

- 1. **Other (*e.g*. drugs, proteins, vectors etc.)**

| Isofluorane | Baxter | KDG 9623 |
| --- | --- | --- |
| Insulin | Humalog Lilly | VL7510 |
| Glucose | Acros | 410955000 |
| N-Nitrosodiethylamine (DEN) | Sigma | 73861 |
| HGF | Sigma | SRP3300 |
| EGF | Sigma | E1257 |
| BSA | Fisher Scientific | BP9702 |
| ECL-anti rabbit IgG HRP | GE Healthcare | NA934V |
| Type I Collagen, from rat tail | Corning | BD#354236 |
| Collagenase type IV | Sigma | C5138 |
| Normal Horse Serum | Vector laboratories | S-2000 |
| H_2_O_2_ | Sigma | 216763 |
| 3,3-diaminobenzidine | Sigma | D-5905 |
| Entellan | Merck | 1.07961.0100 |
| GSK2636771 (Inhibitor PI3Kβ) | SelleckChem | S8002 |
| PIK294 (Inhibitor PI3Kδ) | MedChem Express | HY-10303 |
| IPI549 (Inhibitor PI3Kγ) | MedChem Express(SelleckChem) | HY-100716 |
| TUNEL Assay | Roche | 12 156 792 910 |
| DNase I | Roche | 04 716 728 001 |
| ImPROM-II Reverse Transcriptase | Promega | A3803 |
| Syber Green (qPCR Mix) | Bio-Rad | 1725270 |
| Immobilon Western Chemiluminescence HRP Substrate | Millipore | WBLKS0500 |
| Vectastain ABC reagent | Vector laboratories | PK-4000 |
| Hematoxylin Solution, Gill No.3 | Sigma | GHS316 |
| Eosin | Sigma | HT110116 |
| High Fat Diet | Bio-Serv, Germany | F3282 |
| Glucose Strips | Contour Next | Ascensia |
| Ultra Sensitive Mouse Insulin ELISA Kit | Crystal Chem | 90080 |
| Non-Esterified Fatty Acid (NEFA) | Fujifilm | 999-34691, 991-34891 |
| Mouse AST ELISA Kit | Abcam | AB263882 |
| Mouse ALT ELISA Kit |  | AB282882 |
| Mouse Albumin ELISA Kit | Abcam | AB207620 |
| Triglyceride Assay Kit - Quantification | Abcam | AB65336 |
| Cholesterol/ Cholesteryl Ester Assay Kit - Quantitation | Abcam | AB65359 |

- 1. **Please provide the details of the corresponding methods author for the manuscript:**

|  |
| --- |

**2.0 Please confirm for randomised controlled trials all versions of the clinical protocol are included in the submission. These will be published online as supplementary information.**

|  |
| --- |
