# Supplementary material for: Loss of hepatocyte PI3Kα reduces hepatocellular carcinoma and hepatocyte proliferation in association with altered lipid metabolism
Source: JHEP Rep. 2026 Apr 15;8(7):101847. doi: 10.1016/j.jhepr.2026.101847 (PMC13310634; doi:10.1016/j.jhepr.2026.101847)
Supplement: Multimedia component 4 [file mmc4.pdf]

JHEP|Reports

Loss of hepatocyte PI3Kα reduces hepatocellular carcinoma and hepatocyte proliferation in association with altered lipid metabolism

Authors

Barbara Becattini, Claudia Sardi, Bart Edelbroek, …, Toshima Parris, Khalil Helou, Giovanni Solinas

Correspondence

barbara.becattini@wlab.gu.se (B. Becattini), giovanni.solinas@wlab.gu.se (G. Solinas).

Graphical abstract

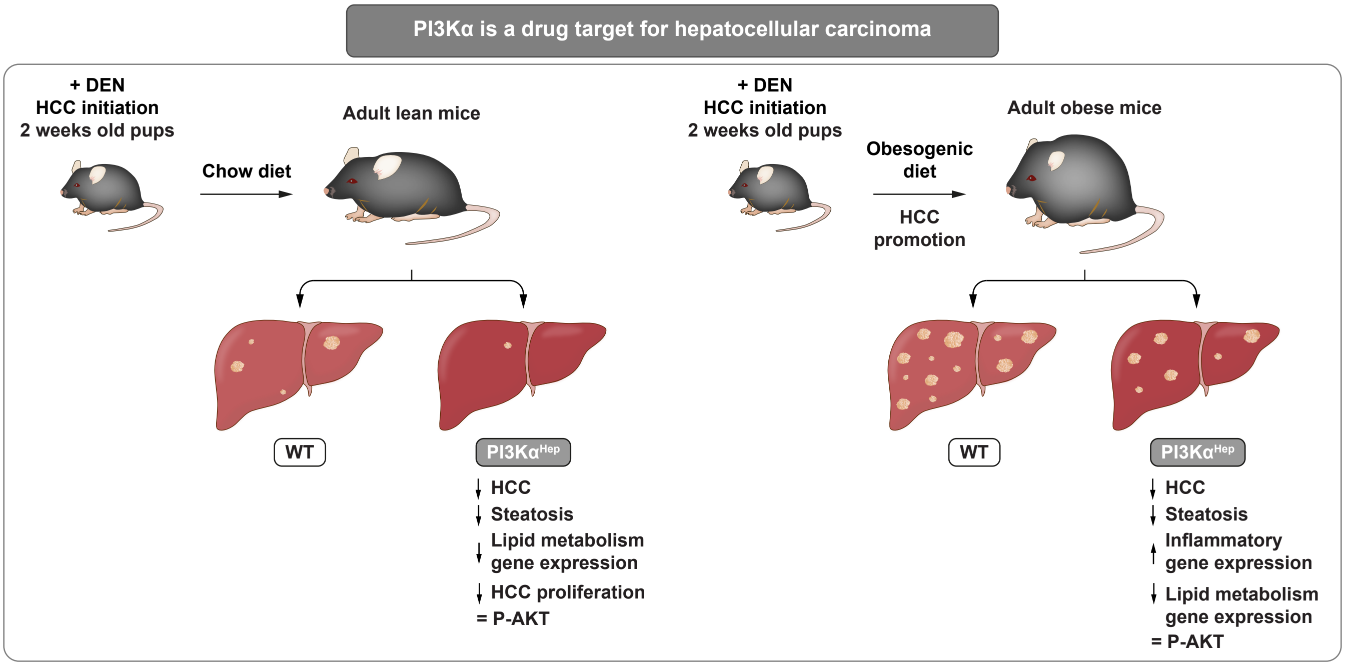

Highlights:

- Loss of PI3Kα in hepatocytes reduces HCC in mice.
- Loss of PI3Kα in hepatocytes reduces carcinoma and hepatocyte proliferation independently of AKT phosphorylation.
- Loss of PI3Kα in hepatocytes alters the expression of genes implicated in lipid metabolism and prevents the formation of lipid droplets.

Impact and implications:

Class-1 PI3Ks are frequently activated in tumors, but complete inhibition of PI3K signaling is associated with liver damage and HCC. We showed that selective ablation of the PI3Kα isoform in hepatocytes drastically reduced HCC development in mice injected with a hepatocarcinogen. This phenotype was associated with reduced hepatocyte proliferation and a gene expression signature indicative of altered lipid metabolism and reduced lipid droplet formation. Overall, our results indicate PI3Kα as a promising drug target for the treatment of HCC.

https://doi.org/10.1016/j.jhepr.2026.101847

© 2026 The Author(s). Published by Elsevier B.V. on behalf of European Association for the Study of the Liver (EASL). This is an open access article under the CC BY license (http://creativecommons.org/licenses/by/4.0/).

Loss of hepatocyte PI3Kα reduces hepatocellular carcinoma and hepatocyte proliferation in association with altered lipid metabolism

Barbara Becattini[1,*], Claudia Sardi[1], Bart Edelbroek[2], Amit Chand Gupta[1], Toshima Parris[3,4], Khalil Helou[3,4], Giovanni Solinas[1,*]

JHEP Reports **2026**. vol. 8 | 1–16

Check for updates

Background & Aims: Hepatocellular carcinoma (HCC) is a disease with an increasing incidence and a high mortality rate. Thus, targeted therapies for HCC are urgently needed. Phosphoinositide 3-kinase (PI3K)-AKT-mTORC1 signaling is frequently induced in solid tumors and is associated with tumor progression and with the most aggressive type of HCC. However, complete inhibition of all PI3K isoforms is unlikely to achieve a favorable therapeutic index for HCC treatment because of on-target side effects. In this study, we determined the role of hepatocyte PI3Kα activity in HCC.

Methods: We investigated the role of hepatocyte PI3Kα in HCC using conditional knockout mice in the *N*-nitrosodiethylamine (DEN) plus high-fat diet (HFD) model of HCC.

Results: Mice lacking PI3Kα in hepatocytes were protected from DEN-induced HCC (n = 8–17, p <0.005). PI3Kα in hepatocytes was dispensable for AKT phosphorylation in HCC and normal liver (n = 4). It was also dispensable for AKT phosphorylation in hepatocytes during compensatory proliferation following acute administration of the hepatocarcinogen (n = 3–4). AKT phosphorylation induced by hepatocyte growth factor (HGF) and epidermal growth factor (EGF) was mediated by redundant PI3Kα and PI3Kβ activities (n = 3–4). Nonetheless, mice lacking hepatocyte PI3Kα showed reduced HCC proliferation and reduced hepatocyte proliferation acutely induced by DEN and by HGF and EGF (n = 3, p <0.05). This phenotype was associated with a gene expression signature indicating altered lipid metabolism and reduced formation of lipid droplets (n = 7).

Conclusions: Together, these results indicate PI3Kα as a promising target for the treatment of HCC.

© 2026 The Author(s). Published by Elsevier B.V. on behalf of European Association for the Study of the Liver (EASL). This is an open access article under the CC BY license (http://creativecommons.org/licenses/by/4.0/).

Introduction

Hepatocellular carcinoma (HCC) is one of the most lethal cancers and is common worldwide and, thus, targeted therapies are needed.[1] Phosphoinositide 3-kinase (PI3K) signaling is frequently activated in metastatic solid tumors[2] and HCC, and is associated with tumor progression and with the most aggressive types of HCC.[3] Insulin potently induces PI3Kα and PI3Kβ activities in hepatocytes,[4] and constitutive activation of insulin-PI3K-AKT signaling in hepatocytes by transplantation of pancreatic islets in the livers of diabetic rats causes HCC.[5] Furthermore, insulin signaling is aberrant in HCC cell lines.[6] In mice, expression of constitutively active PI3Kα mutants,[7] active AKT mutants,[8] or deletion of the phosphatidylinositol (3,4,5)-trisphosphate phosphatase (PTEN)[9] is sufficient to cause HCC. Therefore, targeted inhibition of PI3K activity could be a potential therapeutic approach for HCC. Nonetheless, pan-inhibition of PI3K activity is associated with liver damage and severe hyperinsulinemia, driving tumor resistance, and reducing the therapeutic index of PI3K-targeted cancer therapy.[10–14] Furthermore, mice lacking AKT1 and AKT2[15] or a functional mTORC1 complex[16] in their hepatocytes also develop liver damage and HCC. Thus, both excessive and insufficient insulin-PI3K-AKT-mTORC1 signaling have been associated with HCC development.

Although systemic pan-PI3K inhibition is unlikely to achieve a satisfactory therapeutic index in HCC treatment because of on-target side effects,[14] there is a considerable potential for isoform-selective PI3K inhibitors. Previous research indicates PI3Kδ-selective inhibition as a possible therapy for HCC[17] and that PI3Kγ-selective inhibition reduces the effects of obesity on HCC promotion.[18] However, the role of PI3Kα in the progression of HCC remains unresolved.

In this study, we investigated the role of hepatocyte PI3Kα activity in HCC using hepatocyte-specific conditional knockout mice in a model of chemically induced and diet-promoted HCC, which models the HCC promotion in patients with metabolic dysfunction-associated steatotic liver disease (MASLD).

* Corresponding authors. Address: The Wallenberg Laboratory, Institute of Medicine, University of Gothenburg, Bruna Stråket 16, SE-41345, Göteborg, Sweden.
E-mail addresses: barbara.becattini@wlab.gu.se (B. Becattini), giovanni.solinas@wlab.gu.se (G. Solinas).
https://doi.org/10.1016/j.jhepr.2026.101847

Materials and methods

Mice

Experiments were authorized by the Research Animal Ethics Committee of the University of Gothenburg. The mice used were male C57BL/6J mice, maintained at 22 °C with 12-h light:dark cycles at the Experimental BioMedicine (EBM) Facility of the University of Gothenburg. PI3Kα LoxP floxed mice (PI3Kα$^{F/F}$) and mice specifically lacking PI3Kα in their hepatocytes (PI3Kα^{HEP}) were in a 129/SvJ and C57BL/6J mixed background, as previously described.[4]

Two-week-old mice were injected with 25 mg/kg of *N*-nitrosodiethylamine (DEN) and at the age of 6 weeks were fed either a standard rodent chow diet or HFD (60% of calories from fat) until 36 weeks of age. For insulin tolerance test (ITT) and glucose tolerance test (GTT), mice were fasted for 4 h and injected intraperitoneally with 0.75 IU of insulin per kg of body weight or 1 g of glucose per kg of body weight, respectively. Blood glucose concentrations were measured using a glucometer (Contour Next, Ascensia, Basel, Switzerland).

Histology

Livers from PI3Kα$^{F/F}$ and PI3Kα^{HEP} mice were fixed in 10% formalin for 24 h, embedded in paraffin, and cut into 5-μm sections. For Ki67 staining, sections were blocked at room temperature (RT) with 3% H_2O_2 for 20 min and horse serum 1:75 in PBS for 20 min; sections were incubated overnight at 4 °C with Ki67 antibodies in PBS. Tissue sections were then washed and incubated with IgG biotinylated antibodies for 30 min at RT, washed in PBS, and incubated in ABC reagent for 60 min at RT. Tissue sections were then PBS washed, incubated with 3,3′-diaminobenzidine as substrate, and counterstained with hematoxylin. Positive cells were counted in three fields per section. Terminal deoxynucleotidyl transferase dUTP nick-end labeling (TUNEL) was performed according to the manufacturer's instruction (TMR Red, Roche, Basel, Switzerland). For H&E staining, liver sections were deparaffinized in xylene and rehydrated in ethanol at 100%, 95%, and 70% for 2 min each, followed by cold running tap water for 3 min. Sections were then immersed twice in 0.25% ammonia water, rinsed thoroughly with water, stained with eosin (Sigma, Darmstadt, Germany), rinsed with 95% ethanol, dehydrated with three changes of ethanol 95% and 100%, followed by two changes of xylene, and then mounted (Entellan, Merck, Darmstadt, Germany).

Protein sample preparation and immunoblot analysis

Protein samples from nontumor liver and HCC of PI3Kα$^{F/F}$ and PI3Kα^{HEP} mice or primary hepatocytes isolated from PI3Kα$^{F/F}$ and PI3Kα^{HEP} mice were obtained by lysis in 20 mM Tris-HCl, 5% glycerol, 138 mM NaCl, 2.7 mM KCl, 1% NP-40, and 5 mM EDTA with phosphatases and proteases inhibitors. Protein extracts were resolved by SDS-PAGE, transferred onto polyvinylidene fluoride membranes, and incubated with primary antibodies overnight at 4 °C.

Primary hepatocytes

Primary hepatocytes were isolated from PI3Kα$^{F/F}$ and PI3Kα-^{HEP} mice as follows: 12-week-old male mice were anesthetized with isoflurane and sacrificed by bleeding. Liver was perfused with 40 ml of washing solution (Hank's buffered salt solution, GIBCO 14170-112), without magnesium or calcium, and with the addition of 0.5 mM EGTA, and then perfused with a digestion medium comprising DMEM-low glucose (HyClone) with 1% penicillin streptomycin and 15 mM HEPES and 0.8 mg/ml of collagenase type IV. The collagenase digestion was performed at 37 °C for ~10 min, after which Glisson's capsule was torn apart using a pair of sterile forceps to release the collagenase-digested liver into a sterile 10-cm Petri dish containing 10 ml of medium under a cell culture hood. Hepatocytes were dispersed in the medium using a pipette and filtered through a 70-μM cell strainer into a Falcon tube. Cells were collected by centrifugation at RT at 50 *g* for 3 min and washed three times with 20 ml of 50% DMEM high-glucose 50% HAM'S F-12 with 10% FBS, 1% penicillin streptomycin, and 100 nM of dexamethasone. Cells were counted, and viability was evaluated by Trypan Blue exclusion.

For immunoblots experiments, cells were plated in 12-well plates precoated 24 h earlier with type I collagen dissolved in 0.02 N acetic acid. Hepatocytes were seeded at a density of $4x10^5$ viable cells per well in 1 ml of medium. For Ki67 immunostaining, cells were plated in eight-well chamber slides precoated 24 h earlier with type I collagen dissolved in 0.02 N acetic acid. Hepatocytes were seeded at a density of $1x10^5$ viable cells per well in 0.3 ml of medium. For the BrdU proliferation assay, 100 μl of cells at $2x10^5$ cells/ml were seeded into a 96-well culture dish precoated 24 h earlier with type I collagen dissolved in 0.02 N acetic acid. From a 12-week-old mouse, 30–50 million hepatocytes were obtained with a viability of 80–95% (typically ≈90%).

HGF and EGF treatments (immunoblots)

For HGF and EGF dose-response experiments, primary hepatocytes were incubated for 10 min at 37 °C with increasing doses of HGF and EGF (1, 3, and 10 ng/ml) and untreated controls. For chemical mapping experiments, primary hepatocytes were pre-incubated with PI3Kβ, δ, or γ inhibitors, respectively, for 30 min, followed by incubation with either HGF or EGF for 10 min.

HGF and EGF treatments (proliferation assays)

Primary hepatocytes were cultured for 24 h in the absence or presence of a 500 μM mixture of fatty acids at a ratio of 1:2 palmitic acid (PA):oleic acid (OA). After this initial incubation period, the culture medium was replaced with serum-free medium either alone or supplemented with 10 ng/ml o EGF or HGF. Cells were then incubated for an additional 24 h, in either the absence or the continued presence of the fatty acid mixture. At the end of the treatment period, cells were either fixed and subjected to Ki-67 immunostaining or analyzed by BrdU incorporation assay according to the manufacturer's instructions to assess proliferation. For Ki-67 staining, cells were fixed in 4% formaldehyde for 15 min at RT, washed in PBS, permeabilized in 0.3% Triton X-100, washed in PBS, blocked in 2% BSA for 60 min, and incubated overnight with Ki-67 I antibodies (1:600 in blocking buffer). Fixed cells were then incubated with fluorochrome-conjugated II antibodies for 60 min at RT in the dark, washed in PBS, incubated with DAPI

for 5 min at RT to stain nuclei, rinsed in PBS, and mounted. Positive cells were counted in three fields per section.

Real-time PCR RNA profiling

Total RNA was isolated using the guanidinium thiocyanate-phenol-chloroform extraction method from nontumor liver tissue and HCC of PI3Kα$^{F/F}$ and PI3Kα^{HEP} mice injected with DEN and sacrificed acutely 12, 24, and 48 h post injection. Complementary DNA was obtained using a reverse transcription kit (Promega, Wisconsin, USA), and qPCR was performed using a commercial SYBR green mix (SsoAdvanced Universal SYBR Green Supermix, Bio-Rad, California, USA) using specific primers for cyclin D1 and cyclophilin as an internal control.

Statistical analysis

Statistical analysis was performed with GraphPad Prism (GraphPad Software Inc., San Diego, CA, USA). Data are expressed as means and error bars and analyzed using the Wilcoxon-Mann-Whitney *U* test or an unpaired *t* test, and a 2-way ANOVA, followed by the Sidak multiple-comparisons test when two variables were considered; p <0.05 was considered statistically significant.

mRNA sequencing and bioinformatic analysis

Total RNA was isolated using the guanidinium thiocyanate-phenol-chloroform extraction method from nontumor liver tissue and HCC of PI3Kα$^{F/F}$ and PI3Kα^{HEP} mice injected with DEN and sacrificed at 36 weeks of age. mRNA sequencing was performed at Clinical Genomics Gothenburg, SciLifeLab, Göteborg, Sweden. mRNA from six different experimental conditions was sequenced, with seven biological replicates each from a total of 42 sequencing libraries: nontumor liver tissue and HCC from mice fed an HFD; and nontumor liver tissue from mice fed a standard rodent chow diet, for both PI3Kα$^{F/F}$ and PI3Kα^{HEP} mice. Following demultiplexing with bcl2fastq (v2.20; Illumina, Inc., San Diego, CA, USA), read mapping and quantification of the fastq files were performed with the nf-core/rnaseq pipeline (version 3.18.0; nf-core community) with default parameters,[19,20] executed with Nextflow (version 24.10.4; Seqera Labs, Barcelona, Spain).[21] Reads were preprocessed by adapter and quality trimming with Trim Galore! (version 0.6.10; developed at The Babraham Institute, Cambridge, UK), followed by alignment to the GRCm39 mouse reference genome using STAR (version 2.7.11b; developed at Cold Spring Harbor Laboratory, NY, USA).[22] Read quantification was performed with Salmon (version 1.10.3),[23] using the Ensembl release 113 gene annotation.[24] Read quantifications were imported into R (version 4.4.3; R Foundation for Statistical Computing, Vienna, Austra) using tximport (version 1.34.0; Bioconductor Project).[25] Differential expression analysis was performed with DESeq2 (version 1.46.0; Bioconductor Project),[26] comparing gene expression in PI3Kα$^{F/F}$ and PI3Kα^{HEP} mice in each of the different biological conditions.

Gene ontology (GO) over-representation was analyzed using Pathview (version 1.46.0; Bioconductor Project),[27] based on significantly up- or downregulated genes (BH-adjusted Wald test p <0.05). Thematic coloring of the significant GO terms was performed using a manually compiled list of keywords.

Results

PI3Kα ablation in hepatocytes protects mice from HCC

To investigate the role of hepatocyte PI3Kα in the development of HCC, we generated hepatocyte-spcific copnditional KO (PI3Ka^{HEP}) mice by crossing PI3Kα$^{F/F}$ mice with mice expressing the Cre recombinase under the control of the albumin promoter.[4] PI3Kα^{HEP} showed a specific reduction of PI3Kα abundance in their livers, but not in other tissues, without affecting the abundancies of other PI3K isoforms (Fig. S1). PI3Kα^{HEP} and littermate PI3Kα$^{F/F}$ mice were injected at the age of 2 weeks with 25 mg/kg of DEN. By 6 weeks of age, the mice were either kept on a chow diet or placed on an HFD until 36 weeks of age (Fig. 1A). All mice developed HCC by 36 weeks of age (Fig. 1B,C), and obese PI3Kα$^{F/F}$ mice developed significantly more tumors, larger tumors, and an increased total tumor mass compared with lean mice (Fig. 1D–F). However, compared with PI3Kα$^{F/F}$ control mice, PI3Kα^{HEP} mice developed fewer tumors, with a smaller maximal tumor size, and showed a substantial reduction in total tumor mass regardless of the type of diet (Fig. 1D–F). Thus, mice with loss of hepatocyte PI3Kα displayed markedly reduced DEN-induced carcinogenesis, independent of diet type.

Lean PI3Kα^{HEP} mice but not obese PI3Kα^{HEP} mice display reduced HCC proliferation

To investigate the mechanism for the reduced tumor burden in PI3Kα^{HEP} mice, we measured cellular proliferation by Ki67 immunostaining and apoptosis by TUNEL assay of liver sections from the mice described above. The tumor area exhibited a marked increase in cellular proliferation, as indicated by the number of Ki67-positive cells (Fig. 2). However, compared with PI3Kα$^{F/F}$ mice kept on a chow diet, PI3Kα^{HEP} mice showed significantly fewer Ki67-positive cells, specifically in HCC tumors. Hence, loss of PI3Kα reduced tumor cell proliferation (Fig. 2A,B). The number of TUNEL-positive cells was similar in the livers of lean PI3Kα^{HEP} and PI3Kα$^{F/F}$ mice, indicating that loss of PI3Kα does not affect hepatocyte apoptosis (Fig. 2C,D). For PI3Kα^{HEP} mice and PI3Kα$^{F/F}$ mice kept on an HFD, we observed a similar number of Ki67-positive cells in normal liver and HCC areas, as well as a comparable number of TUNEL-positive cells. Overall, the protective effects of PI3Kα ablation on HCC could be explained, at least partially, by reduced HCC proliferation in lean mice. However, HCC proliferation was not affected by PI3Kα deletion in obese mice, indicating that an additional mechanism is responsible for the reduced tumor mass in obese mice.

DEN-injected PI3Kα^{HEP} mice develop mild glucose intolerance and hyperinsulinemia on an HFD

When maintained on a chow diet, PI3Kα^{HEP} mice and PI3Kα$^{F/F}$ mice showed similar growth curves, glucose tolerance, and insulin tolerance (Fig. S2). However, when placed on an obesogenic HFD, PI3Kα^{HEP} mice showed reduced weight gain compared with PI3Kα$^{F/F}$ control mice. By 25 weeks of age, PI3Kα^{HEP} mice showed glucose intolerance and hyperinsulinemia, which were normalized mainly by 36 weeks of age (Fig. S3). Overall, PI3Kα^{HEP} mice showed a normal metabolic phenotype on a chow diet and transient glucose intolerance and hyperinsulinemia on an HFD.

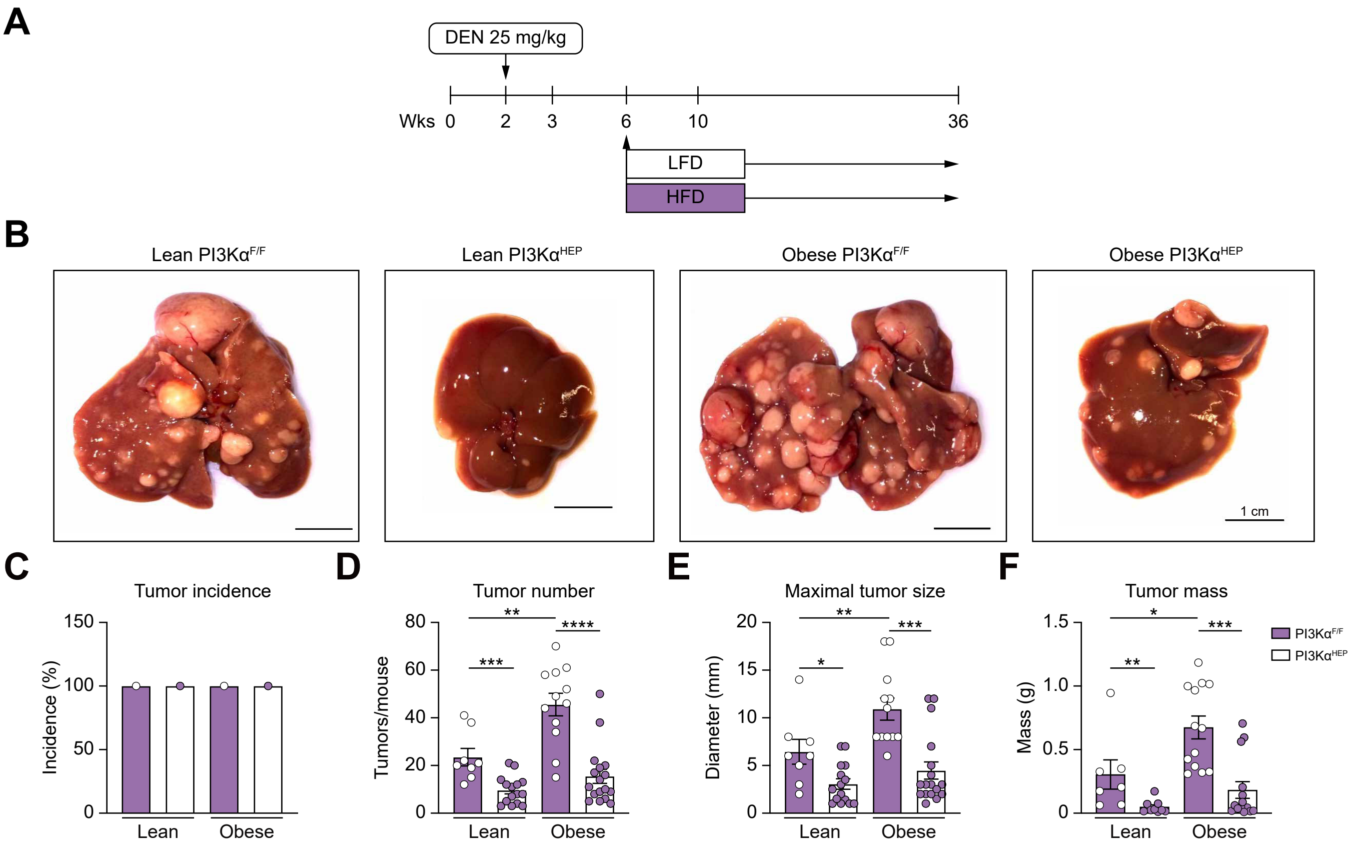

Fig. 1. Loss of hepatocyte PI3Kα protects mice from hepatocellular carcinoma (HCC). (A) Experimental time course. (B) Representative images of livers from PI3Kα$^{F/F}$ and PI3Kα^{HEP} mice injected with DEN and kept either on a chow diet or on obesogenic HFD as described in (A). (C) Tumor incidence in the mice from (B). (D) Number of liver tumors and (E) maximal tumor size of the mice in (A). (F) Total mass of liver tumors per mouse. n = 8–17 mice. Data are mean ± s.e.m. Statistical analysis was performed using Mann-Whitney. One asterisk (*) for p ≤0.05, two asterisks (**) for p ≤0.01, three asterisks (***) for p ≤0.001. DEN, *N*-nitrosodiethylamine; HFD, high-fat diet; PI3K, phosphoinositide 3-kinase; PI3Kα$^{F/F}$, PI3Kα LoxP floxed; PI3Kα^{HEP}, mice specifically lacking PI3Kα in their hepatocytes.

Circulating liver enzymes and lipids are reduced in DEN-injected PI3Kα^{HEP} mice

Compared with control mice, DEN-injected PI3Kα^{HEP} mice showed reduced serum abundance of alanine aminotransferase (ALT) when kept on an HFD, and albumin was slightly but significantly reduced on chow and HFD (Fig. S4). Total serum cholesterol was also reduced, and serum triglycerides were reduced specifically in the chow diet (Fig. S4). Overall, these results are consistent with reduced liver damage and altered lipid metabolism.

PI3Kα^{HEP} mice display altered tumor gene expression despite normal AKT phosphorylation

Analysis of AKT and ERK phosphorylation showed similar abundances in normal livers and tumors of PI3Kα^{HEP} mice and PI3Kα$^{F/F}$ mice, indicating that PI3Kα ablation in hepatocytes is not sufficient to affect AKT and ERK signaling (Fig. 3A–D). However, mRNA sequencing analysis of gene expression revealed a distinct signature in tumors from PI3Kα^{HEP} mice fed an HFD compared with PI3Kα$^{F/F}$ mice. Tumors from PI3Kα^{HEP} mice kept on HFD showed 890 differentially expressed genes (DEGs), of which 532 were upregulated and 358 downregulated (Fig. S5). GO term enrichment analysis identified several significant terms related to immune response among the upregulated genes (Fig. 3E; Fig. S6). Tumors from PI3Kα^{HEP} mice fed an HFD showed a more inflammatory phenotype compared with those from PI3Kα$^{F/F}$ mice. Among the downregulated genes, we found significant GO terms related to different cellular processes, including cell–cell junction maintenance, Golgi vesicle transport, cell–cell junction organization, and protein secretion (Fig. 3E). Notably, we found that different GO terms related to lipid metabolism were also significant, including triglyceride metabolic processes, fatty acid metabolic processes, triglyceride biosynthetic processes, and positive regulation of triglyceride metabolic processes (Fig. 3E,F; Fig. S6). Analysis of tumor-specific DEGs did not indicate a specific pathway (Fig. S14). Overall, our data indicate that loss of PI3Kα in HCC and normal liver does not affect AKT phosphorylation in these tissues, but does lead to clear changes in HCC gene expression profiles, suggesting more inflamed tumors and reduced lipid metabolism.

PI3Kα^{HEP} mice display altered gene expression in the normal liver, indicating reduced lipid transport and metabolism

PI3Kα^{HEP} and PI3Kα$^{F/F}$ mice showed similar AKT phosphorylation in normal liver tissue, indicating that PI3Kα ablation in hepatocytes is not sufficient to affect AKT signaling (Fig. 3A–D). mRNA sequencing analysis of gene expression from the normal livers of PI3Kα^{HEP} mice and PI3Kα$^{F/F}$ mice, both kept on a chow

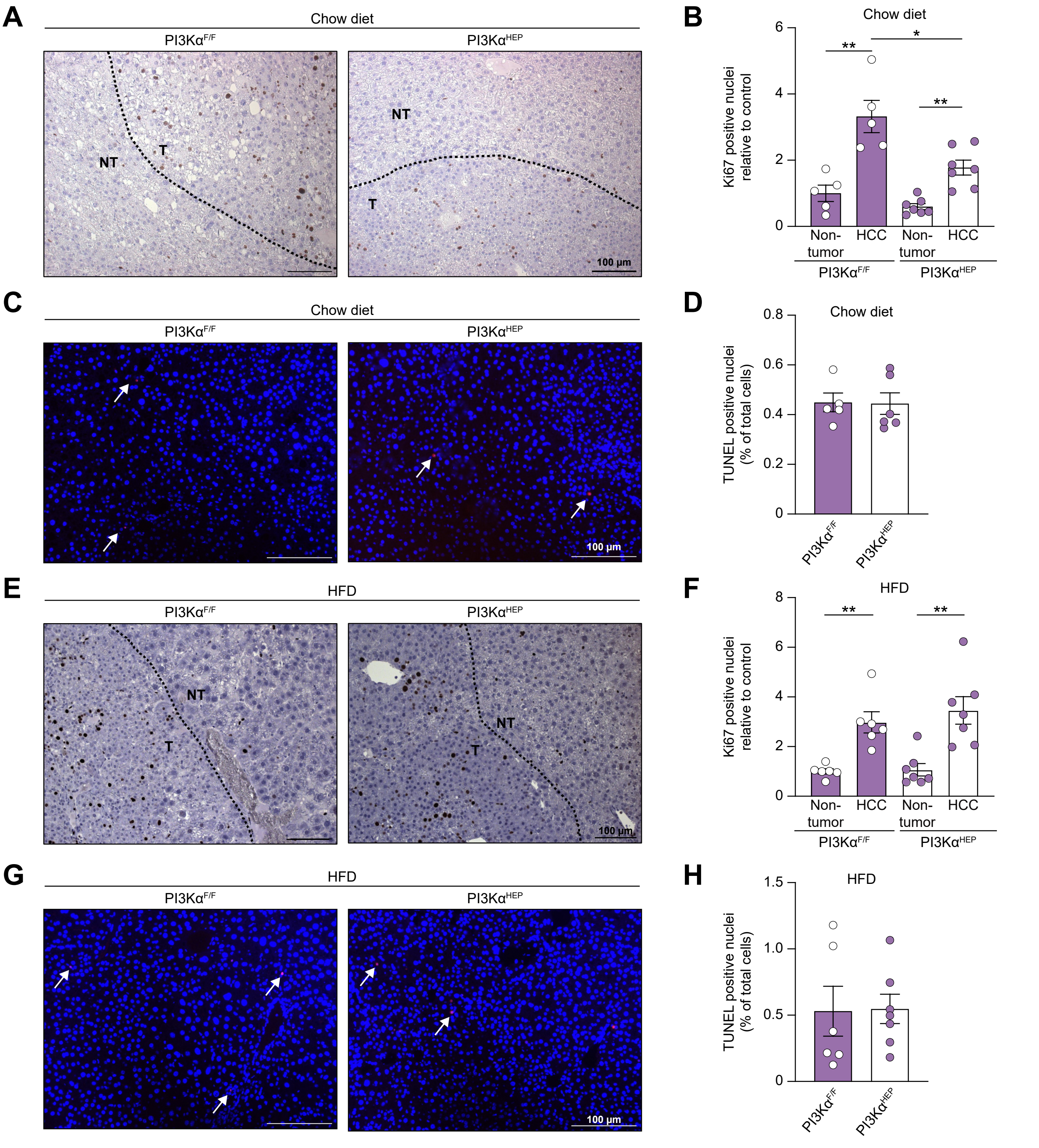

Fig. 2. Effects of PI3Kα deletion on cell proliferation and apoptosis. (A) Ki-67 immunostaining of liver sections from PI3Kα$^{F/F}$ and PI3Kα^{HEP} mice kept on a chow diet described in Fig. 1. (B) Quantification of the number of Ki-67-positive cells from (A) in normal liver and HCC. (C) TUNEL assay of liver section from mice in (B). (D) Quantification of the TUNEL-positive nuclei from (C). (E) Ki-67 immunostaining of liver section from PI3Kα$^{F/F}$ and PI3Kα^{HEP} mice fed an obesogenic HFD. (F) Quantification of Ki-67-positive nuclei from (E). (G) TUNEL assay of the liver section from PI3Kα$^{F/F}$ and PI3Kα^{HEP} mice fed an obesogenic HFD. (H) Quantification of the TUNEL-positive nuclei from (G). n = 5–7 mice. Data are mean ± s.e.m. Statistical analysis was performed using Mann-Whitney. One asterisk (*) for $p \leq 0.05$, two asterisks (**) for $p \leq 0.01$. HFD, high-fat diet; PI3K, phosphoinositide 3-kinase; PI3Kα$^{F/F}$, PI3Kα LoxP floxed; PI3Kα^{HEP}, mice specifically lacking PI3Kα in their hepatocytes; TUNEL, terminal deoxynucleotidyl transferase dUTP nick-end labeling.

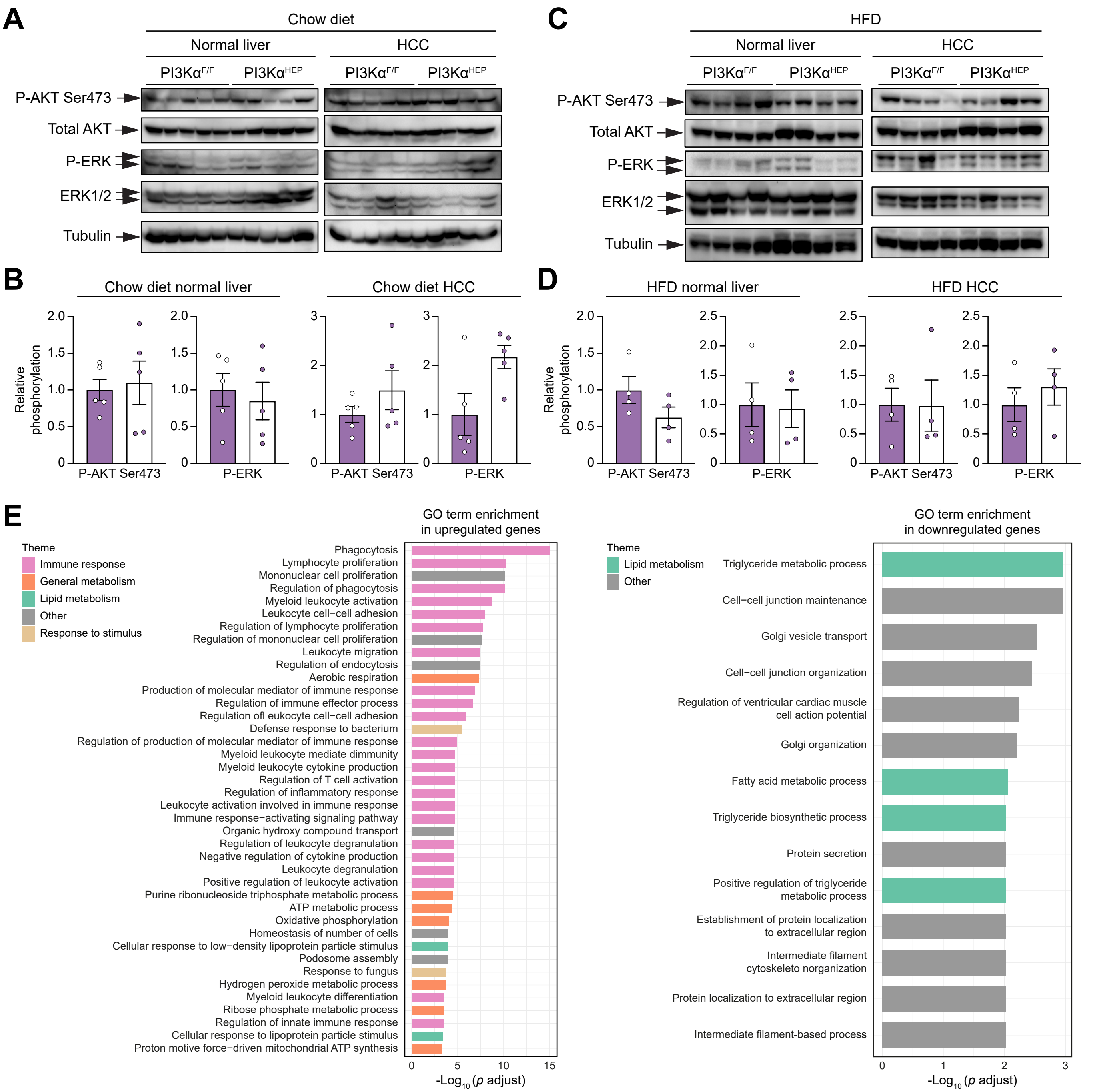

Fig. 3. Loss of PI3Kα alters tumor gene expression but not AKT phosphorylation. (A) Immunoblot analysis of AKT and ERK phosphorylation in normal liver or HCC from PI3KαF/F and PI3KαHEP mice fed a chow diet. (B) Quantification of the blots in (A). (C) Immunoblot analysis of AKT and ERK phosphorylation in normal liver or HCC from PI3KαF/F and PI3KαHEP mice fed an HFD. (D) Quantification of the blots in (C). (E) GO term enrichment in upregulated and downregulated genes in HCC from PI3KαHEP mice vs HCC from PI3KαF/F mice fed an HFD. (F) Heatmaps of DEGs in the ontology categories: regulation_of_lipid_metabolic_process; lipid_transport; lipid_catabolic_process; and triglyceride_metabolic_process. n = 5 mice per group for A-B, n = 4 for mice per group for C-D and n = 7 mice per group for E-F. A-D: Data are mean ± s.e.m. Statistical analysis was performed using Mann-Whitney. E: For GO term enrichment analysis, significance was determined using hypergeometric test p-values (BH-adjusted). The top 40 terms with *p*-adj <0.01 are shown. DEG, differentially expressed gene; GO, Gene Ontology; HCC, hepatocellular carcinoma; HFD, high-fat diet; PI3K, phosphoinositide 3-kinase; PI3KαF/F, PI3Kα LoxP floxed; PI3KαHEP, mice specifically lacking PI3Kα in their hepatocytes.

diet, revealed 1,418 DEGs, with 749 upregulated and 669 downregulated (Figs S7 and S11). GO term enrichment analysis indicated that, among the upregulated genes, cytoplasmic translation was the most significant GO term (Fig. 4A; Fig. S8). GO terms related to oxidative respiration, aerobic respiration, proton-motive force-driven ATP synthesis, mitochondrial respirasome assembly, and ribose phosphate biosynthetic process were also significantly enriched in upregulated genes. Among downregulated genes, there was a clear pattern of GO terms related to lipid transport and metabolism: lipid transport,

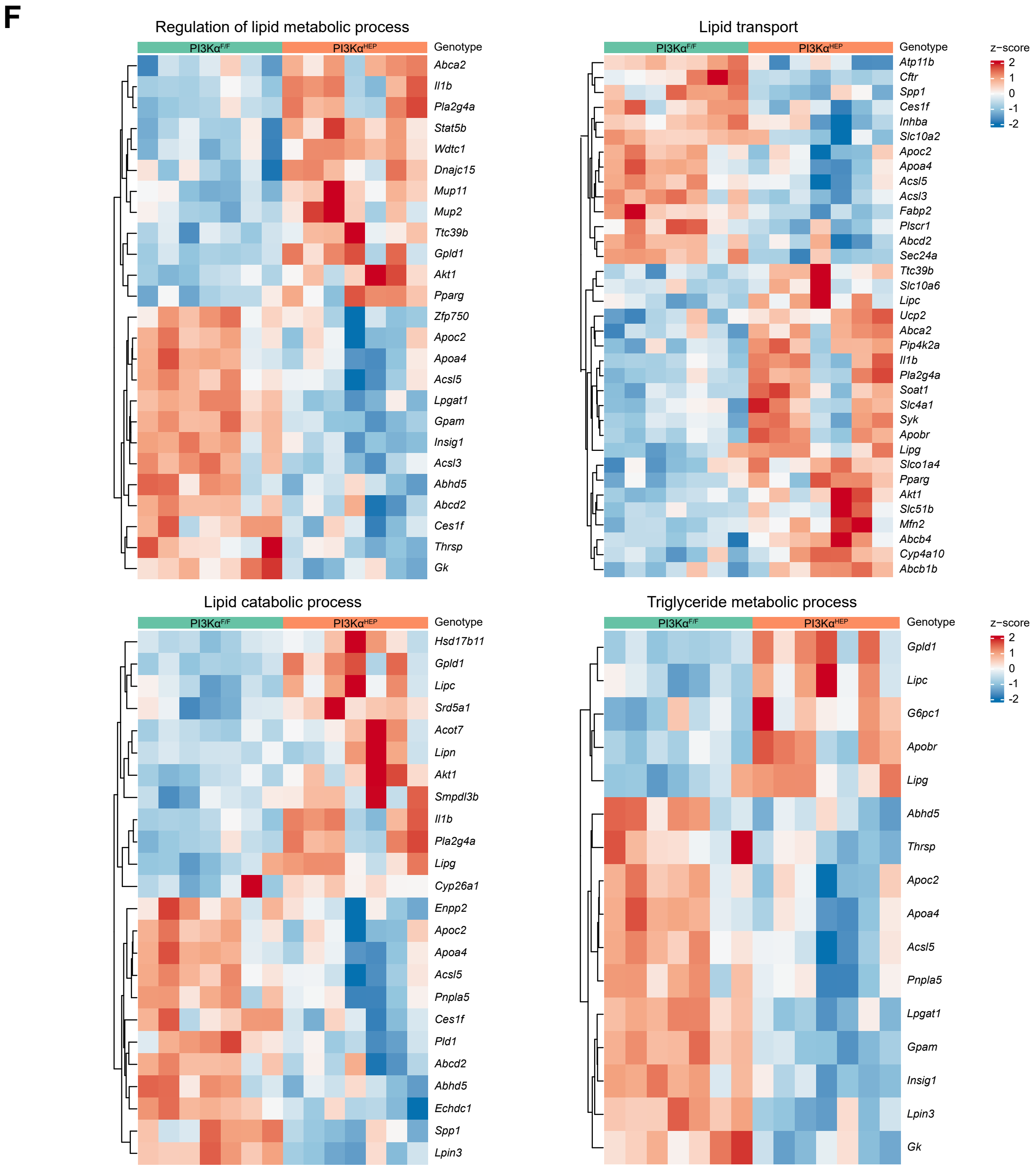

Fig. 3. (Continued).

glycerolipid metabolic processes, regulation of lipid metabolic processes, regulation of lipid localization, fatty acid biosynthetic processes, regulation of lipid storage, lipid homeostasis, intestinal cholesterol absorption, glycerolipid catabolic process, and lipid digestion (Fig. 4A,C; Figs S8, S11, and S12). H&E staining of the liver section from PI3Kα^{HEP} mice and PI3Kα$^{F/F}$ mice showed that hepatocyte expression of PI3Kα was necessary for lipid droplet formation (Fig. 4B).

mRNA sequencing analysis from the normal livers of PI3Kα^{HEP} and PI3Kα$^{F/F}$ mice, both kept on an HFD, revealed 1,814 DEGs, with 902 upregulated and 912 downregulated. When placed on an HFD, mRNA sequencing of normal liver tissues from PI3Kα^{HEP} and PI3Kα$^{F/F}$ mice showed increased proinflammatory gene expression in livers from PI3Kα^{HEP} mice (Fig. 4D,F; Figs S9–S13). Among the genes downregulated in PI3Kα^{HEP} mice, there were several GO terms relative to lipid transport and metabolism: fatty acid metabolic processes, lipid catabolic processes, lipid transport, regulation of lipid catabolic processes, regulation of lipid localization, lipid homeostasis, regulation of triglyceride metabolic processes, lipid

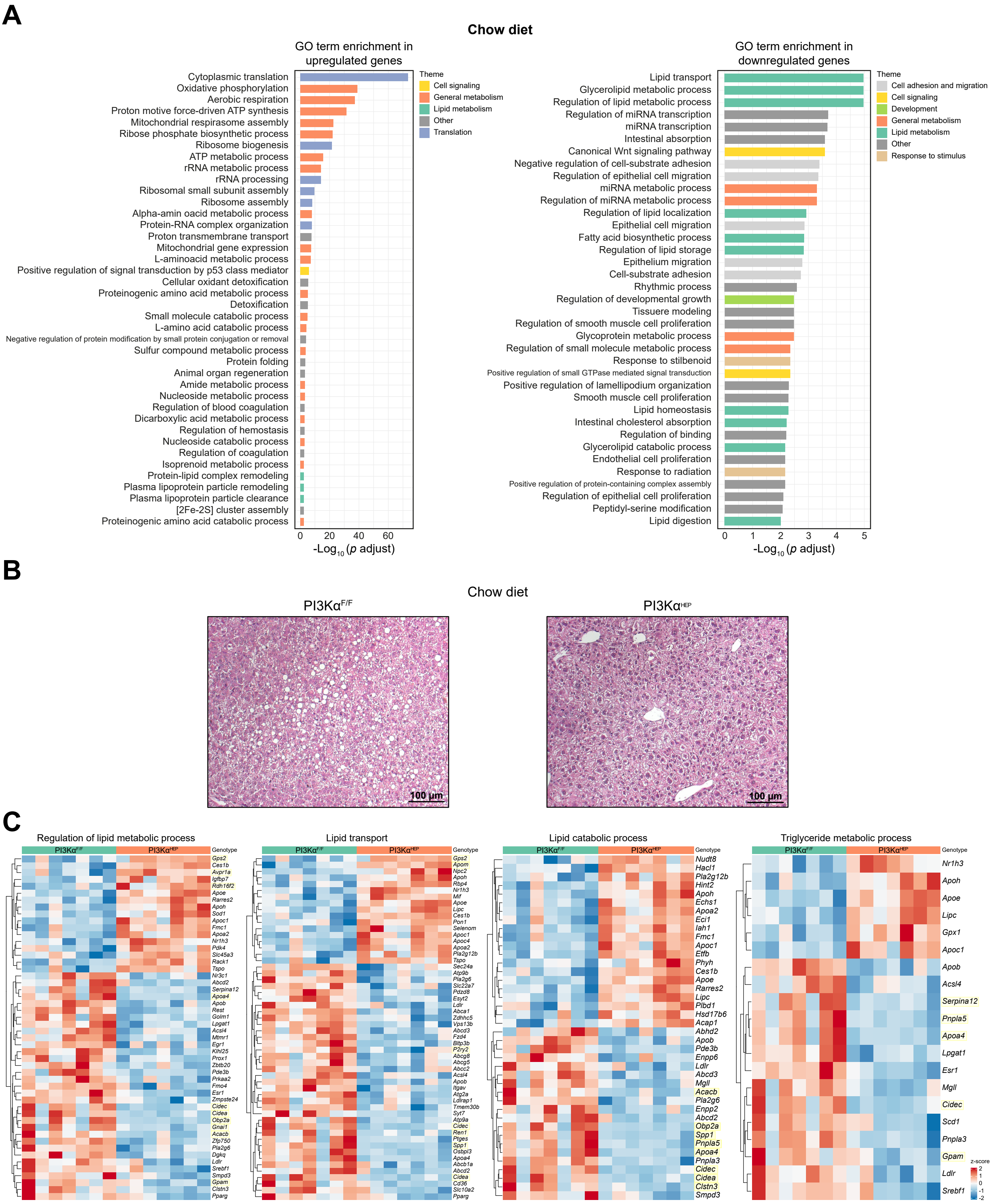

Fig. 4. Loss of PI3Kα alters the expression of genes in lipid metabolism in normal liver. (A) GO term enrichment in upregulated and downregulated genes in normal liver of PI3Kα^{HEP} mice *vs.* PI3Kα$^{F/F}$ mice fed a chow diet. (B) H&E staining of sections from livers in (A). (C) Heatmaps of DEGs in the GO categories: regulation_o-f_lipid_metabolic_process; lipid_transport; lipid_catabolic_process; triglyceride_metabolic_process from A. (D) GO term enrichment in upregulated and downregulated genes in normal liver from PI3Kα^{HEP} mice *vs.* from PI3Kα$^{F/F}$ mice fed an HFD. (E) H&E staining of sections from the livers in (D). (F) Heatmaps of DEGs in the ontology

droplet organization, and regulation of lipid transport (Fig. 4D,F; Figs S9–S13). H&E staining of liver sections from PI3Kα^{HEP} mice and PI3Kα$^{F/F}$ mice kept on an HFD demonstrated an essential role for hepatocyte PI3Kα in lipid droplet formation (Fig. 4E).

From individual analyses, we found similar GO terms in mice maintained on a chow diet or an HFD. By analyzing genes regulated similarly in the chow diet and HFD, normal liver tissue and HCC, we were able to verify which GO terms were common to PI3Kα^{HEP} mice *vs.* PI3Kα$^{F/F}$ (Figs S11 and S12). In line with the individual analyses, immune response dominated GO terms for upregulated genes, and lipid metabolism was among the GO terms for downregulated genes.

By contrast, there were substantial differences in the regulated genes in the two diets (Fig. S13). Genes upregulated in HFD, relative to the regulation in the chow diet, were involved in development. Lipid metabolism and more general metabolism terms were among the genes that were 'more' downregulated in HFD compared with chow diet-fed mice (Fig. S13).

Together, these results suggest that PI3Kα has a more general role in lipid droplet formation rather than controlling a linear signal to a specific transcription factor.

PI3Kα^{HEP} mice display reduced hepatocyte proliferation during carcinogen initiation

Although PI3Kα^{HEP} mice developed fewer tumors and smaller tumors compared with PI3Kα$^{F/F}$ mice, regardless of the diet, we found reduced HCC proliferation only in PI3Kα^{HEP} mice fed a chow diet but not in mice fed an HFD (Figs 1 and 2). Hence, we hypothesized that PI3Kα is required for the acute hepatocyte proliferation induced by the carcinogen DEN during tumor initiation.

Compared with PBS control mice, at 12 h after DEN injection, PI3Kα^{HEP} mice injected with DEN showed a significant reduction in the number of Ki67-positive hepatocytes and reduced liver expression of cyclin D1 (Fig. 5A–C). PI3Kα$^{F/F}$ mice also showed reduced cyclin D1 but not reduced proliferating hepatocytes, as assessed by Ki67 staining (Fig. 5A–C). DEN induced the number of apoptotic cells to a similar extent in PI3Kα$^{F/F}$ mice and PI3Kα^{HEP} mice (Fig. 1A,D). As expected, at 24 h post-DEN injection, we observed in PI3Kα$^{F/F}$ mice a significant increase in the number of Ki67-positive hepatocytes and increased liver cyclin D1 expression compared with PBS control-injected mice (Fig. 5E–G).

However, this induction of hepatocyte proliferation was blunted in livers from PI3Kα^{HEP} (Fig. 5E–G). No significant difference was detected between genotypes in the number of apoptotic cells 24 h post-DEN injection. By 48 h post-DEN injection, there were no more differences in the number of Ki67-positive cells, Cyclin D1 expression, or TUNEL-positive hepatocytes. Overall, we concluded that hepatocyte PI3Kα was necessary for the acute induction of hepatocyte proliferation following DEN injection.

PI3Kα is dispensable for AKT phosphorylation induced by HGF and EGF

We measured AKT and ERK phosphorylation in the livers of PI3Kα$^{F/F}$ mice and PI3Kα^{HEP} mice 24 h after DEN injection, when we observed a difference in hepatocyte proliferation (Fig. 5). The results showed a similar abundance of AKT serine 473 phosphorylation and ERK phosphorylation in livers from PI3Kα^{HEP} and PI3Kα$^{F/F}$ mice (Fig. 6A,B), indicating that PI3Kα controls hepatocyte proliferation independently of AKT phosphorylation. Acute hepatocyte proliferation in response to liver damage (*e.g.* after DEN exposure during tumor initiation) depends on HGF and EGF, the two most potent hepatocytic growth factors, which act as complete mitogens.[28] Therefore, we also measured AKT and ERK phosphorylation in primary hepatocytes from PI3Kα$^{F/F}$ mice and PI3Kα^{HEP} mice exposed to increasing doses of HGF and EGF. The results demonstrated that PI3Kα was dispensable for HGF and EGF signaling in primary mouse hepatocytes and that loss of PI3Kα was compensated for by another PI3K isoform (Fig. 6C–F).

AKT phosphorylation induced by HGF and EGF depends on redundant PI3Kα and PI3Kβ activities

To identify the PI3K isoform compensating for the loss of PI3Kα in HGF and EGF signaling in hepatocytes, we performed a pharmacological mapping using a panel of isoform-selective PI3K inhibitors. The lowest doses of the PI3Kβ-selective inhibitor GSK2636771 reduced HGF-induced AKT phosphorylation by >50% in primary hepatocytes from PI3Kα$^{F/F}$ mice, and this was reduced to basal levels by 400 nM of GSK2636771 in primary hepatocytes from PI3Kα^{HEP} mice (Fig. 7A,B). These results indicate that HGF-induced AKT phosphorylation depends on PI3Kα and PI3Kβ. The PI3Kδ-selective inhibitor PIK294 and the PI3Kγ-selective inhibitor IPI549 inhibited AKT phosphorylation with similar potency in primary hepatocytes from PI3Kα$^{F/F}$ mice and PI3Kα^{HEP} mice. However, AKT phosphorylation was effectively inhibited only at higher doses of PIK294 and IPI549, where there was no isoform selectivity (Fig. 8E–F). An almost identical pattern to that seen for HGF-induced AKT phosphorylation was also observed for EGF-induced AKT phosphorylation in primary hepatocytes from PI3Kα$^{F/F}$ mice and PI3Kα^{HEP} mice treated with PI3K isoform-selective inhibitors (Fig. 7G–L).

Overall, our results indicate that HGF- and EGF-induced AKT phosphorylation in primary mouse hepatocytes depends on the redundant activities of PI3Kα and PI3Kβ.

Hepatocyte proliferation induced by HGF and EGF depends on PI3Kα

Motivated by our *in vivo* findings of the role of PI3Kα in hepatocyte proliferation in response to the DEN injection (Fig. 5A,B,E,F), we investigated the role of PI3Kα in the effects of HGF and EGF on the proliferation of cultured primary

categories: regulation_of_lipid_metabolic_process; lipid_transport; lipid_catabolic_process; triglyceride_metabolic_process from D. n = 7 mice per group. A, D: For GO term enrichment analysis, significance was determined using hypergeometric test *p*-values (BH-adjusted). The top 40 terms with p <0.01 are shown. DEG, differentially expressed gene; GO, Gene Ontology; HCC, hepatocellular carcinoma; HFD, high-fat diet; PI3K, phosphoinositide 3-kinase; PI3Kα$^{F/F}$, PI3Kα LoxP floxed; PI3Kα^{HEP}, mice specifically lacking PI3Kα in their hepatocytes.

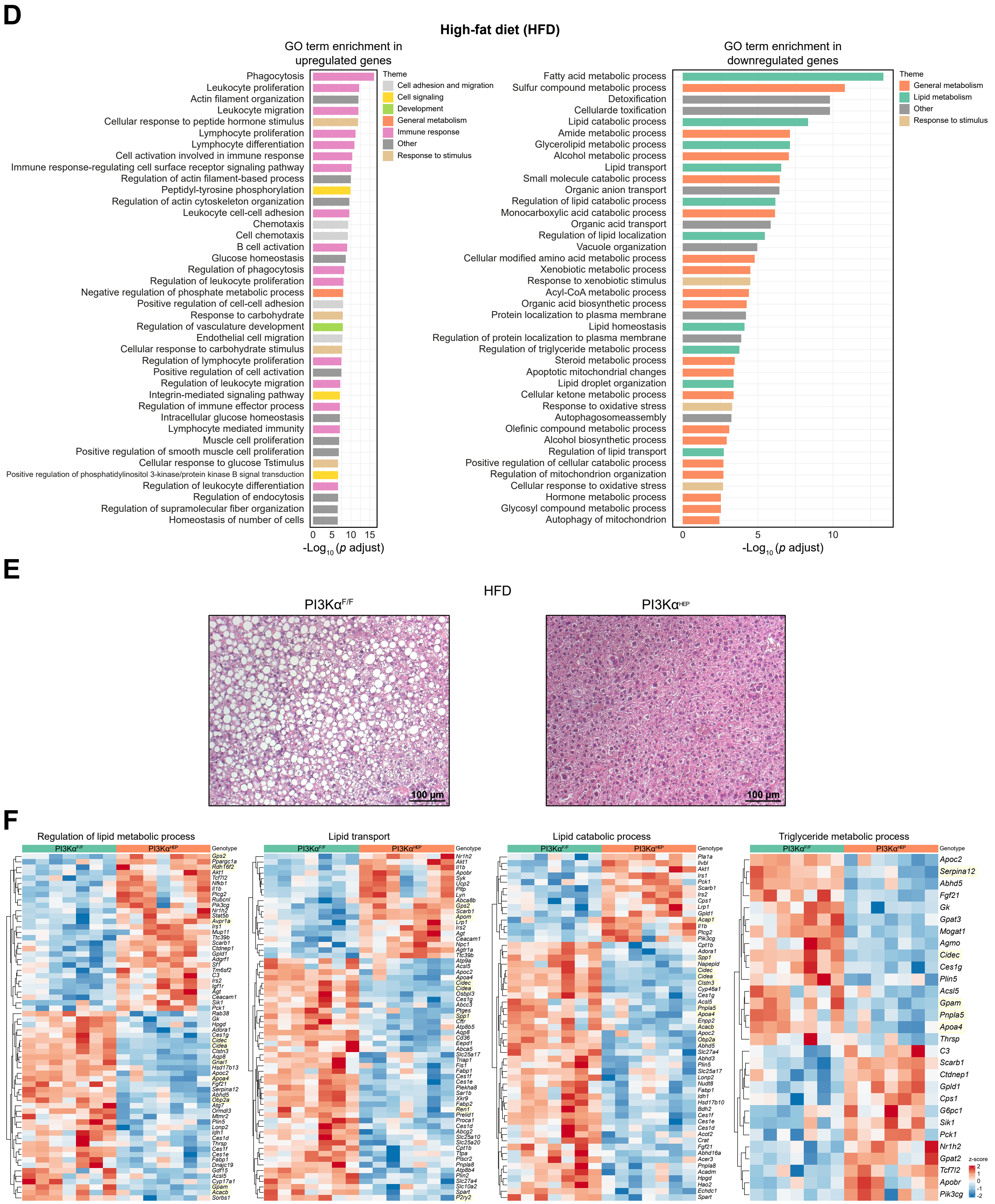

Fig. 4. (Continued).

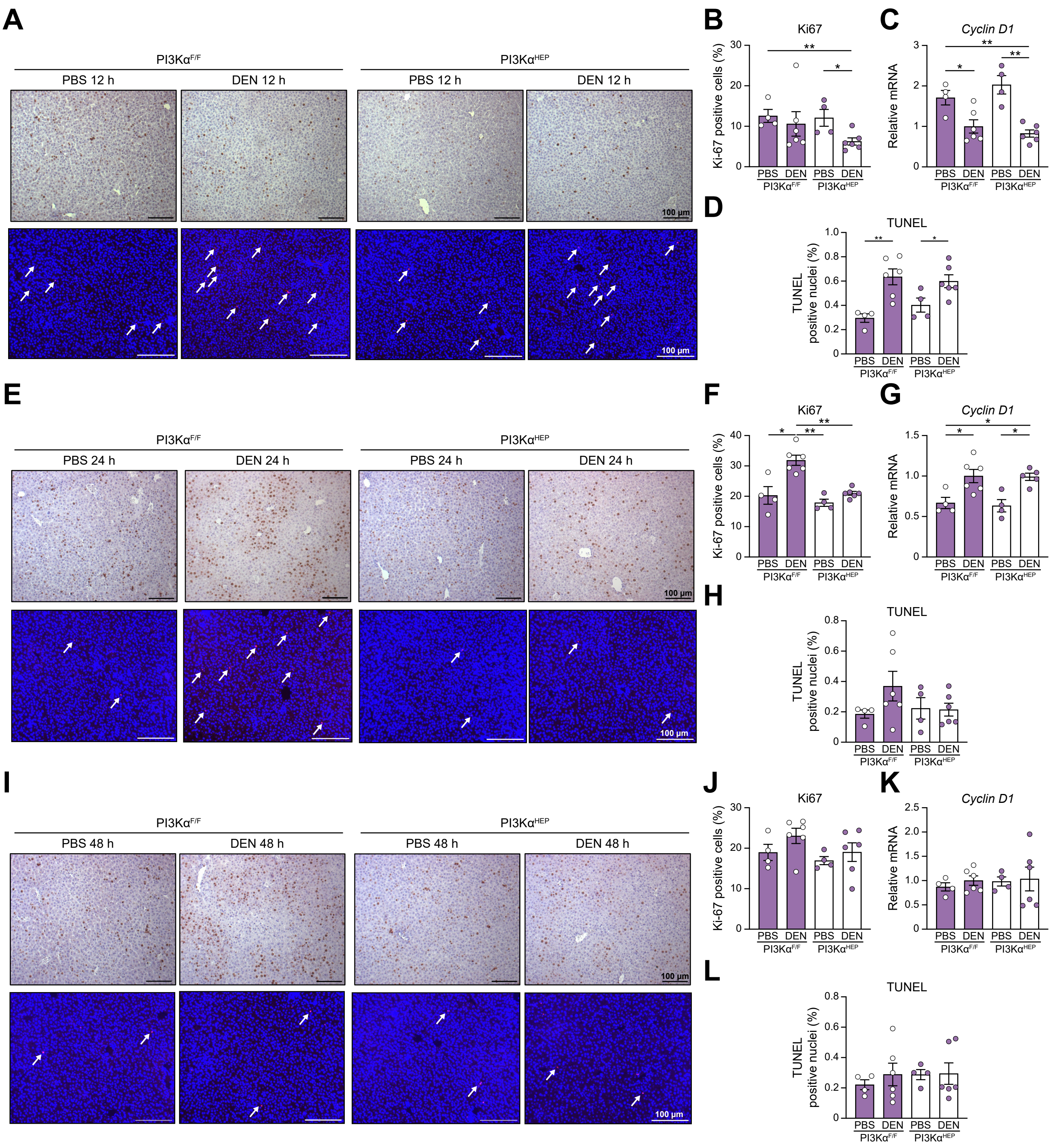

Fig. 5. PI3Kα ablation reduces DEN-induced proliferation. (A,E,I) Ki-67 (top) and TUNEL assay (bottom) of 2-week-old PI3Kα$^{F/F}$ and PI3Kα^{HEP} mice (A) 12 h, (E) 24 h, and (I) 48 h after DEN injection. (B) Quantification of Ki-67-positive nuclei in (A). (C) *Cyclin D1* mRNA abundance in the livers of (A). (D) Quantification of TUNEL-positive nuclei from (A). (F) Quantification of the Ki67-positive nuclei in (E). (G) *Cyclin D1* mRNA abundance in livers in (E). (H) Quantification of TUNEL-positive nuclei from (E). (J) Quantification of Ki-67-positive nuclei from (I). (K) Cyclin D1 mRNA abundance in the livers of (I). (L) Quantification of TUNEL-positive nuclei from (I). n = 4–6 mice per group. Data are mean±s.e.m. Statistical analysis was performed using Mann-Whitney. One asterisk (*) for $p \leq 0.05$, two asterisks (**) for $p \leq 0.01$. DEN, *N*-nitrosodiethylamine; PI3K, phosphoinositide 3-kinase; PI3Kα$^{F/F}$, PI3Kα LoxP floxed; PI3Kα^{HEP}, mice specifically lacking PI3Kα in their hepatocytes; TUNEL, terminal deoxynucleotidyl transferase dUTP nick-end labeling.

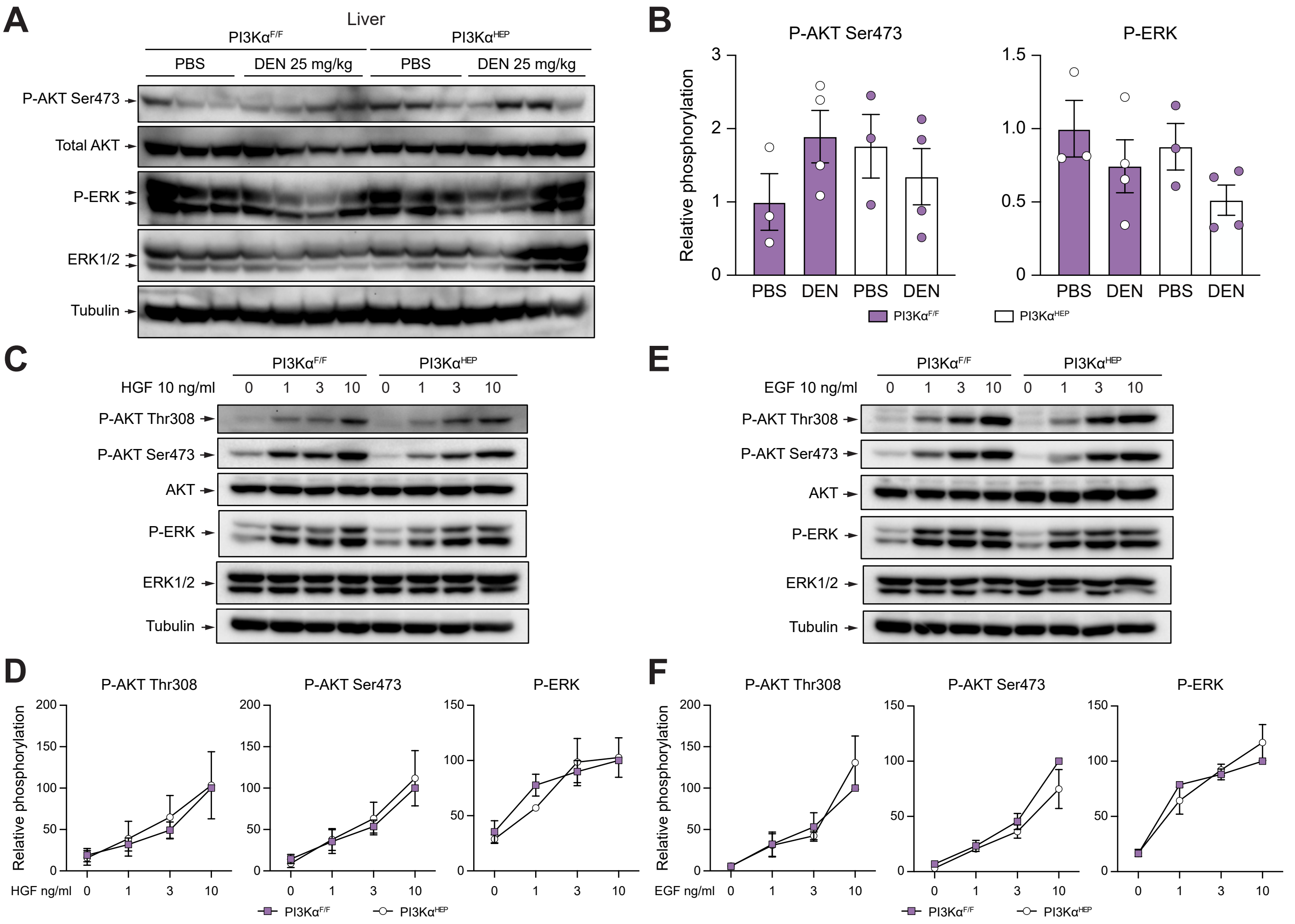

Fig. 6. PI3Kα is dispensable for HGF and EGF signaling. (A) Immunoblot analysis of AKT and ERK phosphorylation in whole liver of mice 24 h after injection with DEN. (B) Quantification of blots in (A). (C,E) Immunoblot analysis of AKT and ERK phosphorylation in primary mouse hepatocytes exposed to increasing doses of (C) HGF and (E) EGF. (D) Quantification of immunoblots in (C). (F) Quantification of immunoblots in (E). n = 3–4 mice per genotype. Statistical analysis was performed using unpaired *t*-test for A-B and two-way ANOVA for C–F. DEN, *N*-nitrosodiethylamine; EGF, epidermal growth factor; HGF, hepatocyte growth factor; PI3K, phosphoinositide 3-kinase.

hepatocytes from PI3Kα$^{F/F}$ mice and PI3Kα^{HEP} mice. Primary hepatocytes were either serum starved for 24 h or incubated with either EGF or HGF in the absence of serum. To investigate the effects of lipids on hepatocyte proliferation, cells were also incubated with a 500 μM mixture of fatty acids (1:2 PA and OA) starting 24 h before serum starvation, for a total of 48 h. Compared with primary hepatocytes from PI3Kα$^{F/F}$ mice, Ki67 immunostaining showed fewer Ki67-positive nuclei in primary hepatocytes from PI3Kα^{HEP} mice treated with HGF or EGF (Fig. 8A–C), indicating a role of PI3Kα in hepatocyte proliferation induced by HGF and EGF. This difference was independent of the presence of lipids in the medium. This result was confirmed by the BrdU incorporation assay, which directly measures cell proliferation. Indeed, hepatocytes from PI3Kα^{HEP} mice showed reduced BrdU incorporation after exposure to either EGF or HGF, with the exception of hepatocytes treated with EGF in the presence of PA/OA, where the difference did not achieve statistical significance.

Altogether, our results indicate that the effects of EGF and HGF on the proliferation of cultured primary hepatocytes depend on PI3Kα, despite normal AKT phosphorylation.

Discussion

Several lines of evidence implicate hepatocyte PI3K-AKT signaling in the development of HCC.[5–9] However, pan-inhibition of PI3K activity is unlikely to achieve a satisfactory therapeutic index because it is associated with liver damage and severe hyperglycemia and hyperinsulinemia.[12–14] Importantly, complete blockage of AKT-mTORC1 signaling in mice causes chronic liver damage and HCC.[15,16] Therefore, both excessive and insufficient PI3K signaling were associated with HCC. In this study, we showed that mice lacking PI3Kα in their hepatocytes displayed markedly reduced carcinogen-induced HCC, regardless of diet (chow or HFD). This finding is highly relevant to patients with fatty liver disease, given that the incidence of MASLD-related HCC is increasing. The reduced tumor burden in mice lacking PI3Kα in their hepatocytes could be partially explained by reduced HCC proliferation, and partly by reduced hepatocyte proliferation induced 24 h after carcinogen administration. Indeed, damage-induced hepatocyte proliferation, such as that acutely induced by DEN, has been linked to tumor initiation in

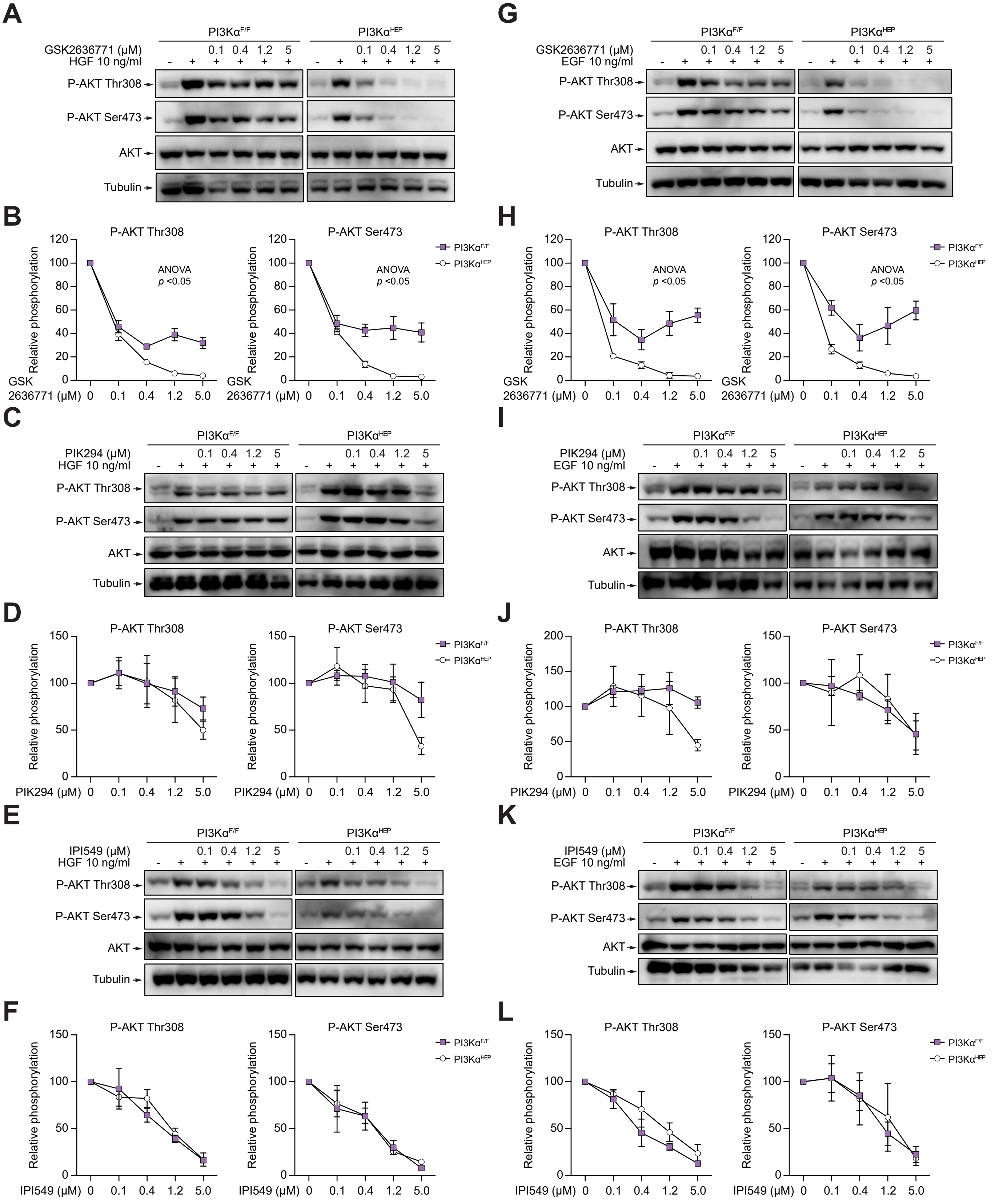

Fig. 7. HGF and EGF signaling depend on PI3Kα and PI3Kβ. (A,C,E) Immunoblot analysis of AKT phosphorylation in primary mouse hepatocytes exposed to HGF and increasing doses of (A) the PI3Kβ-selective inhibitor GSK2636771, (C) the PI3Kδ-selective inhibitor PIK2954, and (E) the PI3Kγ-selective inhibitor IPI549 (B) Quantification of the blots in (A). (D) Quantification of the blots in (C). (F) Quantification of the blots in (E). (G,I,K) Immunoblot analysis of AKT phosphorylation in primary mouse hepatocytes exposed to EGF and increasing doses of (G) the PI3Kβ-selective inhibitor GSK2636771, (I) the PI3Kδ-selective inhibitor PIK2954, and (K) the PI3Kγ-selective inhibitor IPI549. (H) Quantification of the blots in (G). (J) Quantification of the immunoblots in (I). ((L) Quantifications of the immunoblots in (K). n = 3 mice per genotype and treatment. Data are mean ± s.e.m. Statistical analysis was performed using Mann-Whitney. One asterisk (*) for $p \leq 0.05$, two asterisks (**) for $p \leq 0.01$. EGF, epidermal growth factor; HGF, hepatocyte growth factor; PI3K, phosphoinositide 3-kinase.

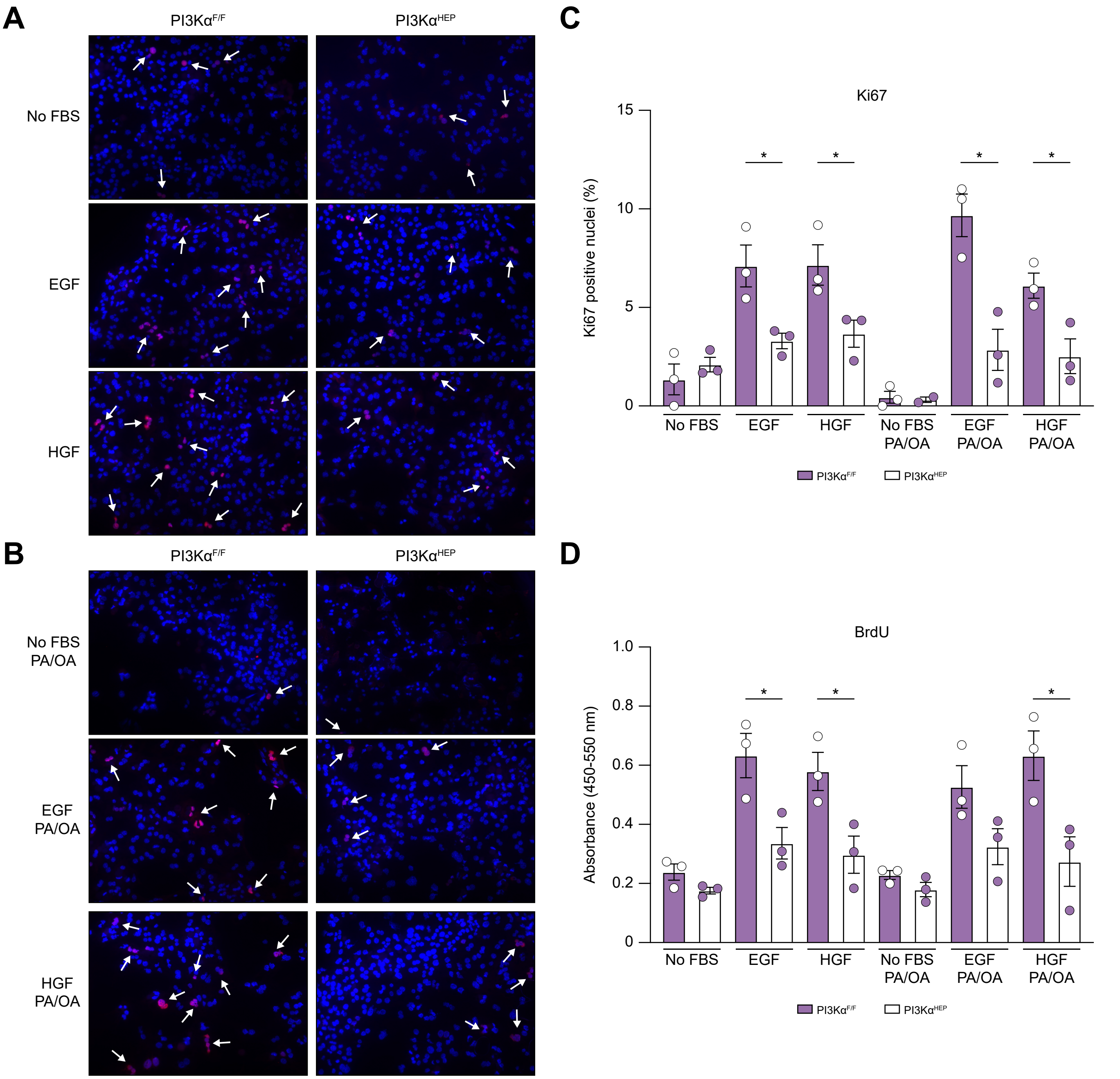

Fig. 8. PI3Kα ablation reduces EGF and HGF-induced proliferation in primary murine hepatocytes. (A) Ki-67 immunostaining of primary mouse hepatocytes isolated from PI3Kα$^{F/F}$ and PI3Kα^{HEP} mice cultured in FBS-free medium, or medium supplemented with EGF and HGF. (B) As in (A) but in the presence of a 1:2 mixture of PA and OA. (C) Quantification of Ki-67-positive nuclei shown in (A,B). (D) BrdU cell proliferation assay of primary mouse hepatocytes from PI3Kα$^{F/F}$ and PI3Kα^{HEP} mice cultured in FBS-free medium, or medium supplemented with EGF and HGF in the absence or presence of a 1:2 mixture of PA and OA. n = 3 mice per genotype and treatment. Data are mean±s.e.m. Statistical analysis was performed using Mann-Whitney. One asterisk (*) for $p \leq 0.05$, two asterisks (**) for $p \leq 0.01$. EGF, epidermal growth factor; HGF, hepatocyte growth factor; OA, oleic acid; PA, palmitic acid; PI3K, phosphoinositide 3-kinase; PI3Kα$^{F/F}$, PI3Kα LoxP floxed; PI3Kα^{HEP}, mice specifically lacking PI3Kα in their hepatocytes.

a variety of experimental conditions.[28] To our surprise, loss of PI3Kα did not alter AKT phosphorylation in either normal liver or HCC of mice fed either a chow diet or an HFD. Loss of PI3Kα also did not alter AKT phosphorylation in mice 24 h after DEN injection. PI3Kα was also dispensable for AKT phosphorylation induced by HGF and EGF, the two most potent hepatocytic growth factors driving hepatocyte proliferation following liver damage (*e.g.* the damage induced by DEN).[28] Indeed, we found that AKT phosphorylation induced by HGF and EGF in hepatocytes depended on the redundant activities of PI3Kα and PI3Kβ. We have previously reported that insulin-induced hepatocyte AKT phosphorylation also depends on the redundant activities of PI3Kα and PI3Kβ.[4] Thus, it is unlikely that AKT phosphorylation in PI3Kα^{HEP} mice is affected by the feeding state. Nonetheless, loss of PI3Kα in hepatocytes had significant effects on gene expression in

HCC and normal liver. Compared with HCC from PI3Kα$^{F/F}$ mice, HCC from PI3Kα^{HEP} mice kept on HFD showed 890 DEGs, with a gene expression signature indicating increased proinflammatory gene expression and decreased expression of several genes involved in lipid metabolism and lipid transport. In the normal liver of the same PI3Kα$^{F/F}$ and PI3Kα^{HEP} mice, we found 1,814 DEGs in HFD and 1,418 DEGs in the chow diet. For both diets, we also observed decreased expression of several genes implicated in lipid metabolism and lipid transport in PI3Kα^{HEP} mice compared with PI3Kα$^{F/F}$ mice. Although this gene expression signature was observed in mice fed either a chow diet or an HFD, there was a substantial difference in the specifically DEGs, all of which pertained to the same GO terms. This pattern of gene expression, together with the fact that AKT phosphorylation was similar in livers from PI3Kα^{HEP} and PI3Kα$^{F/F}$ mice, indicates a general role of PI3Kα in the control of lipid metabolism, which is unlikely to be linked to a linear pathway to a specific transcription factor. This pattern of gene expression was associated with the absence of lipid droplet formation in the livers of PI3Kα^{HEP} mice.

The lack of lipid droplet formation in PI3Kα^{HEP} mice has been previously described by others.[29,30] However, here we dissociate this phenotype from elevated blood glucose and show that it occurs in the presence of normal AKT phosphorylation. This finding suggests a specific role for PI3Kα activity in lipid metabolism, which is independent of AKT and is not compensated for by PI3Kβ or other PI3K isoforms. The fact that we observed reduced HCC proliferation in PI3Kα^{HEP} mice fed a chow diet but not in mice fed an HFD suggests that PI3Kα is important in producing lipids that promote HCC proliferation, which might be compensated for by dietary lipids. Indeed, this was the case for triglycerides. It is also possible that this metabolic phenotype is responsible for the reduction in the proliferation induced acutely by the carcinogen DEN. Indeed, this reduced proliferation also occurred despite normal AKT phosphorylation in livers lacking PI3Kα, and PI3Kα was dispensable for AKT phosphorylation induced by the most potent hepatocytic growth factors, HGF and EGF. Nonetheless, primary hepatocytes lacking PI3Kα showed reduced proliferation in response to HGF and EGF, which is consistent with a role for PI3Kα in hepatocyte proliferation independent of AKT phosphorylation.

In conclusion, we found that the loss of PI3Kα in the hepatocyte protected mice from DEN-induced HCC occurs despite normal AKT phosphorylation in both HCC and the normal liver, the latter after both chronic and acute DEN exposure. AKT phosphorylation induced by HGF and EGF in primary hepatocytes was mediated by redundant PI3Kα and PI3Kβ activities. Nonetheless, loss of hepatocyte PI3Kα activity was associated with reduced hepatocyte proliferation induced by HGF and EGF, significant changes in gene expression, particularly in lipid metabolism and lipid transport, and PI3Kα activity was essential for the formation of lipid droplets. We hypothesize that this metabolic phenotype is implicated in the reduced proliferation observed in HCC of PI3Kα^{HEP} mice and in hepatocytes from these mice 24 h after exposure to the carcinogen DEN. However, this remains to be demonstrated.

Our study has some limitations. We investigated only one HCC model and it will be important to investigate other HCC models with varying degrees of liver damage. Furthermore, we did not measure phosphoinositide levels and, therefore, cannot exclude reduced PIP3 production in PI3Kα^{HEP} mice. Finally, we measured AKT phosphorylation *in vivo* only in the morning and, therefore, we cannot exclude that PI3Kα^{HEP} mice display reduced AKT phosphorylation at other specific circadian times. However, circadian AKT phosphorylation in hepatocytes depends on feeding and insulin, and we previously showed that insulin signaling in hepatocytes depends on redundant PI3Kα and PI3Kβ activities.[4]

Although this has not yet been formally demonstrated in humans, our results indicate that hepatocyte PI3Kα is a potentially actionable therapeutic target for HCC in lean patients and patients with obesity.

Affiliations

[1]*The Wallenberg Laboratory, Institute of Medicine, University of Gothenburg, Gothenburg, Sweden;* [2]*The Bioinformatics and Data Centre, University of Gothenburg, Gothenburg, Sweden;* [3]*Department of Oncology, Institute of Clinical Sciences, Sahlgrenska Academy, University of Gothenburg, Gothenburg, Sweden;* [4]*Sahlgrenska Center for Cancer Research, Sahlgrenska Academy, University of Gothenburg, Gothenburg, Sweden*

Abbreviations

ALT, alanine aminotransferase; DEG, differentially expressed gene; DEN, *N*-nitrosodiethylamine; EGF, epidermal growth factor; GO, Gene Ontology; GTT, glucose tolerance test; HCC, hepatocellular carcinoma; HFD, high-fat diet; HGF, hepatocyte growth factor; ITT, insulin tolerance test; MASLD, metabolic dysfunction-associated steatotic liver disease; OA, oleic acid; PA, palmitic acid; PI3K, phosphoinositide 3-kinase; PI3Kα$^{F/F}$, PI3Kα LoxP floxed; PI3Kα^{HEP}, mice specifically lacking PI3Kα in their hepatocytes; PTEN, phosphatidylinositol (3,4,5)-trisphosphate phosphatase; RT, room temperature; TUNEL, Terminal deoxynucleotidyl transferase dUTP nick-end labeling.

Financial support

This study was supported by funding from the Swedish Research Council (2022-01033), The Cancerfonden (232829 Pj), and the Diabetesfonden (DIA2022-753).

Conflicts of interest

The authors declare no conflicts of interest.

Please refer to the accompanying ICMJE disclosure forms for further details.

Authors' contributions

Performed experiments, analyzed data, and contributed to manuscript writing: BB. Performed experiments and analyzed data: CS, ACG. Analyzed data and contributed to manuscript writing: BE. Analyzed data: TP, KH. Procured funding, designed experiments, analyzed data, and wrote the manuscript: GS.

Data availability statement

Original immunoblots images are available at Mendeley data (https://doi.org/10.17632/g6fndymznk.1). mRNA sequencing data are available on GEO (GSE302836). The code for downstream analysis of transcriptomics is available on Figshare (https://doi.org/10.6084/m9.figshare.29931302) or GitHub (https://github.com/Bart-Edelbroek/PI3Ka_HCC).

Acknowledgements

None provided.

Supplementary data

Supplementary data to this article can be found online at https://doi.org/10.1016/j.jhepr.2026.101847.

References

Author names in bold designate shared co-first authorship

[1] Global Burden of Disease Cancer Collaboration, Fitzmaurice C, Akinyemiju TF, Al Lami FH, et al. Global, regional, and national cancer incidence, mortality, years of life lost, years lived with disability, and disability-adjusted life-years for 29 cancer groups,1990 to 2016: a systematic analysis for the Global Burden of Disease Study. JAMA Oncol 2018;4:1553–1568.

[2] **Priestley P**, **Baber J**, Lolkema MP, et al. Pan-cancer whole-genome analyses of metastatic solid tumours. Nature 2019;575:210–216.

[3] Bidkhori G, Benfeitas R, Klevstig M, et al. Metabolic network-based stratification of hepatocellular carcinoma reveals three distinct tumor subtypes. Proc Natl Acad Sci U S A 2018;115:E11874–E11883.

[4] **Molinaro A**, **Becattini B**, Mazzoli A, et al. Insulin-driven PI3K-AKT signaling in the hepatocyte is mediated by redundant PI3Kalpha and PI3Kbeta Activities and is promoted by RAS. Cell Metab 2019;29:1400–1409.

[5] Dombrowski F, Mathieu C, Evert M. Hepatocellular neoplasms induced by low-number pancreatic islet transplants in autoimmune diabetic BB/Pfd rats. Cancer Res 2006;66:1833–1843.

[6] Molinaro A, Becattini B, Solinas G. Insulin signaling and glucose metabolism in different hepatoma cell lines deviate from hepatocyte physiology toward a convergent aberrant phenotype. Sci Rep 2020;10:12031.

[7] Scheiter A, Evert K, Reibenspies L, et al. RASSF1A independence and early galectin-1 upregulation in PIK3CA-induced hepatocarcinogenesis: new therapeutic venues. Mol Oncol 2022;16:1091–1118.

[8] Calvisi DF, Wang C, Ho C, et al. Increased lipogenesis, induced by AKT-mTORC1-RPS6 signaling, promotes development of human hepatocellular carcinoma. Gastroenterology 2011;140:1071–1083.

[9] Horie Y, Suzuki A, Kataoka E, et al. Hepatocyte-specific Pten deficiency results in steatohepatitis and hepatocellular carcinomas. J Clin Invest 2004;113:1774–1783.

[10] Solinas G, Becattini B. An adipoincretin effect links adipostasis with insulin secretion. Trends Endocrinol Metab 2024;35:466–477.

[11] Becattini B, Molinaro A, Henricsson M, et al. Adipocyte PI3K links adipostasis with baseline insulin secretion at fasting through an adipoincretin effect. Cell Rep 2024;43:114132.

[12] Solinas G, Becattini B. PI3K and AKT at the interface of signaling and metabolism. Curr Top Microbiol Immunol 2022;436:311–336.

[13] Hopkins BD, Pauli C, Du X, et al. Suppression of insulin feedback enhances the efficacy of PI3K inhibitors. Nature 2018;560:499–503.

[14] Janku F, Yap TA, Meric-Bernstam F. Targeting the PI3K pathway in cancer: are we making headway? Nat Rev Clin Oncol 2018;15:273–291.

[15] **Wang Q**, **Yu WN**, Chen X, et al. Spontaneous hepatocellular carcinoma after the combined deletion of Akt isoforms. Cancer Cell 2016;29:523–535.

[16] Umemura A, Park EJ, Taniguchi K, et al. Liver damage, inflammation, and enhanced tumorigenesis after persistent mTORC1 inhibition. Cell Metab 2014;20:133–144.

[17] Ko E, Seo HW, Jung ES, et al. PI3Kdelta is a therapeutic target in hepatocellular carcinoma. Hepatology 2018;68:2285–2300.

[18] Becattini B, Breasson L, Sardi C, et al. PI3Kgamma promotes obesity-associated hepatocellular carcinoma by regulating metabolism and inflammation. JHEP Rep 2021;3:100359.

[19] Ewels PA, Peltzer A, Fillinger S, et al. The nf-core framework for community-curated bioinformatics pipelines. Nat Biotechnol 2020;38:276–278.

[20] Patel H, Ewels P, Manning J, et al. nf-core/rnaseq: nf-core/rnaseq v3.18.0 - Lithium Lynx. https://doi.org/10.5281/zenodo.14537300. Accessed 7 April 2026.

[21] Di Tommaso P, Chatzou M, Floden E, et al. Nextflow enables reproducible computational workflows. Nat Biotechnol 2017;35:316–319.

[22] Dobin A, Davis CA, Schlesinger F, et al. STAR: ultrafast universal RNA-seq aligner. Bioinformatics 2013;29:15–21.

[23] Patro R, Duggal G, Love M, et al. Salmon provides fast and bias-aware quantification of transcript expression. Nat Methods 2017;14:417–419.

[24] Dyer SC, Austine-Orimoloye O, Azov AG, et al. Ensembl 2025. Nucleic Acids Res 2025;53:D948–D957.

[25] Soneson C, Love MI, Robinson MD. Differential analyses for RNA-seq: transcript-level estimates improve gene-level inferences. F1000Research 2016;4:1521.

[26] Love MI, Huber W, Anders S. Moderated estimation of fold change and dispersion for RNA-seq data with DESeq2. Genome Biol 2014;15:550.

[27] Luo W, Brouwer C. Pathview: an R/Bioconductor package for pathway-based data integration and visualization. Bioinformatics 2013;29:1830–1831.

[28] Michalopoulos GK, Bhushan B. Liver regeneration: biological and pathological mechanisms and implications. Nat Rev Gastroenterol Hepatol 2021;18:40–55.

[29] Regnier M, Polizzi A, Fougeray T, et al. Liver gene expression and its rewiring in hepatic steatosis are controlled by PI3Kalpha-dependent hepatocyte signaling. Plos Biol 2025;23:e3003112.

[30] Chattopadhyay M, Selinger ES, Ballou LM, et al. Ablation of PI3K p110-alpha prevents high-fat diet-induced liver steatosis. Diabetes 2011;60:1483–1492.

Keywords: Phosphoinositide 3 kinase; Metabolic dysfunction-associated steatotic liver disease; Obesity; Growth factors; Metabolic syndrome; Cancer.

Received 11 September 2025; received in revised form 18 March 2026; accepted 27 March 2026; Available online 15 April 2026

Journal of Hepatology, Volume *8*

Supplemental information

Loss of hepatocyte PI3Kα reduces hepatocellular carcinoma and hepatocyte proliferation in association with altered lipid metabolism

Barbara Becattini, Claudia Sardi, Bart Edelbroek, Amit Chand Gupta, Toshima Parris, Khalil Helou, and Giovanni Solinas

Loss of hepatocyte PI3Kα protects mice from hepatocellular carcinoma in association with altered lipid metabolism gene expression

Barbara Becattini, Claudia Sardi, Bart Edelbroek, Amit Chand Gupta, Toshima Parris, Khalil Helou, Giovanni Solinas

Table of contents

Supplementary figures 2

Fig. S1

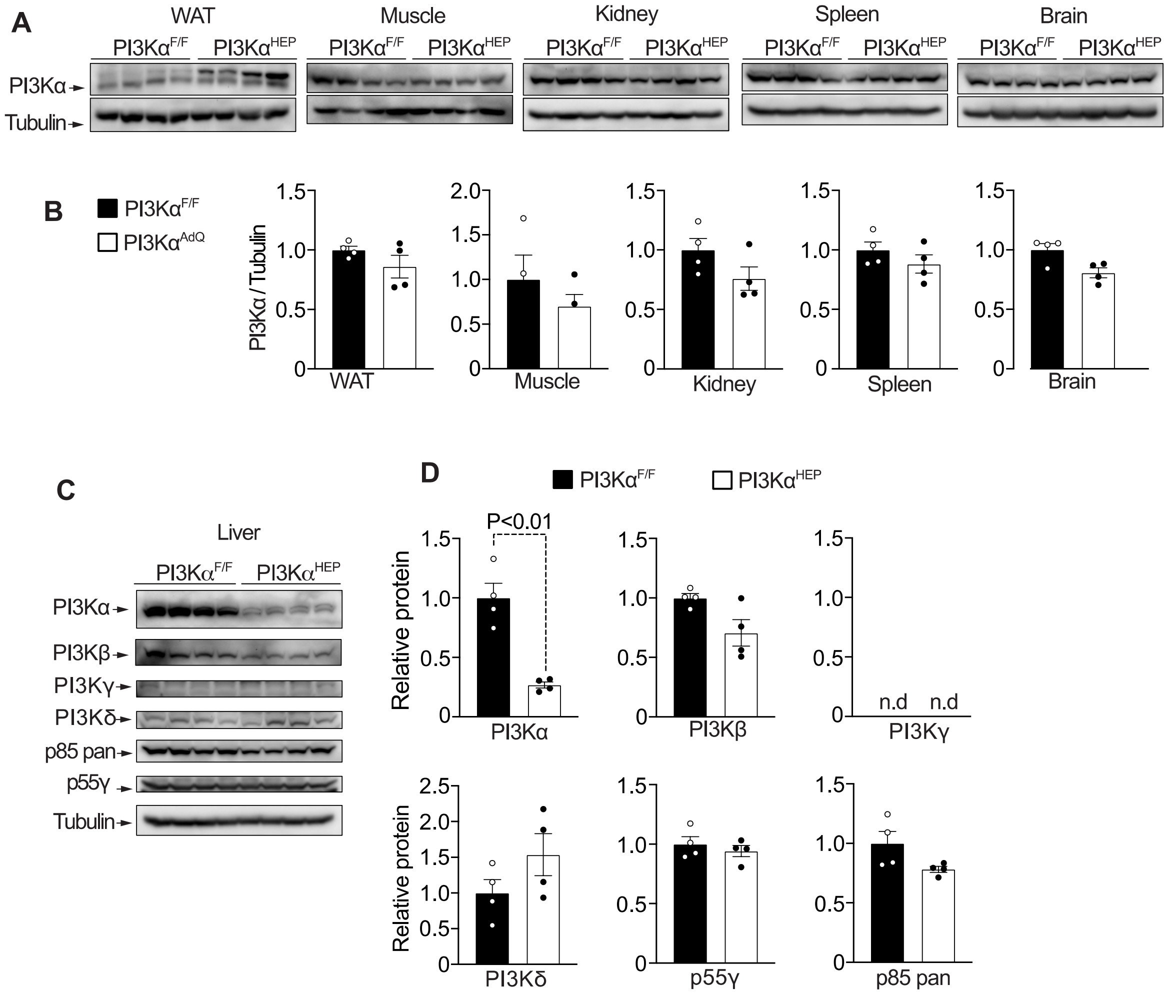

Fig. S1. (Related to Fig. 1). Characterization of PI3Kα deletion in PI3Kα^{Hep} mice.

(A) Immunoblots analysis of PI3Kα abundances in white adipose tissue (WAT) muscle, kidney, spleen, and brain. (B) Quantifications of the blots in A. (C) Immunoblot analysis of different class-1 PI3K in the liver of PI3Kα^{Hep} mice and PI3Kα$^{F/F}$ mice. (D) Quantifications of the blots in C. Data are represented as means, and error bars indicate standard errors. Statistical analysis was performed using Mann-Whitney.

n=4 mice per group.

Fig. S2

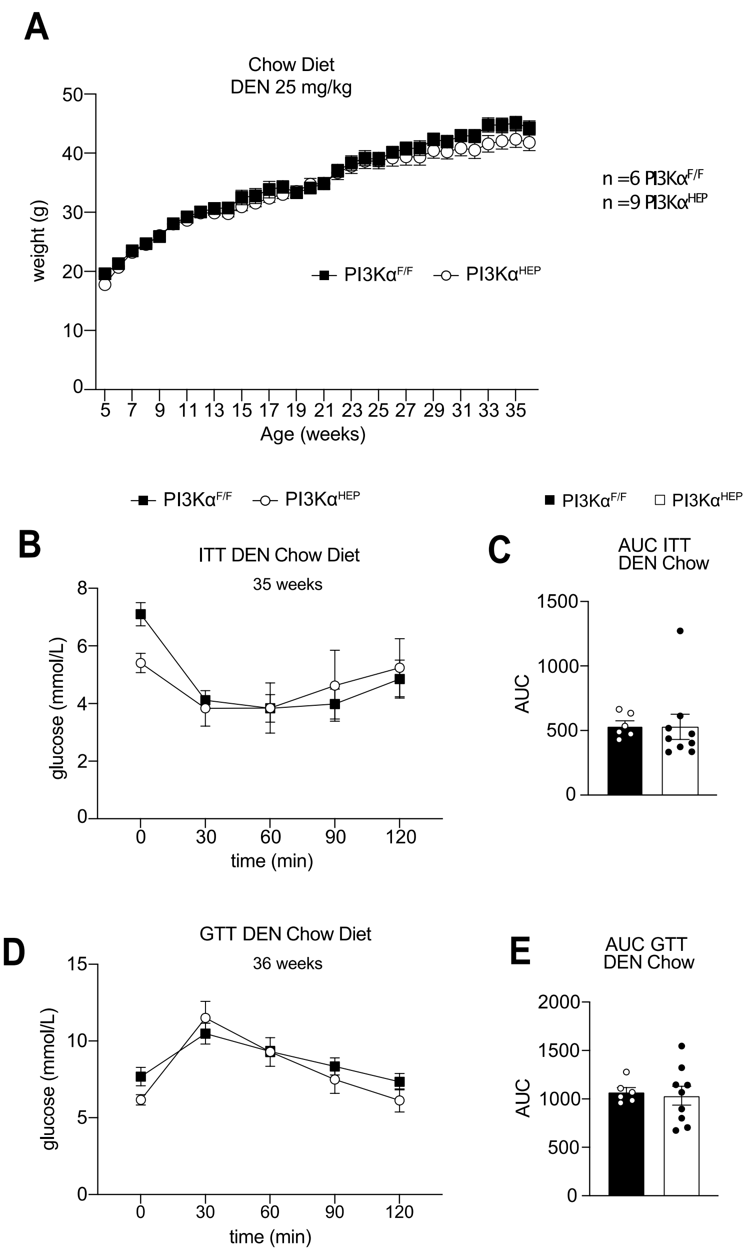

Fig. S2. (Related to Fig. 1). Loss of hepatocyte PI3Kα has a negligible impact on growth and glucose homeostasis in DEN-injected mice fed a chow diet.

(A) Growth curve of PI3Kα$^{F/F}$ and PI3Kα^{HEP} mice kept on a chow diet. (B) Insulin tolerance test (ITT) of the mice from A at the age of 35 weeks. (C) Area under the curves of the ITT in B. (D) Glucose tolerance test (GTT) of the mice in A at the age of 36 weeks. (E) Area under the curve of the GTT in D. n=6-9 mice

Data are represented as means, and error bars indicate standard errors. Statistical analysis was performed using Mann-Whitney for C and E, and two-way ANOVA for A,B, D.

Fig. S3

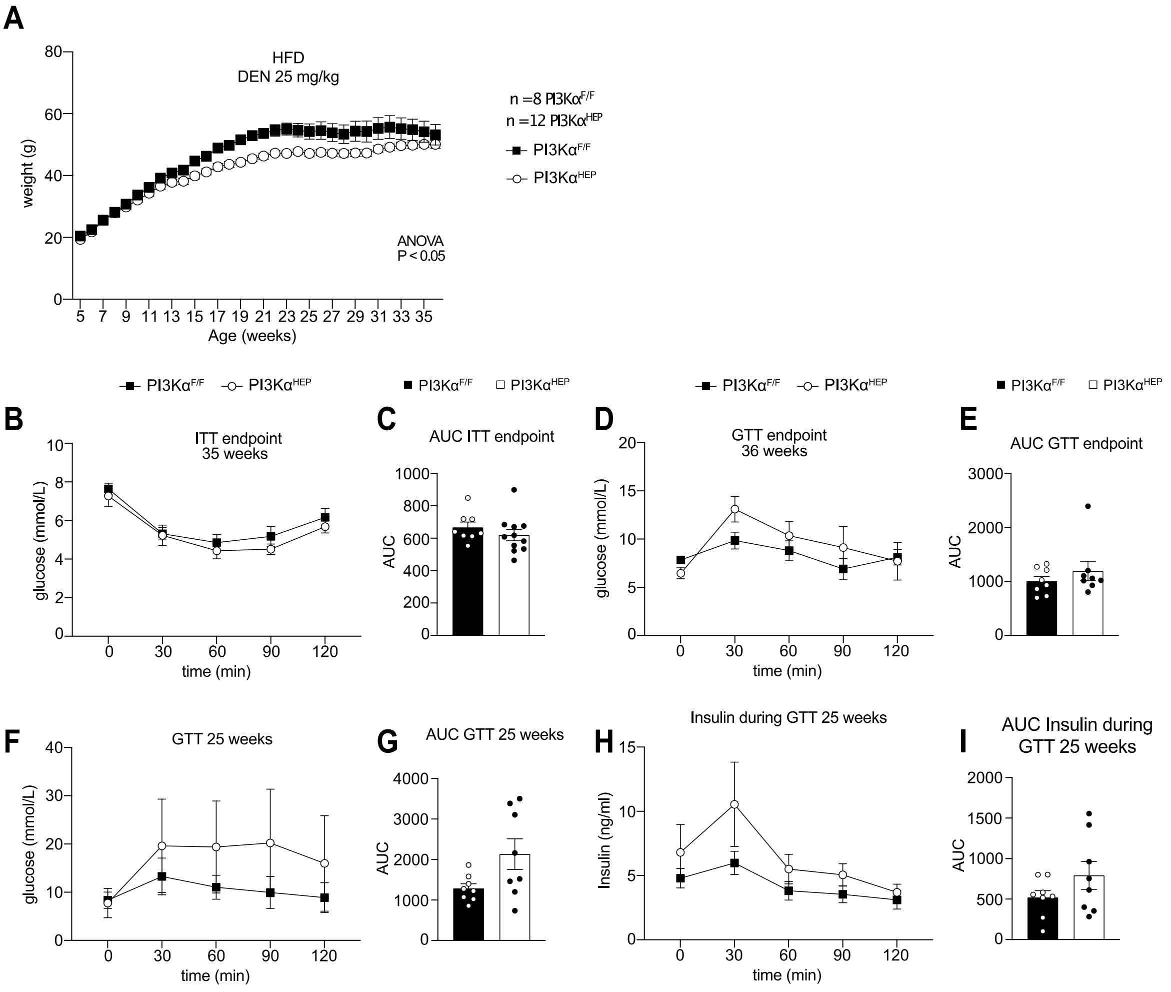

Fig. S3. (Related to Fig. 1) Loss of hepatocyte PI3Kα has a mild impact on growth and glucose homeostasis in DEN-injected mice fed an obesogenic HFD.

(A) Growth curve of PI3Kα$^{F/F}$ and PI3Kα^{HEP} mice kept on an HFD. (B) Insulin tolerance test (ITT) of the mice from A at the age of 35 weeks. (C) Area under the curves of the ITT in B. (D) Glucose tolerance test (GTT) of the mice in A at the age of 36 weeks. (E) Area under the curve of the GTT in D. (F) GTT of the mice in A at the age of 25 weeks. (G) Area under the curve of the GTT in F. (H) Serum insulin during the GTT in F. (I) Area under the curve of the insulin levels in H. n=8-12 mice for A, n=8 mice for B-I. Data are represented as means, and error bars indicate standard errors. Statistical analysis was performed using Mann-Whitney for C, E,D. I and two-way ANOVA for A,B, D, F, H.

Fig. S4

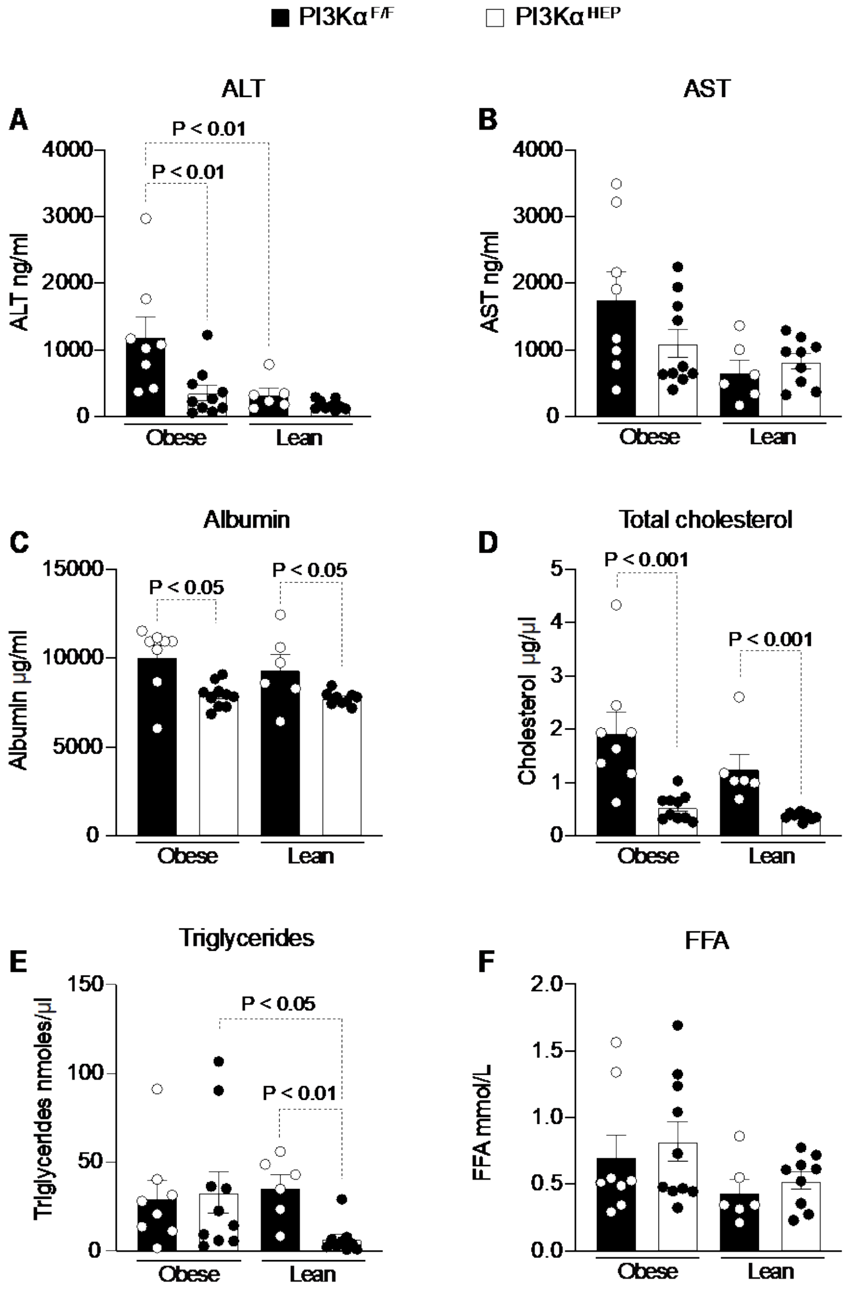

Fig. S3. (Related to Fig. 1) Liver enzymes and circulating lipids.

(A) Serum abundance of Alanine Aminotransferase (ALT). (B) Serum abundance of Aspartate Aminotransferase (AST). (C) Serum abundance of Albumin. (D) Serum abundance of total cholesterol. (E) Serum abundance of triglycerides. (F) Serum abundance of Free fatty acids (FFA). n=6-10 mice per group. Data are represented as means, and error bars indicate standard errors. Statistical analysis was performed using Mann-Whitney.

Fig. S5

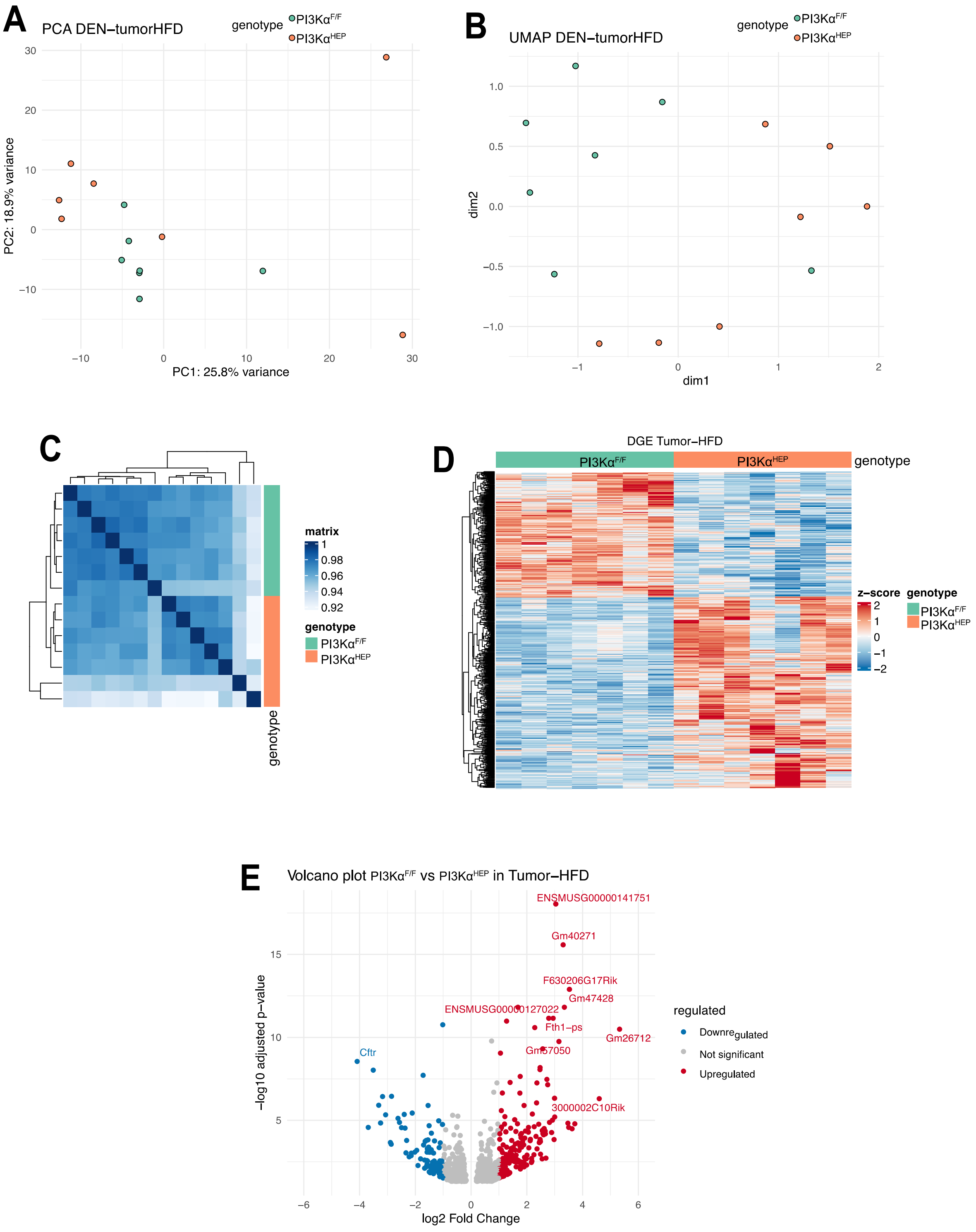
A
PCA DEN–tumorHFD
genotype
PI3Kα^F/F
PI3Kα^HEP
PC2: 18.9% variance
PC1: 25.8% variance
B
UMAP DEN–tumorHFD
dim2
dim1
C
matrix
1
0.98
0.96
0.94
0.92
genotype
D
DGE Tumor–HFD
z–score
E
Volcano plot PI3Kα^F/F vs PI3Kα^HEP in Tumor–HFD
ENSMUSG00000141751
Gm40271
F630206G17Rik
Gm47428
ENSMUSG00000127022
Fth1–ps
Gm26712
Gm57050
3000002C10Rik
Cftr
regulated
Downregulated
Not significant
Upregulated
–log10 adjusted p–value
log2 Fold Change

Fig. S5. (Related to Fig. 3) Dimensional analysis and differentially expressed genes in HCC from PI3K$\alpha^{F/F}$ and PI3Kα^{HEP} mice kept on an HFD.

(A) Principal component analysis (PCA) of mRNA Sequencing data from HCC of PI3K$\alpha^{F/F}$ and PI3Kα^{HEP} mice kept on an HFD from Figure 3. (B) Uniform Manifold Approximation and Projection (UMAP) analysis of mRNA sequencing data from HCC of PI3K$\alpha^{F/F}$ and PI3Kα^{HEP} mice kept on an HFD from Figure 3. (C) Clustered Spearman correlation matrix from the data above. (D) Heatmap showing z-scores of differentially expressed genes from HCC of PI3K$\alpha^{F/F}$ and PI3Kα^{HEP} mice kept on an HFD from Figure 3. Each row contains expression of one differentially expressed gene (n = 890, adj. p-value < 0.05). Each column represents the expression in one biological replicate (n = 7). (E) Volcano plot of the differentially expressed genes in D, marked as downregulated (log2 fold change < -1) or upregulated (log2 fold change > 1). The top 10 genes are labeled, based on the product of the absolute fold change and $-\log_{10}$ adj. p-value. Statistical analysis was performed using BH-adjusted Wald test
n=7 mice per genotype.

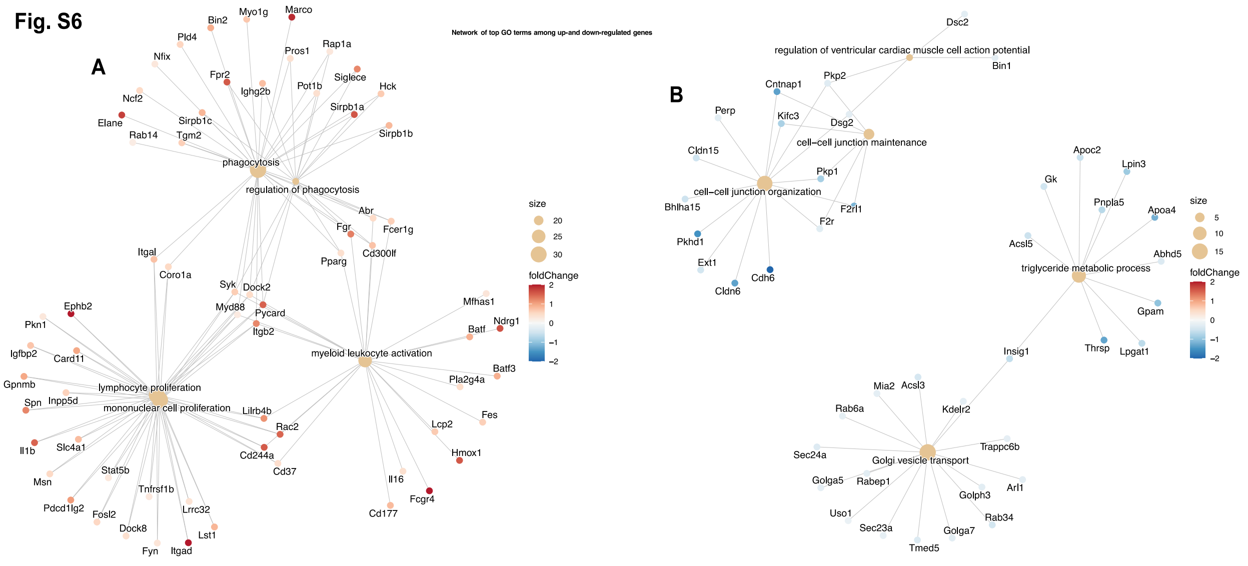

Fig. S6. (Related to Fig. 3) Network of top gene ontology terms

(A) Network of the most significant gene ontology terms of upregulated genes and their associated upregulated genes in HCC of PI3Kα^{HEP} mice kept on an HFD from Figure 3. Gene nodes are colored according to $\log_2$ fold change.

(B) Network of the most significant gene ontology terms of downregulated genes and their associated downregulated genes in HCC of PI3Kα^{HEP} mice kept on an HFD from Figure 3. Gene nodes are colored according to $\log_2$ fold change. n=7 mice per genotype.

Fig. S7

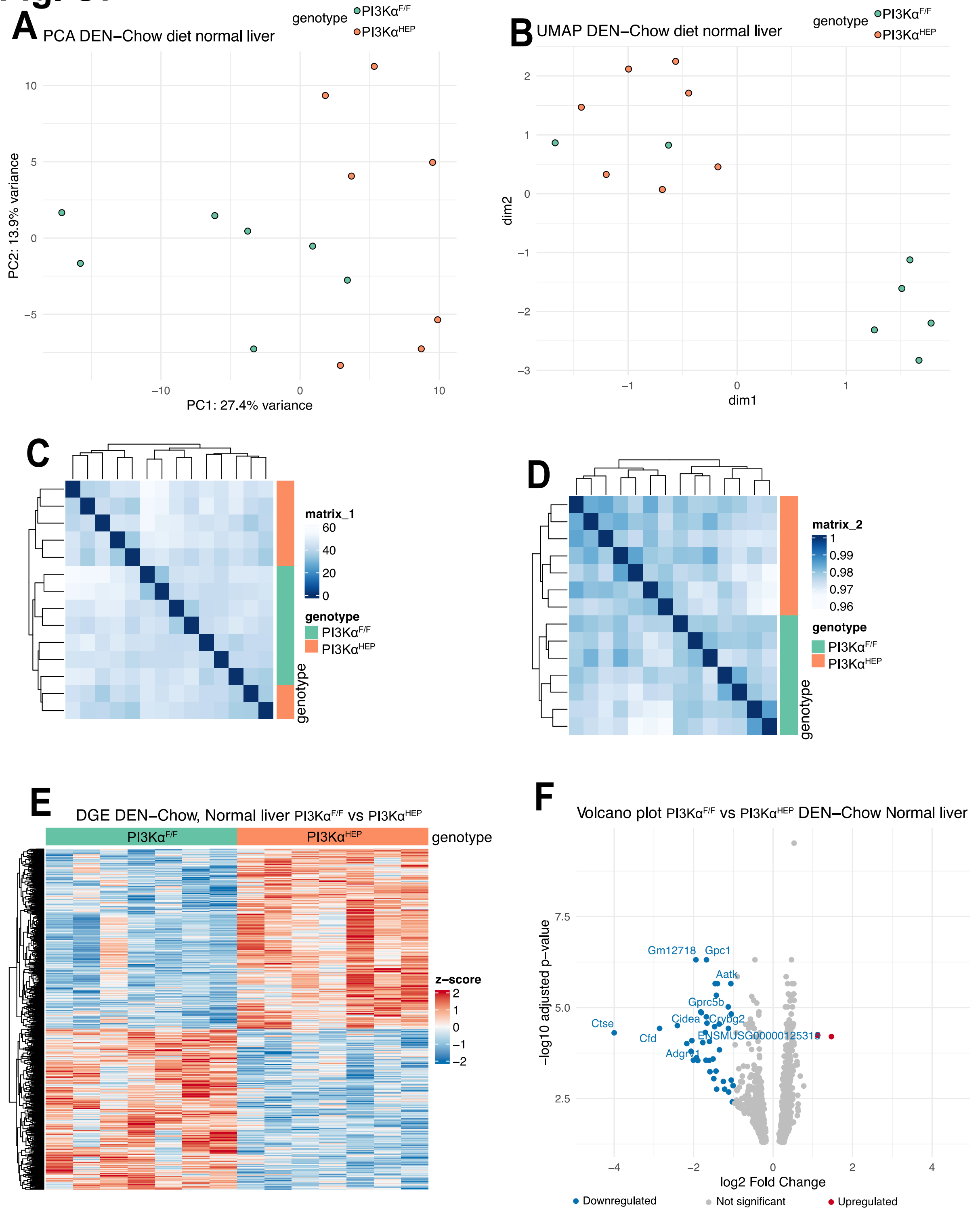

Fig. S7. (Related to Fig. 4) Dimensional analysis and differentially expressed genes in normal livers from PI3Kα$^{F/F}$ and PI3Kα^{HEP} mice kept on a chow diet.

(A) Principal component analysis (PCA) of mRNA Sequencing data from normal livers of PI3K$\alpha^{F/F}$ and PI3Kα^{HEP} mice kept on a chow diet from Figure 4.
(B) Uniform Manifold Approximation and Projection (UMAP) analysis of mRNA Sequencing data from above.
(C) Clustered Euclidian distance matrix from the data above.
(D) Clustered Spearman correlation matrix from the data above.
(E) Heatmap showing z-scores of differentially expressed genes from normal liver tissue of PI3K$\alpha^{F/F}$ and PI3Kα^{HEP} mice kept on chow diet from Figure 4. Each row contains expression of one differentially expressed gene (n = 1418, adj. p-value < 0.05). Each column represents the expression in one biological replicate (n = 7).
(F) Volcano plot of the differentially expressed genes in E, marked as downregulated (log2 fold change < -1) or upregulated (log2 fold change > 1). The top 10 genes are labeled, based on the product of the absolute fold change and -$\log_{10}$ adj. p-value.
Statistical analysis was performed using BH-adjusted Wald test. n=7 mice per genotype.

Fig. S8

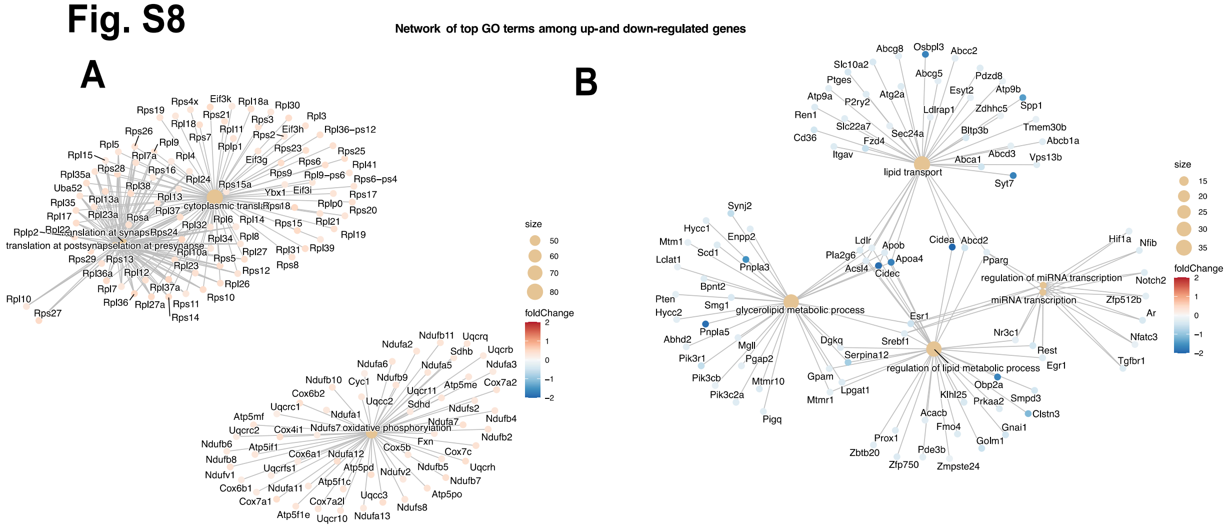

Fig. S8. (Related to Fig. 4) Network of top gene ontology terms (Chow diet)

(A) Network of the most significant gene ontology terms of upregulated genes and their associated upregulated genes in the normal liver of PI3Kα^{HEP} mice kept on a chow diet from Figure 4. Gene nodes are colored according to $\log_2$ fold change.

(B) Network of the most significant gene ontology terms of downregulated genes and their associated downregulated genes in the normal liver of PI3Kα^{HEP} mice kept on a chow diet from Figure 4. Gene nodes are colored according to $\log_2$ fold change.

n=7 mice per genotype.

Fig. S9

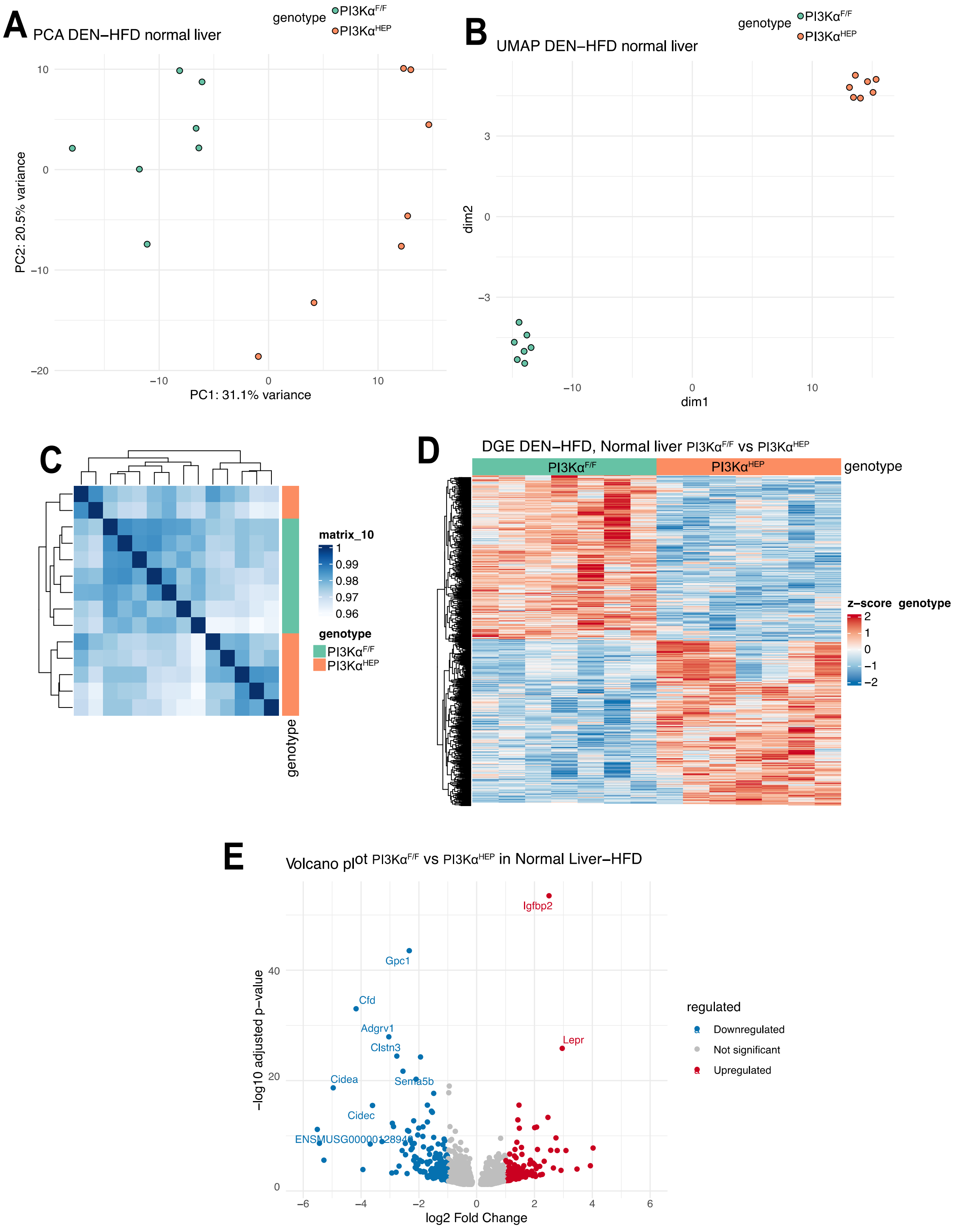

A
PCA DEN−HFD normal liver
genotype
PI3Kα F/F
PI3Kα HEP
PC2: 20.5% variance
PC1: 31.1% variance
B
UMAP DEN−HFD normal liver
dim2
dim1
C
matrix_10
genotype
D
DGE DEN−HFD, Normal liver PI3Kα F/F vs PI3Kα HEP
z−score
E
Volcano plot PI3Kα F/F vs PI3Kα HEP in Normal Liver−HFD
Igfbp2
Gpc1
Cfd
Adgrv1
Clstn3
Lepr
Cidea
Sema5b
Cidec
regulated
Downregulated
Not significant
Upregulated
−log10 adjusted p−value
log2 Fold Change

Fig. S9. (Related to Fig. 4) Dimensional analysis and differentially expressed genes in normal livers from PI3K$\alpha^{F/F}$ and PI3Kα^{HEP} mice kept on HFD

(A) Principal component analysis (PCA) of mRNA Sequencing data from normal livers of PI3K$\alpha^{F/F}$ and PI3Kα^{HEP} mice kept on an obesogenic HFD from Figure 4.

(B) Uniform Manifold Approximation and Projection (UMAP) analysis of mRNA Sequencing data from above.

(C) Clustered Spearman correlation matrix from the data above.

(D) Heatmap showing z-scores of differentially expressed genes from normal livers of PI3K$\alpha^{F/F}$ and PI3Kα^{HEP} mice kept on HFD from Figure 4. Each row contains expression of one differentially expressed gene (n = 1814, adj. p-value < 0.05). Each column represents the expression in one biological replicate (n = 7).

(E) Volcano plot of the differentially expressed genes in D, marked as downregulated (log2 fold change < -1) or upregulated (log2 fold change > 1). The top 10 genes are labeled, based on the product of the absolute fold change and $-\log_{10}$ adj. p-value. Statistical analysis was performed using BH-adjusted Wald test. n=7 mice per genotype.

Fig. S10

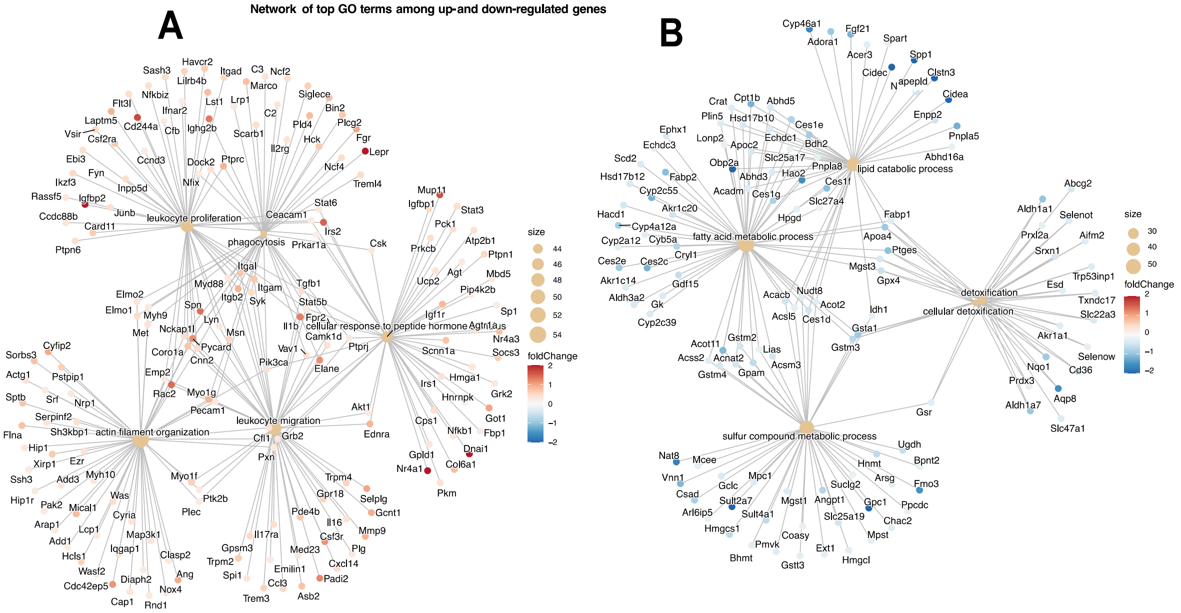

Fig. S10. (Related to Fig. 4) Network of top gene ontology terms (HFD)

(A) Network of the most significant gene ontology terms of upregulated genes and their associated upregulated genes in the normal liver of PI3Kα^{HEP} mice kept on HFD from Figure 4. Gene nodes are colored according to $\log_2$ fold change.

(B) Network of the most significant gene ontology terms of downregulated genes and their associated downregulated genes in the normal liver of PI3Kα^{HEP} mice kept on HFD from Figure 4. Gene nodes are colored according to $\log_2$ fold change.

n=7 mice per genotype.

Fig. S11

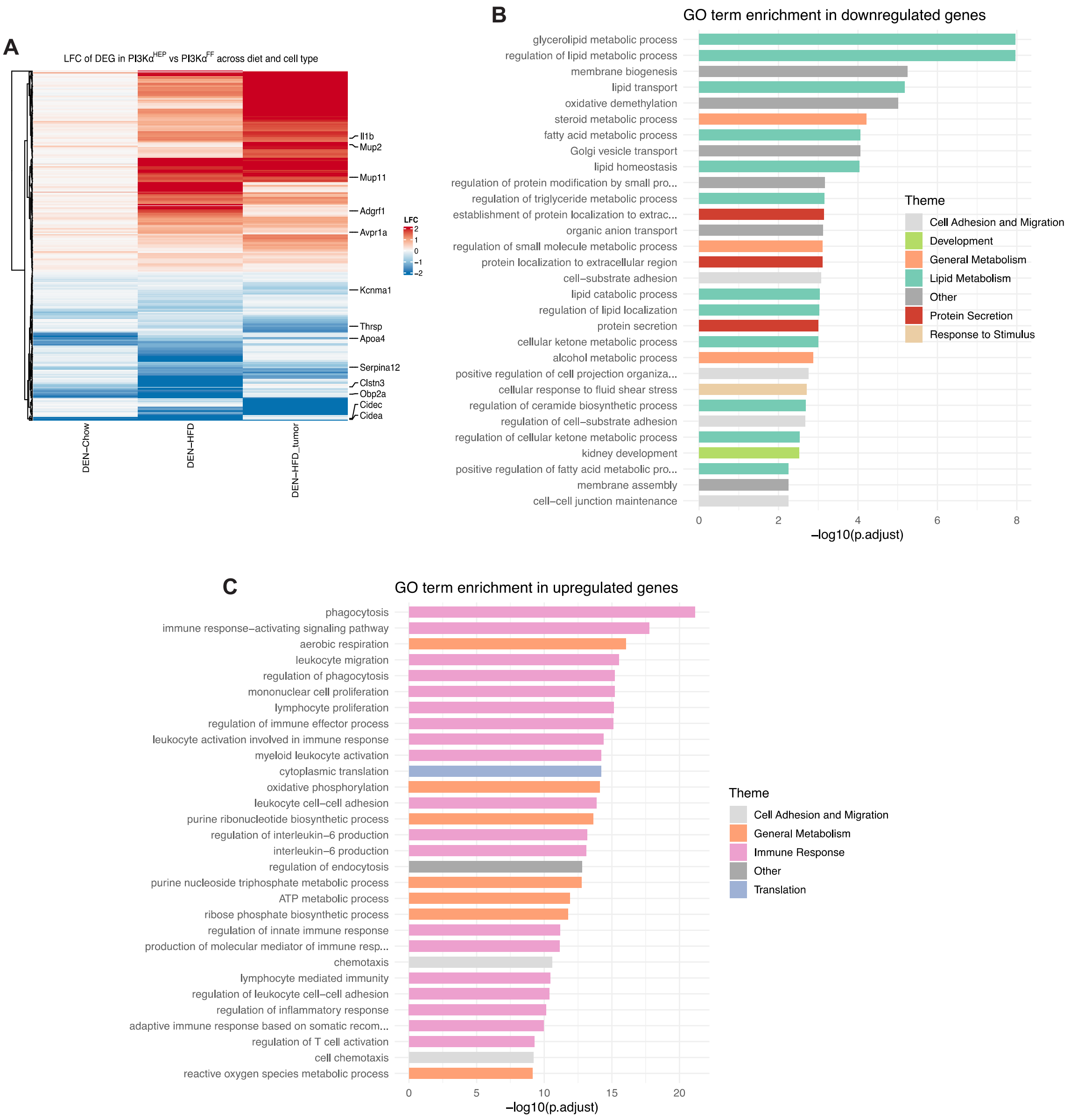

Fig. S11. (Related to Fig. 3, 4) Analysis of gene expression, all samples compared. (A) Heat map of differentially expressed genes in normal liver and tumor in chow and HFD. Log2 fold change (LFC) of expression in PI3Kα^{Hep} vs PI3Kα$^{F/F}$ across diet and tissue type. Significantly regulated genes (p-adj < 0.05) with a minimum effect size (|LFC| > 1) from the combined analysis were plotted. Genes which belong to the ‘regulation of lipid metabolism’ GO-term (GO:0019216) are labeled with their gene symbol.

(B) Gene ontology (GO) term enrichment analysis of all significantly downregulated genes (p-adj < 0.05) from the combined analysis in A. The top 30 most significant GO terms are shown, colored by overarching theme

(C) Gene ontology (GO) term enrichment analysis of all significantly upregulated genes (p-adj < 0.05) from the combined analysis in A. The top 30 most significant GO terms are shown, colored by overarching theme. Statistical analysis was performed using BH-adjusted hypergeometric test p-values.

Fig. S12

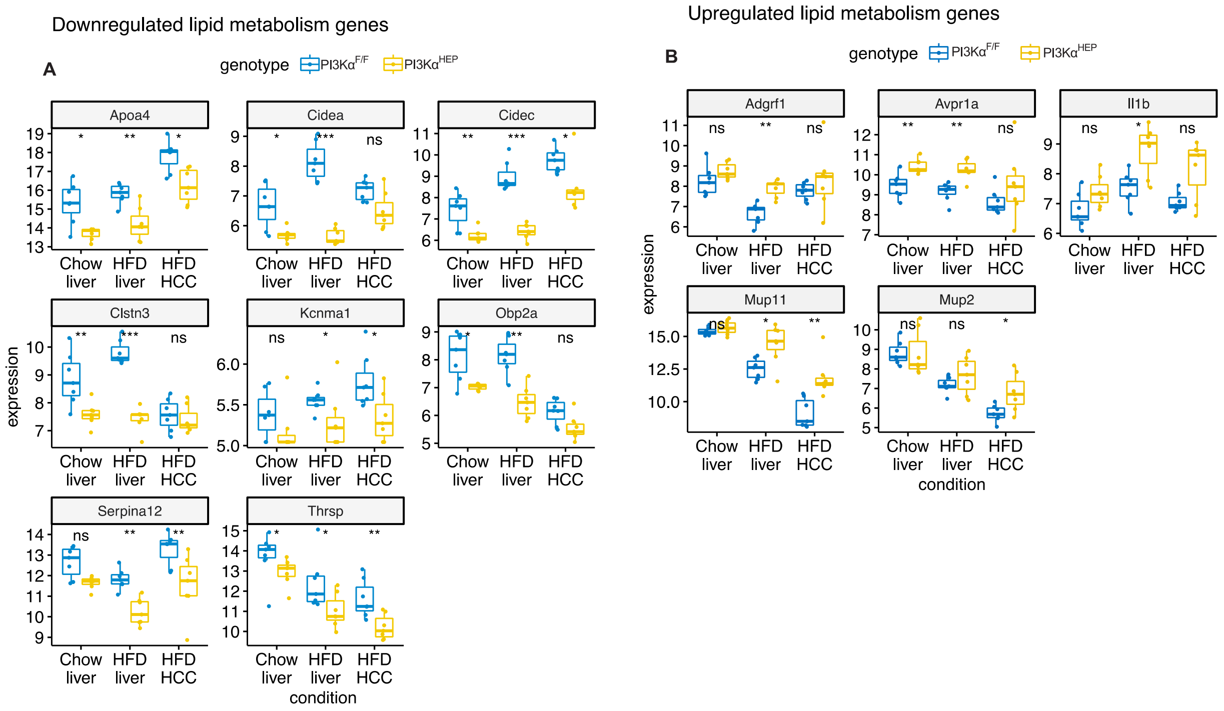

Fig. S12. (Related to Fig. 3, 4) Expression of significantly regulated lipid metabolism genes across diet and tissue types.

Expression of significantly regulated genes (p-adj < 0.05) with a minimum effect size (|LFC| > 1) across diet and tissue types from the combined analysis. Boxplots show the expression per condition in PI3Kα$^{F/F}$ (blue) and PI3Kα^{Hep} (yellow). The points show the expression for each of the biological replicates for the given gene (n=7). Statistical significance for the pairwise comparisons per condition shown above the boxplots (Wilcoxon rank sum test; ns = p ≥ 0.5, * = p < 0.05, ** = p < 0.01, *** = p < 0.001). Downregulated genes shown in A, and upregulated genes in B.

Fig. S13

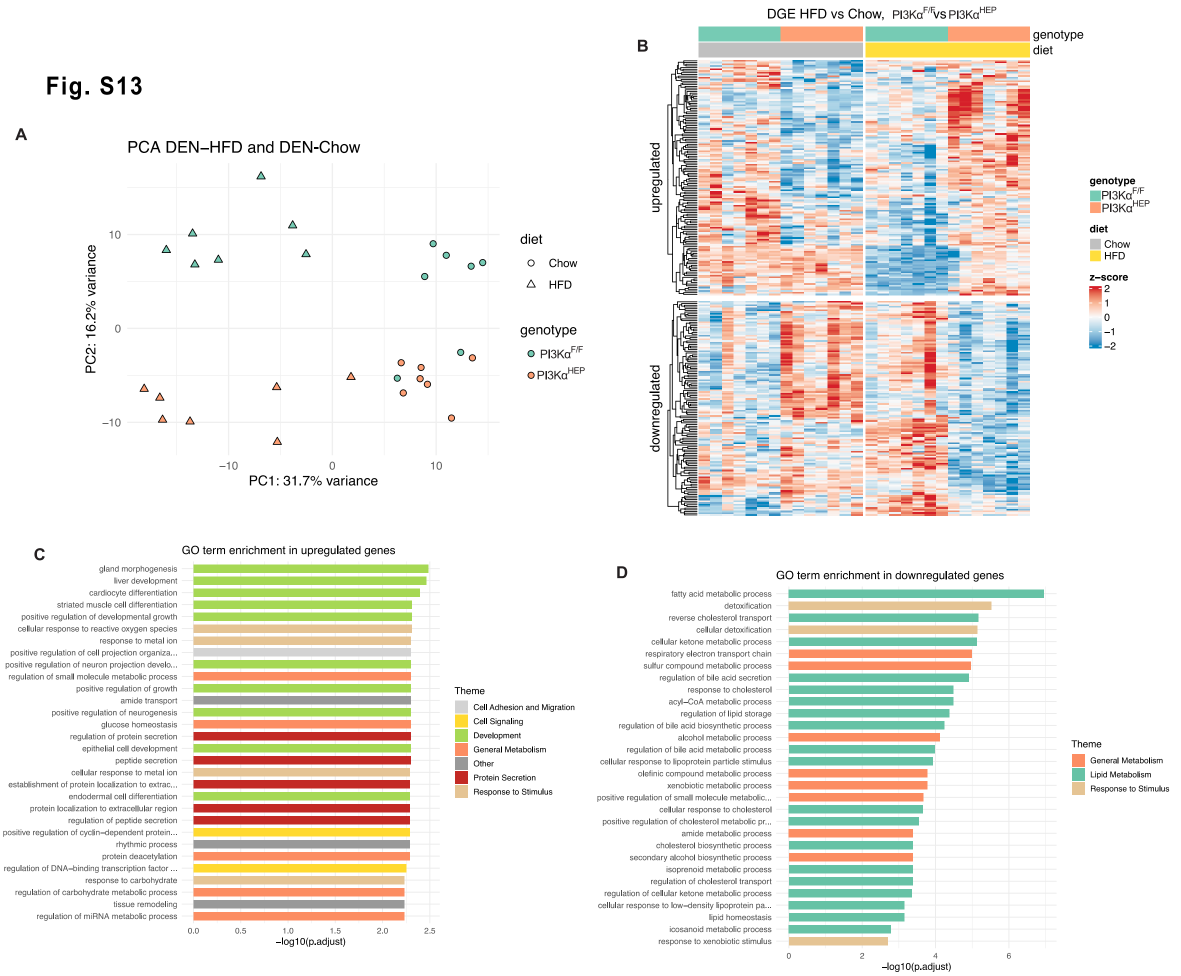

Fig. S13. (Related to Fig. 3, 4) Impact of diet on PI3Kα dependent gene regulation in normal liver.

(A) Principal component analysis of normal liver tissue from mice kept on chow diet (circles) or HFD (triangles). Points colored by genotype.

(B) Heatmap of differentially expressed genes dependent on PI3Kα and diet. Z-scores shown for all significant genes (p-adj < 0.05) with a minimum 1.5-fold up or downregulation in the interaction term.

(C) Gene ontology (GO) term enrichment analysis of the upregulated genes from B. The top 30 most significant GO terms are shown, colored by overarching theme.

(D) Gene ontology (GO) term enrichment analysis of the downregulated genes from B. The top 30 most significant GO terms are shown, colored by overarching theme. Statistical analysis by BH-adjusted hypergeometric test p-values.

Fig. S14

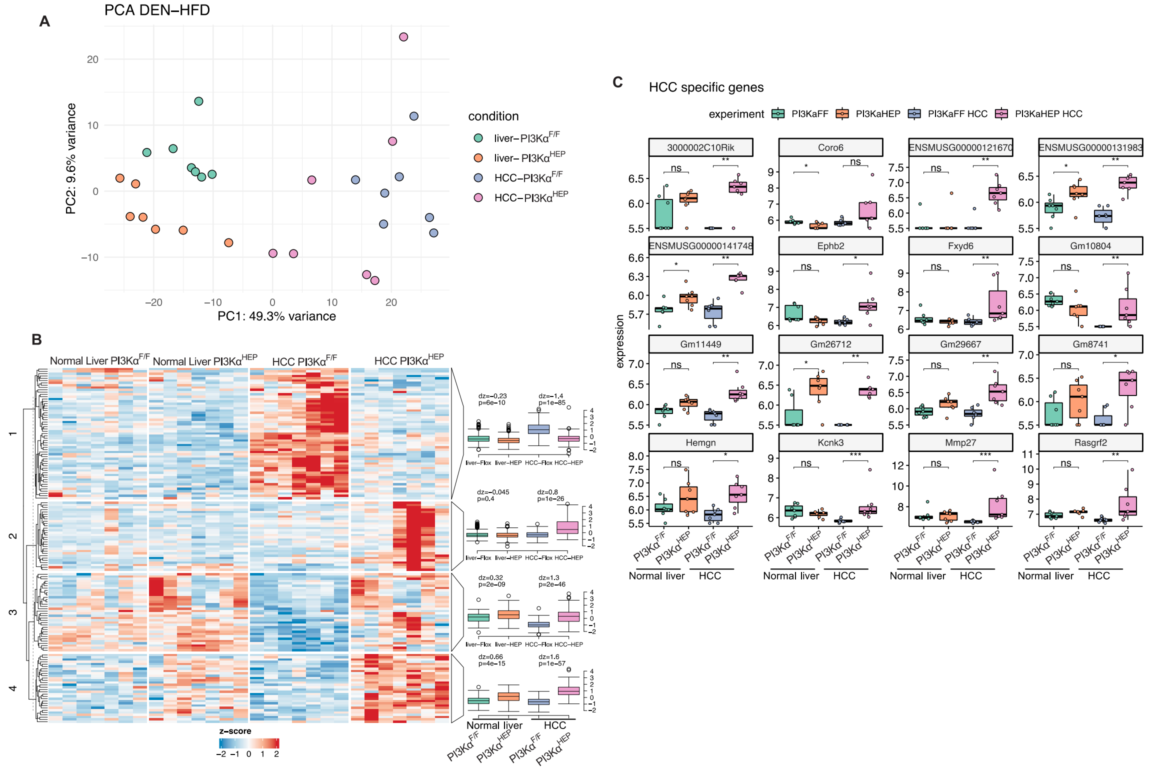

Fig. S14. (Related to Fig. 3, 4) Analysis of gene expression, comparison of normal liver and tumor from PI3Kα^{Hep} and PI3Kα$^{F/F}$ mice kept on HFD.

(A) Principal component analysis of normal liver tissue and HCC from mice kept on HFD, colored by condition.

(B) Heatmap of differentially expressed genes in response to PI3Kα in HCC but not normal liver tissue. Genes with p-adj < 0.05 and |LFC| > 1 in HCC, and p-adj > 0.1 or |LFC| < 0.2 in normal liver tissue were defined as specifically regulated in HCC. Z-scores were hierarchically clustered. The boxplots to the right show the z-scores for the cluster, for each of the conditions. The p-value (pairwise Wilcox test) and relative change in z-score (dz) are shown for the pairwise comparisons (normal liver PI3Kα$^{F/F}$ vs PI3Kα^{Hep} and HCC PI3Kα$^{F/F}$ vs PI3Kα^{Hep}).

(C) Expression of top 16 genes with the greatest LFC difference between normal liver and HCC from B. Statistical significance for the pairwise comparisons per condition shown above the boxplots (Wilcoxon rank sum test; ns = p ≥ 0.5, * = p < 0.05, ** = p < 0.01, *** = p < 0.001).
